# Supplementary figures and images for: Adsorption of phosphate over a novel magnesium-loaded sludge-based biochar
Source: PLoS One. 2024 Apr 16;19(4):e0301986. doi: 10.1371/journal.pone.0301986 (PMC11020854; doi:10.1371/journal.pone.0301986)

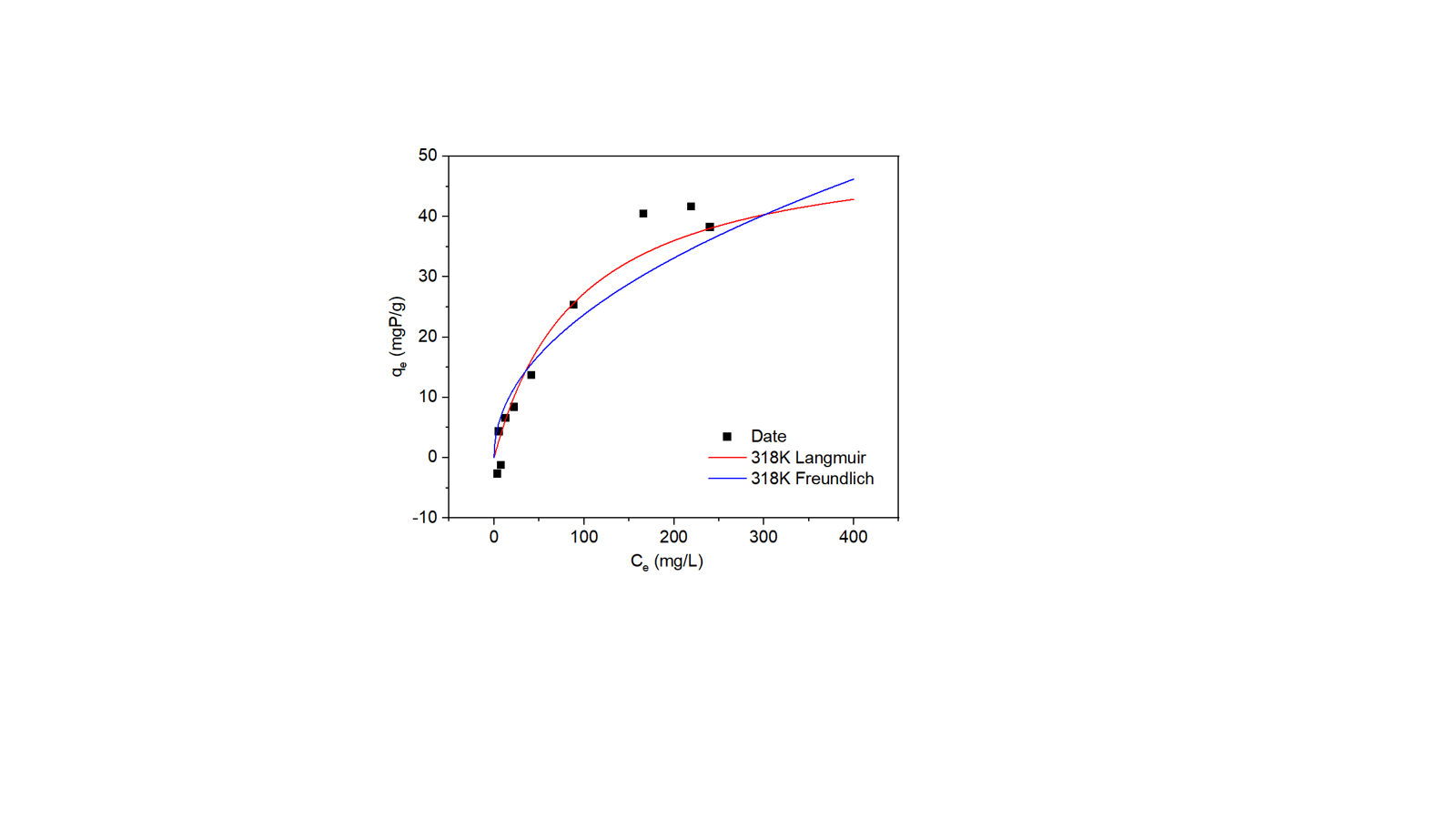

Supplement: S1 Fig — (TIF) [file pone.0301986.s001.tif]

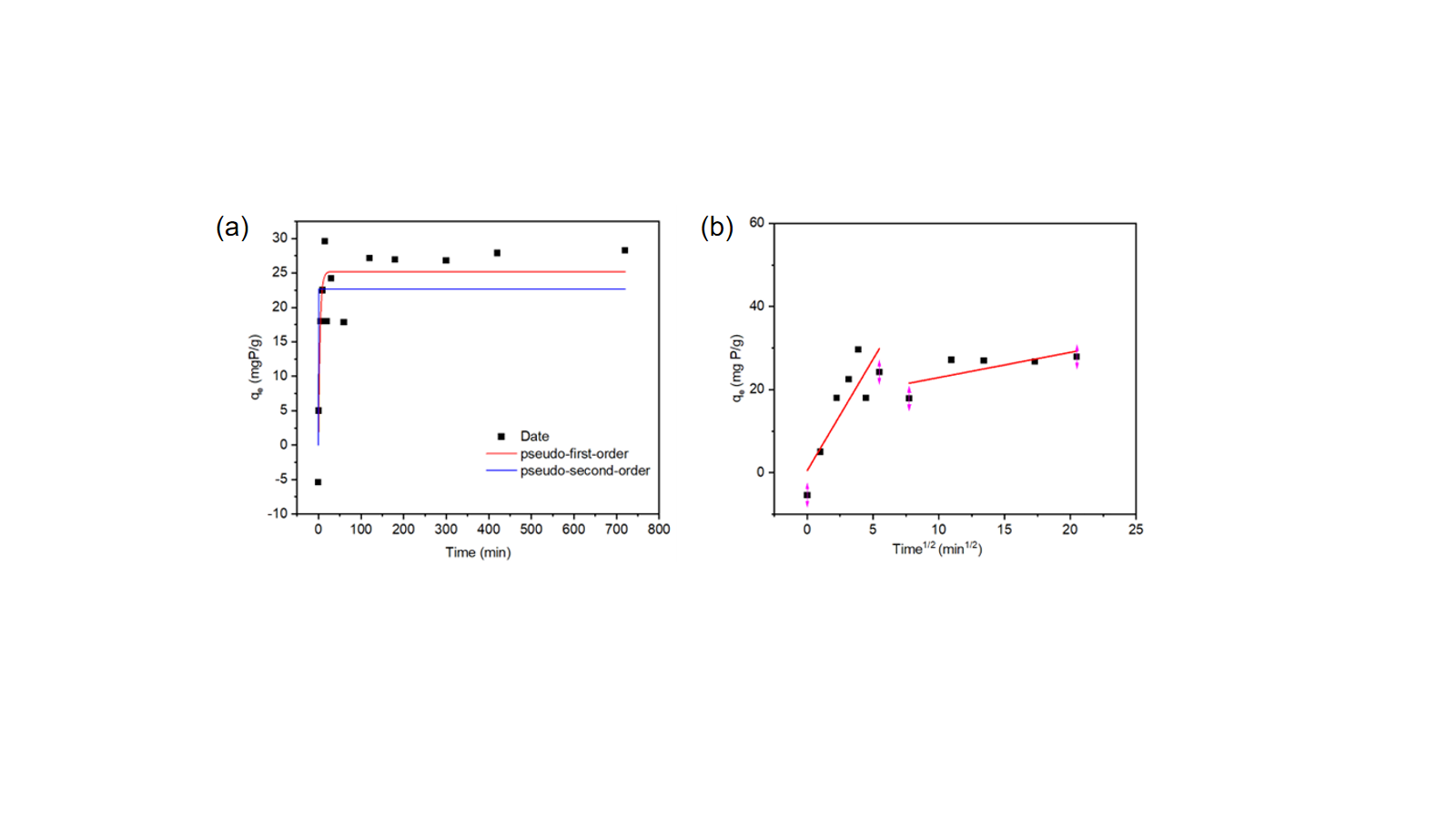

Supplement: S2 Fig — (a) Kinetic curves of SBC and (b) particle diffusion model fitting of SBC. (TIF) [file pone.0301986.s002.tif]

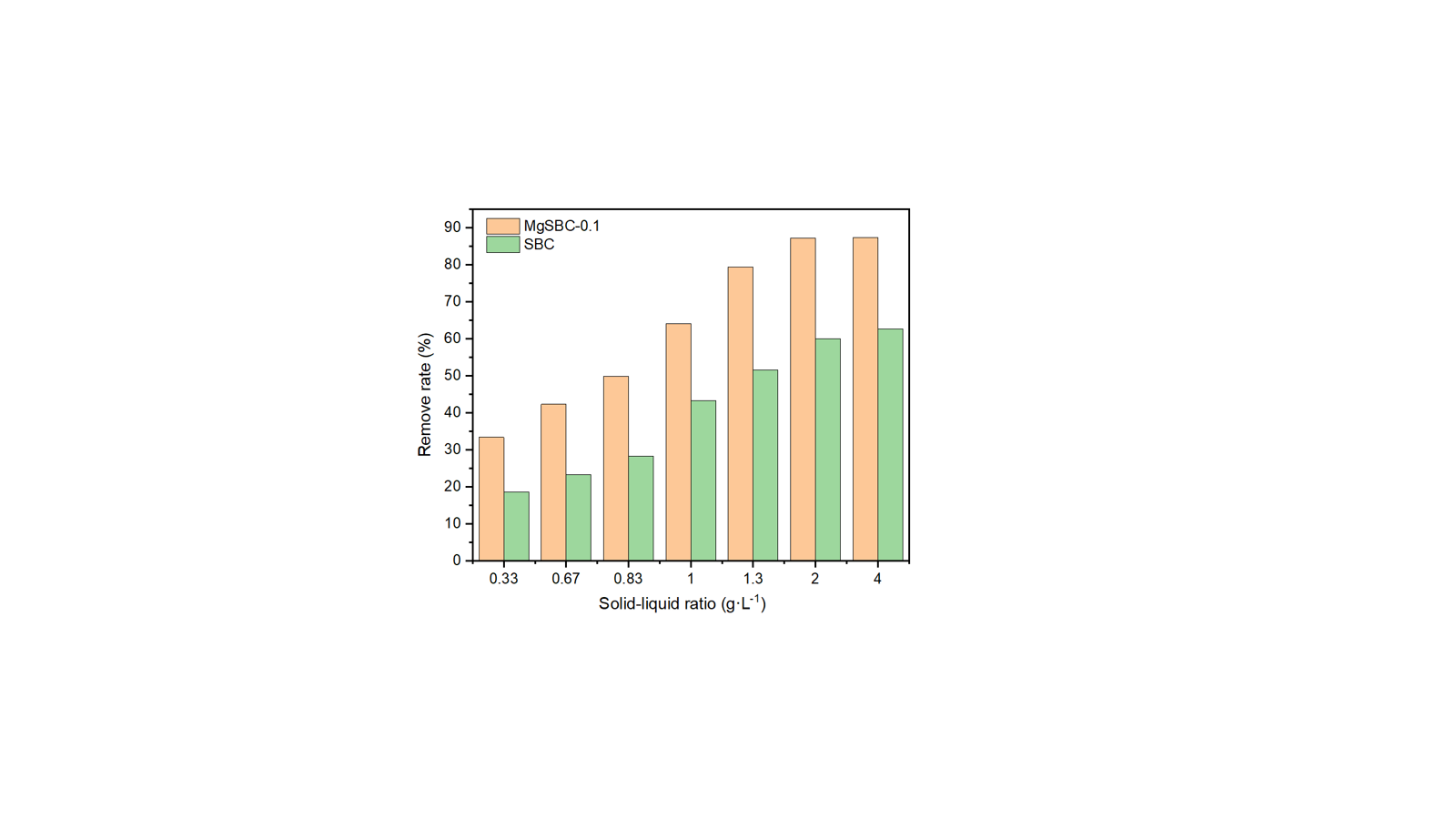

Supplement: S3 Fig — (TIF) [file pone.0301986.s003.tif]

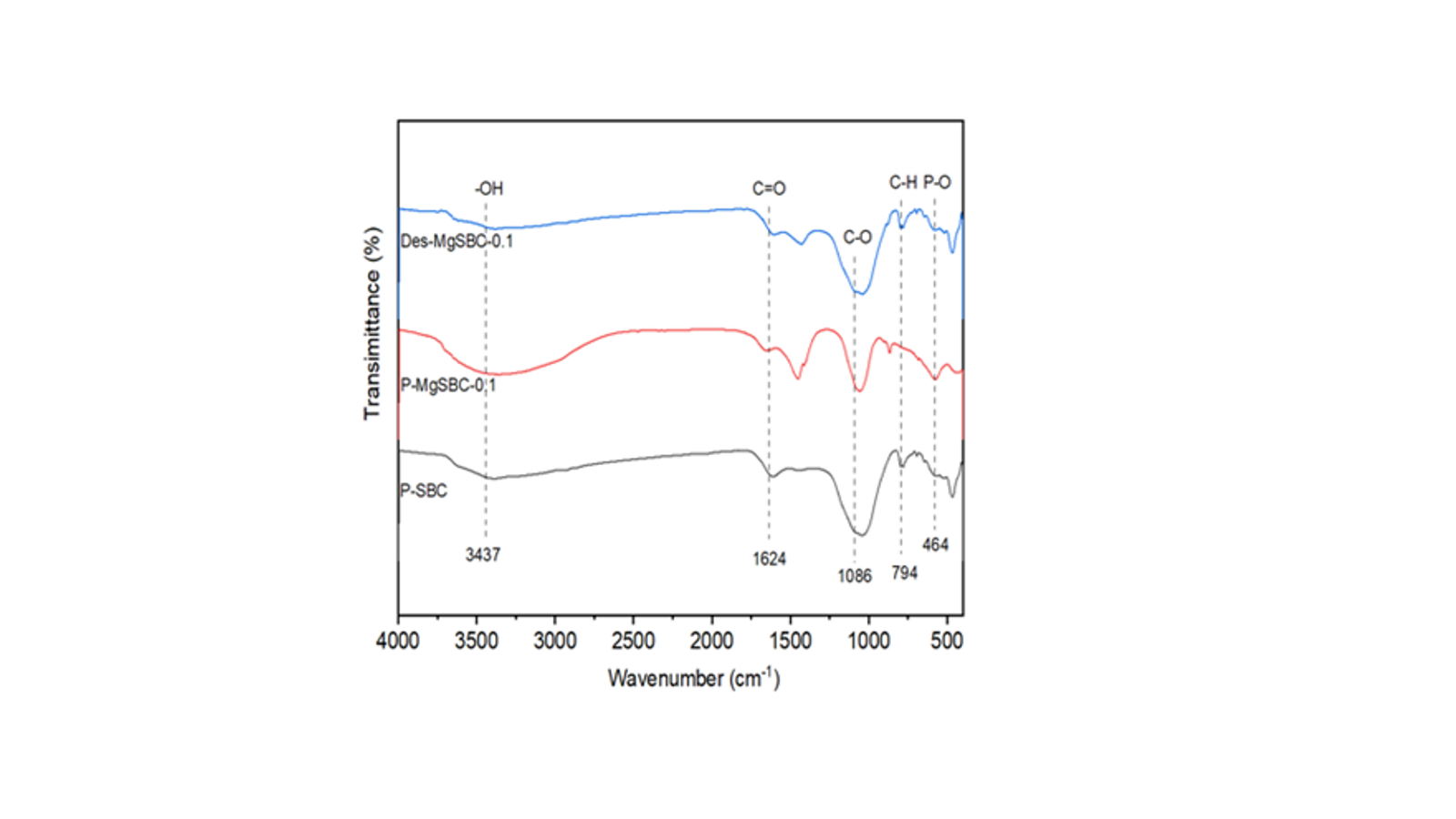

Supplement: S4 Fig — (TIF) [file pone.0301986.s004.tif]

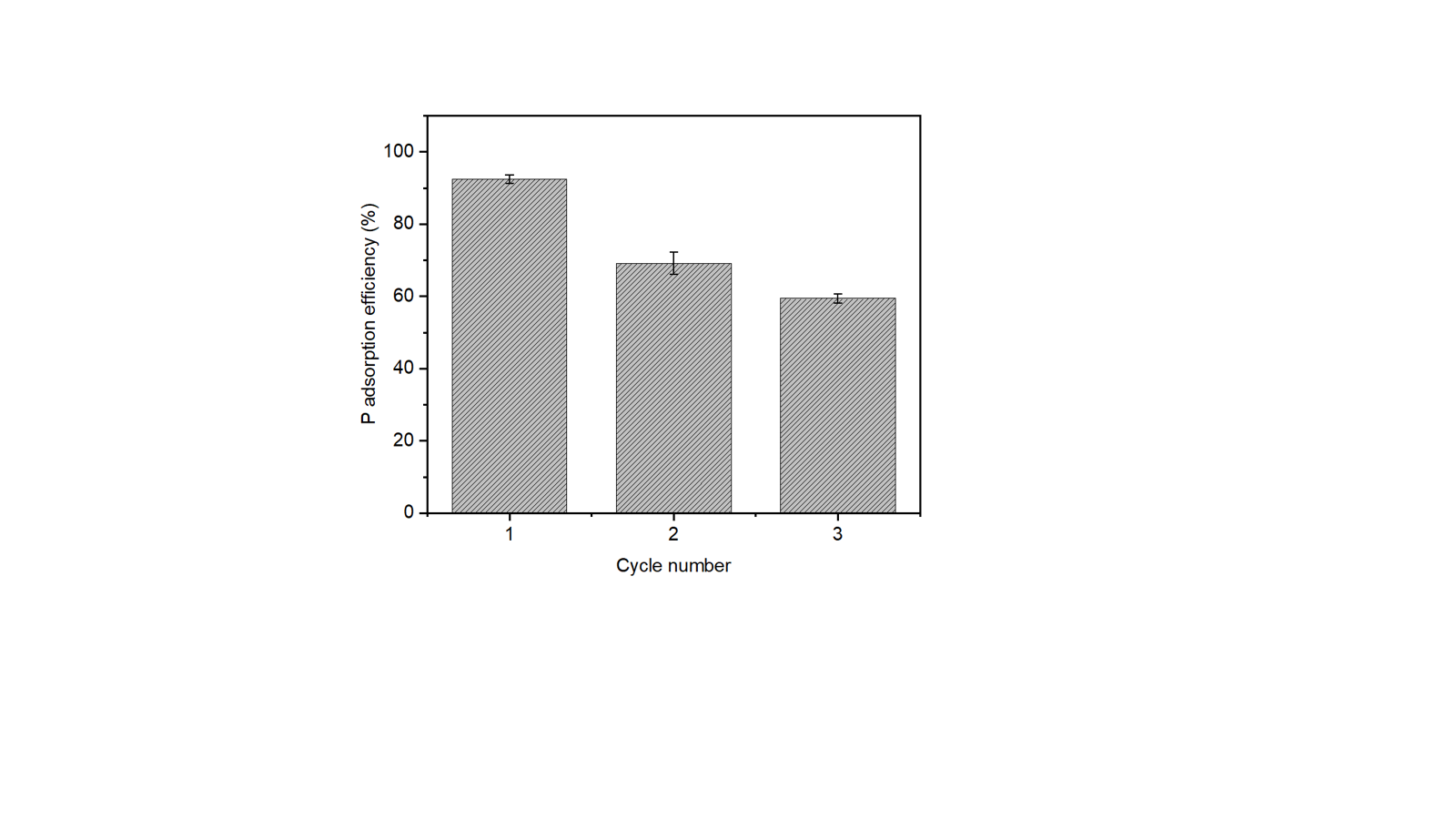

Supplement: S5 Fig — (TIF) [file pone.0301986.s005.tif]

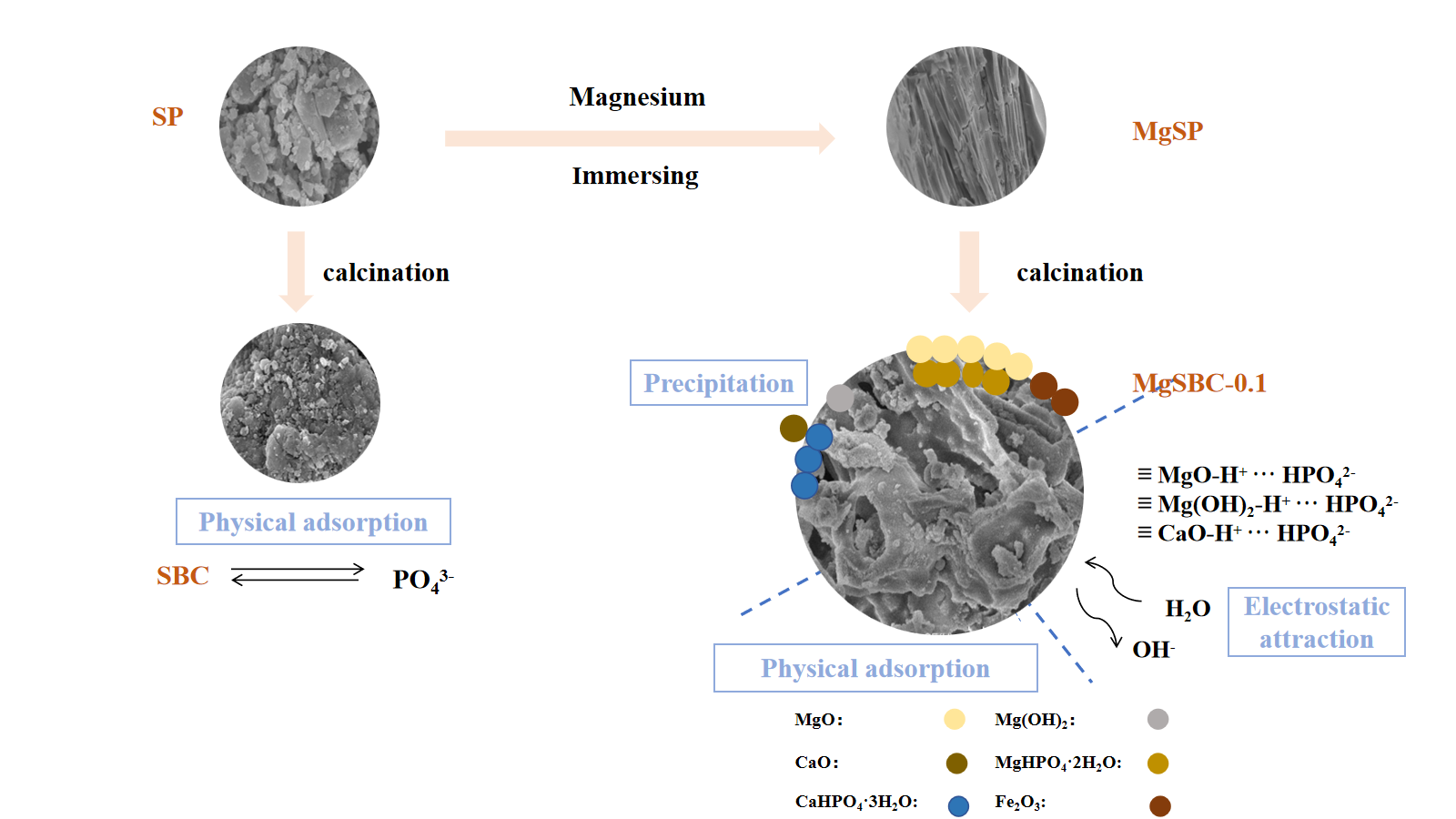

Supplement: S1 Graphical abstract — (TIF) [file pone.0301986.s006.tif]
