# Supplementary material for: Adsorption of phosphate over a novel magnesium-loaded sludge-based biochar
Source: PLoS One. 2024 Apr 16;19(4):e0301986. doi: 10.1371/journal.pone.0301986 (PMC11020854; doi:10.1371/journal.pone.0301986)

Sample: zmb-bjh
Operator:
Submitter:
File: E:\ASAP 2460\data\2307267899-1.SMP

Started:	2023/7/30 8:14:01	Analysis adsorptive:	N2
Completed:	2023/7/30 11:44:14	Analysis bath temp.:	-195.850 °C
Report time:	2023/7/30 19:22:42	Thermal correction:	No
Sample mass:	0.1315 g	Ambient free space:	17.2326 cm³ Measured
Analysis free space:	48.6379 cm³	Equilibration interval:	6 s
Low pressure dose:	None	Sample density:	1.000 g/cm³
Automatic degas:	No		

Summary Report

Surface Area

Single point surface area at P/Po = 0.259784074: 15.9502 m²/g

BET Surface Area: 16.7594 m²/g

Langmuir Surface Area: 59.1124 m²/g

t-Plot external surface area: 17.0231 m²/g

BJH Adsorption cumulative surface area of pores between 3.7602 nm and 300.0000 nm diameter: 11.4870 m²/g

BJH Desorption cumulative surface area of pores between 2.3123 nm and 21.7646 nm diameter: 16.9527 m²/g

D-H Adsorption cumulative surface area of pores between 1.7000 nm and 300.0000 nm diameter: 16.5068 m²/g

D-H Desorption cumulative surface area of pores between 1.7000 nm and 300.0000 nm diameter: 20.0117 m²/g

Pore Volume

Single point adsorption total pore volume of pores less than 43.5150 nm diameter at P/Po = 0.953818751: 0.037230 cm³/g

Single point desorption total pore volume of pores less than 40.3122 nm diameter at P/Po = 0.950000000: 0.043011 cm³/g

t-Plot micropore volume: -0.000437 cm³/g

BJH Adsorption cumulative volume of pores between 3.7602 nm and 300.0000 nm diameter: 0.065372 cm³/g

BJH Desorption cumulative volume of pores between 2.3123 nm and 21.7646 nm diameter: 0.032673 cm³/g

D-H Adsorption cumulative volume of pores between 1.7000 nm and 300.0000 nm diameter: 0.067628 cm³/g

D-H Desorption cumulative volume of pores between 1.7000 nm and 300.0000 nm diameter: 0.068652 cm³/g

Sample:	zmb-bjh
Operator:	
Submitter:	
File:	E:\ASAP 2460\data\2307267899-1.SMP

Started:	2023/7/30 8:14:01	Analysis adsorptive:	N2
Completed:	2023/7/30 11:44:14	Analysis bath temp.:	-195.850 °C
Report time:	2023/7/30 19:22:42	Thermal correction:	No
Sample mass:	0.1315 g	Ambient free space:	17.2326 cm³ Measured
Analysis free space:	48.6379 cm³	Equilibration interval:	6 s
Low pressure dose:	None	Sample density:	1.000 g/cm³
Automatic degas:	No		

Pore Size

Adsorption average pore diameter (4V/A by BET):	8.8857 nm
Desorption average pore diameter (4V/A by BET):	10.2655 nm
BJH Adsorption average pore diameter (4V/A):	22.7637 nm
BJH Desorption average pore diameter (4V/A):	7.7091 nm
D-H Adsorption average pore diameter (4V/A):	16.3880 nm
D-H Desorption average pore diameter (4V/A):	13.7224 nm

Horvath-Kawazoe

Maximum pore volume at P/Po = 0.539704478:	0.010995 cm³/g
Median pore width:	0.8454 nm

Sample: zmb-bjh
Operator:
Submitter:
File: E:\ASAP 2460\data\2307267899-1.SMP

Started: 2023/7/30 8:14:01
Completed: 2023/7/30 11:44:14
Report time: 2023/7/30 19:22:42
Sample mass: 0.1315 g
Analysis free space: 48.6379 cm³
Low pressure dose: None
Automatic degas: No

Analysis adsorptive: N2
Analysis bath temp.: -195.850 °C
Thermal correction: No
Ambient free space: 17.2326 cm³ Measured
Equilibration interval: 6 s
Sample density: 1.000 g/cm³

Isotherm Tabular Report

Relative Pressure (P/Po)	Absolute Pressure (mmHg)	Quantity Adsorbed (cm³/g STP)	Elapsed Time (h:min)	Saturation Pressure (mmHg)
			00:58	766.490417
0.053930052	41.329353	3.1600	01:10	766.351074
0.084758177	64.957603	3.5196	01:12	766.387451
0.120039808	91.985016	3.8597	01:13	766.287598
0.154915522	118.755768	4.1585	01:14	766.584045
0.189956615	145.610001	4.4369	01:16	766.543457
0.224870737	172.389099	4.6965	01:18	766.614197
0.259784074	199.145248	4.9506	01:19	766.579895
0.294843755	226.056854	5.2007	01:21	766.700500
0.329917816	252.989334	5.4535	01:22	766.825317
0.364841948	279.779907	5.7044	01:24	766.852356
0.399799014	306.597626	5.9759	01:26	766.879395
0.434840086	333.506104	6.2397	01:27	766.962646
0.469902412	360.331116	6.5163	01:28	766.821167
0.504801271	387.116455	6.7987	01:30	766.869019
0.539704478	413.955048	7.1080	01:31	767.003174
0.574962967	440.897949	7.4320	01:33	766.828430
0.609783347	467.549744	7.8019	01:34	766.747314
0.644597870	494.403931	8.2158	01:36	766.995911
0.679837373	521.340576	8.6867	01:37	766.860718
0.714576906	548.040405	9.2504	01:39	766.943909
0.749587645	574.947815	9.9207	01:41	767.018799
0.784670855	601.763428	10.7535	01:42	766.899170
0.819187435	628.333862	11.8231	01:44	767.020874
0.854289095	655.051453	13.2958	01:45	766.779602
0.888358118	681.171143	15.4286	01:47	766.775391
0.922666989	707.391052	18.6508	01:50	766.680786
0.954316659	731.768372	24.3306	01:53	766.798279
0.991412909	760.393127	44.1982	01:57	766.979248
0.962893528	738.410217	32.0258	02:01	766.865906
0.921173252	706.448914	23.7310	02:04	766.901245
0.877713075	673.268982	19.6369	02:07	767.071838
0.839691026	644.047424	17.2764	02:08	767.005249
0.803483337	616.270935	15.5616	02:10	766.999023
0.785684110	602.626282	14.8366	02:12	767.008362
0.750587511	575.624939	13.6167	02:13	766.899170
0.715155811	548.641357	12.5769	02:14	767.163391
0.680465656	521.956116	11.6843	02:16	767.057251
0.645370716	495.010132	10.8826	02:17	767.016724
0.610355385	468.108978	10.1761	02:19	766.944946
0.575092574	441.186951	9.5439	02:21	767.158142
0.540172471	414.437042	8.9782	02:22	767.230957

Sample: zmb-bjh
Operator:
Submitter:
File: E:\ASAP 2460\data\2307267899-1.SMP

Started: 2023/7/30 8:14:01
Completed: 2023/7/30 11:44:14
Report time: 2023/7/30 19:22:42
Sample mass: 0.1315 g
Analysis free space: 48.6379 cm³
Low pressure dose: None
Automatic degas: No

Analysis adsorptive: N2
Analysis bath temp.: -195.850 °C
Thermal correction: No
Ambient free space: 17.2326 cm³ Measured
Equilibration interval: 6 s
Sample density: 1.000 g/cm³

Isotherm Tabular Report

Relative Pressure (P/Po)	Absolute Pressure (mmHg)	Quantity Adsorbed (cm³/g STP)	Elapsed Time (h:min)	Saturation Pressure (mmHg)
0.505482999	387.630829	8.4643	02:23	766.852356
0.471716115	361.813660	7.1522	02:26	767.015686
0.435228733	333.791962	6.4174	02:27	766.934570
0.381282652	292.443817	6.0000	02:29	767.000061
0.345993976	265.451904	5.7314	02:30	767.215393
0.330166836	253.265793	5.6000	02:32	767.084290
0.295034491	226.295776	5.3545	02:33	767.014648
0.260074116	199.539627	5.0950	02:35	767.241394
0.224938659	172.567505	4.8550	02:37	767.175842
0.190003575	145.767532	4.5983	02:38	767.183105
0.154966061	118.877838	4.3218	02:40	767.121765
0.119898447	91.986061	4.0396	02:41	767.199768
0.085017326	65.237213	3.7071	02:43	767.340210
0.050057044	38.400734	3.3133	02:45	767.139465

Sample: zmb-bjh
Operator:
Submitter:
File: E:\ASAP 2460\data\2307267899-1.SMP

Started:	2023/7/30 8:14:01	Analysis adsorptive:	N2
Completed:	2023/7/30 11:44:14	Analysis bath temp.:	-195.850 °C
Report time:	2023/7/30 19:22:42	Thermal correction:	No
Sample mass:	0.1315 g	Ambient free space:	17.2326 cm³ Measured
Analysis free space:	48.6379 cm³	Equilibration interval:	6 s
Low pressure dose:	None	Sample density:	1.000 g/cm³
Automatic degas:	No		

Isotherm Linear Plot

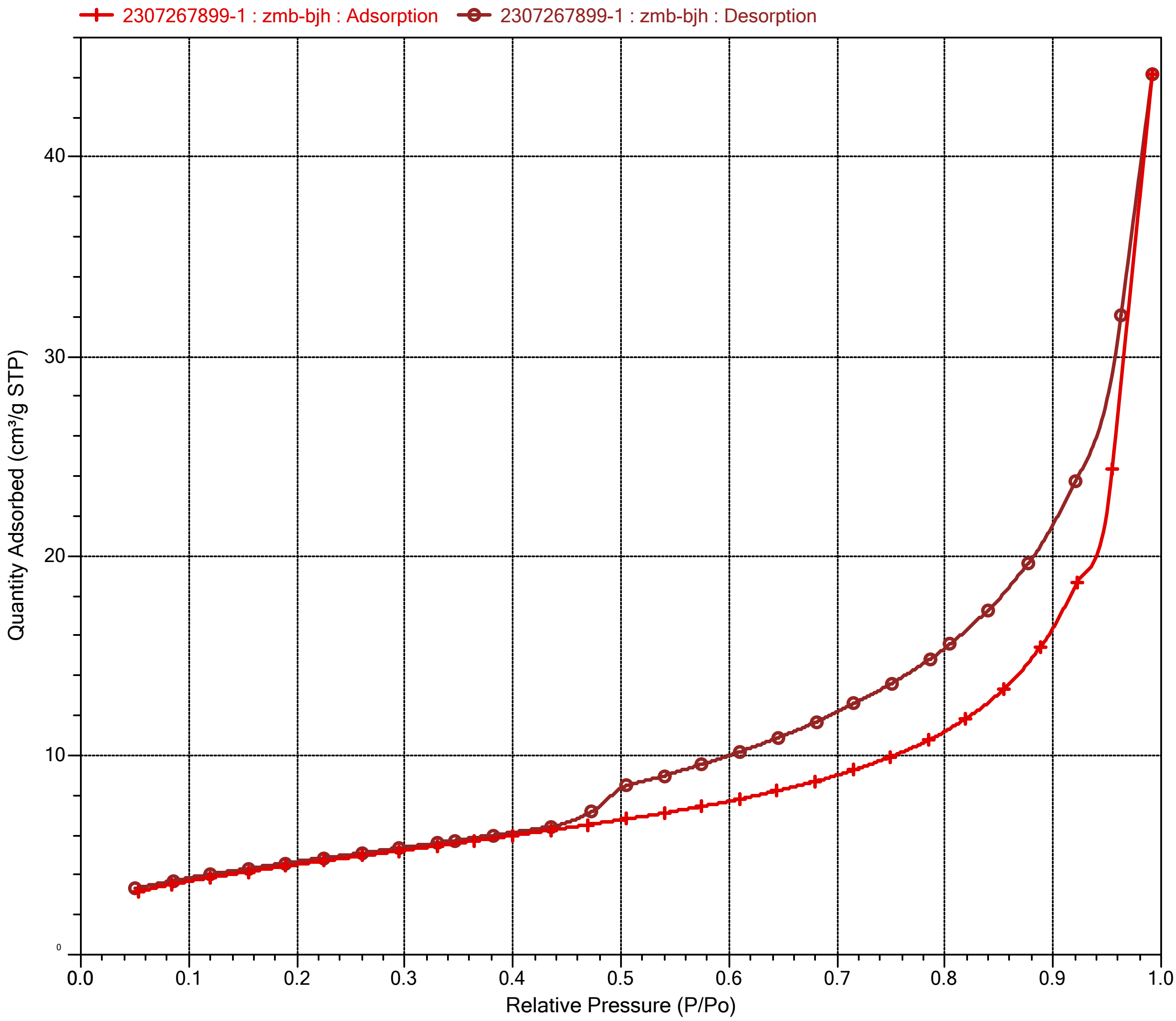

Sample: zmb-bjh
Operator:
Submitter:
File: E:\ASAP 2460\data\2307267899-1.SMP

Started:	2023/7/30 8:14:01	Analysis adsorptive:	N2
Completed:	2023/7/30 11:44:14	Analysis bath temp.:	-195.850 °C
Report time:	2023/7/30 19:22:42	Thermal correction:	No
Sample mass:	0.1315 g	Ambient free space:	17.2326 cm³ Measured
Analysis free space:	48.6379 cm³	Equilibration interval:	6 s
Low pressure dose:	None	Sample density:	1.000 g/cm³
Automatic degas:	No		

Isotherm Log Plot

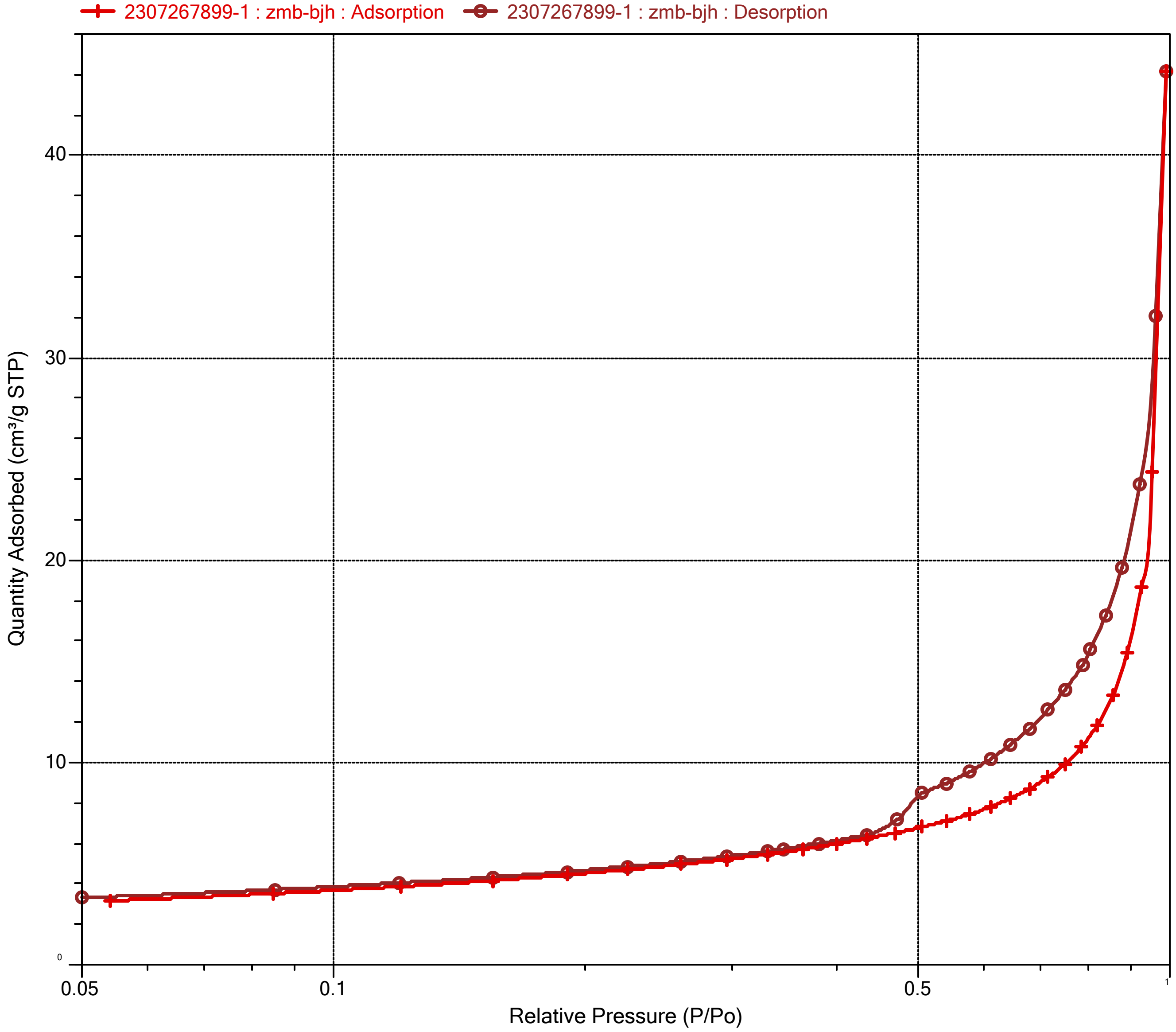

Sample: zmb-bjh
Operator:
Submitter:
File: E:\ASAP 2460\data\2307267899-1.SMP

Started:	2023/7/30 8:14:01	Analysis adsorptive:	N2
Completed:	2023/7/30 11:44:14	Analysis bath temp.:	-195.850 °C
Report time:	2023/7/30 19:22:42	Thermal correction:	No
Sample mass:	0.1315 g	Ambient free space:	17.2326 cm³ Measured
Analysis free space:	48.6379 cm³	Equilibration interval:	6 s
Low pressure dose:	None	Sample density:	1.000 g/cm³
Automatic degas:	No		

Isotherm Linear Absolute Plot

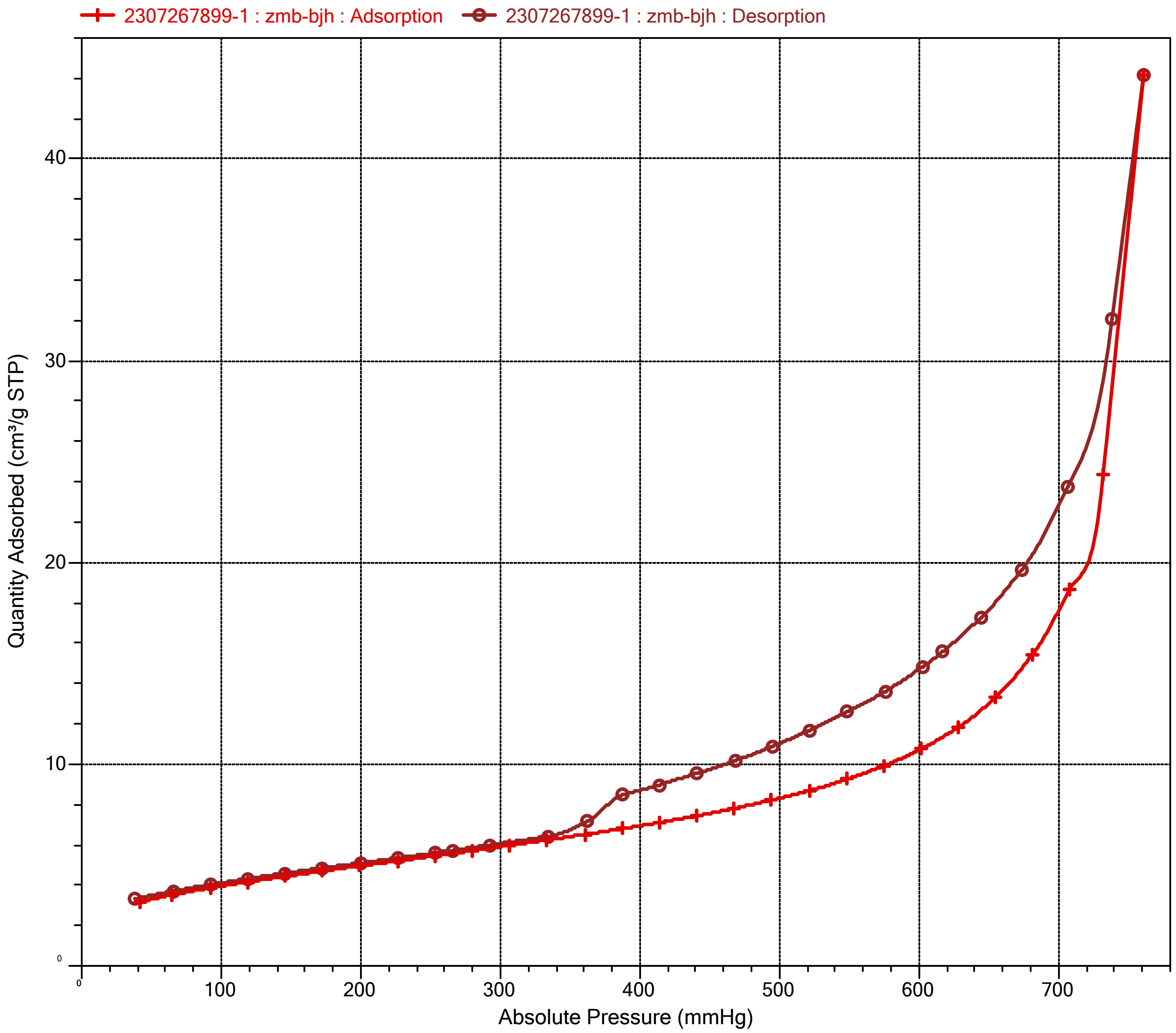

Sample: zmb-bjh
Operator:
Submitter:
File: E:\ASAP 2460\data\2307267899-1.SMP

Started:	2023/7/30 8:14:01	Analysis adsorptive:	N2
Completed:	2023/7/30 11:44:14	Analysis bath temp.:	-195.850 °C
Report time:	2023/7/30 19:22:42	Thermal correction:	No
Sample mass:	0.1315 g	Ambient free space:	17.2326 cm³ Measured
Analysis free space:	48.6379 cm³	Equilibration interval:	6 s
Low pressure dose:	None	Sample density:	1.000 g/cm³
Automatic degas:	No		

Isotherm Log Absolute Plot

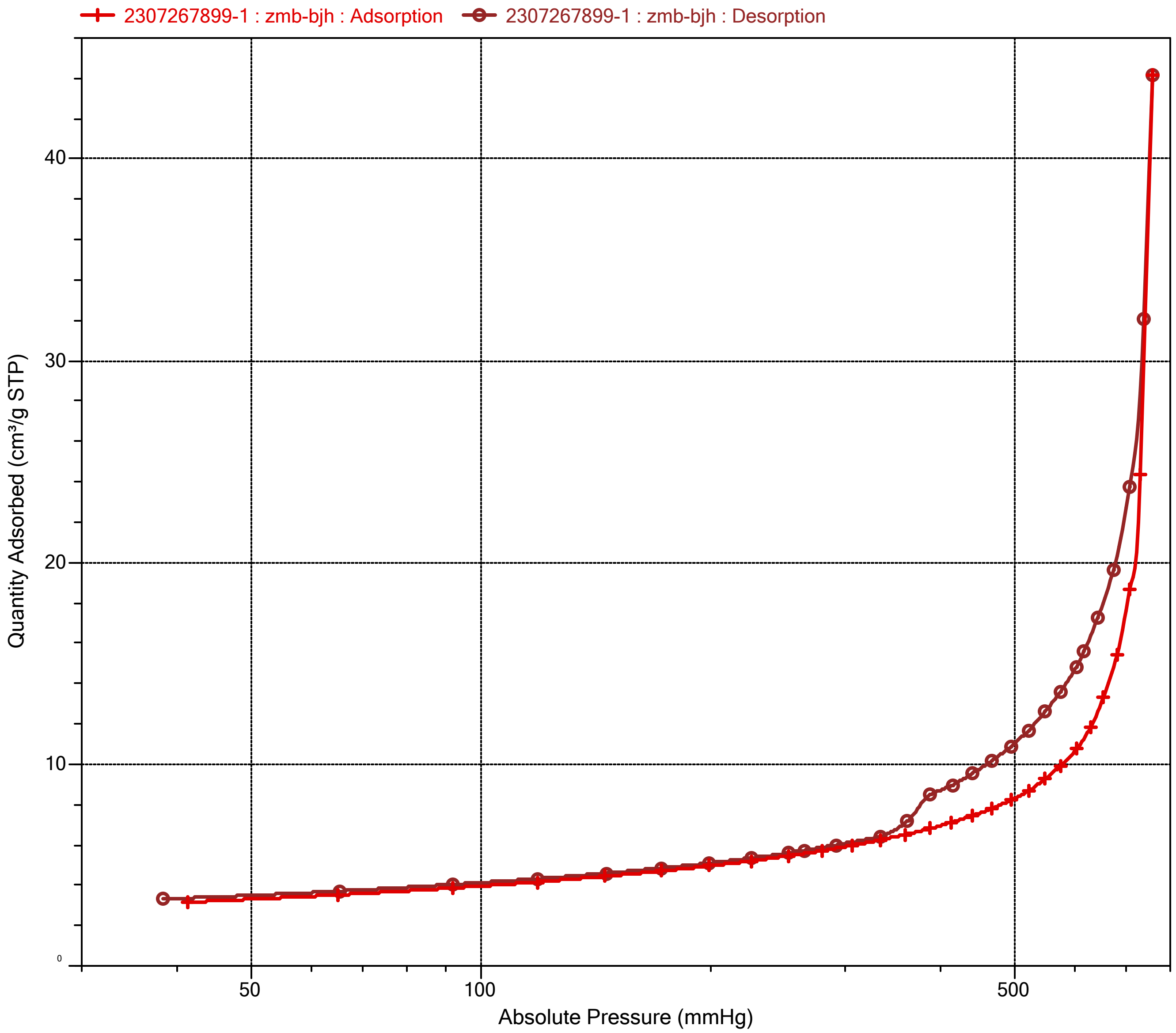

Sample:	zmb-bjh
Operator:	
Submitter:	
File:	E:\ASAP 2460\data\2307267899-1.SMP

Started:	2023/7/30 8:14:01	Analysis adsorptive:	N2
Completed:	2023/7/30 11:44:14	Analysis bath temp.:	-195.850 °C
Report time:	2023/7/30 19:22:42	Thermal correction:	No
Sample mass:	0.1315 g	Ambient free space:	17.2326 cm³ Measured
Analysis free space:	48.6379 cm³	Equilibration interval:	6 s
Low pressure dose:	None	Sample density:	1.000 g/cm³
Automatic degas:	No		

Isotherm Pressure Composition

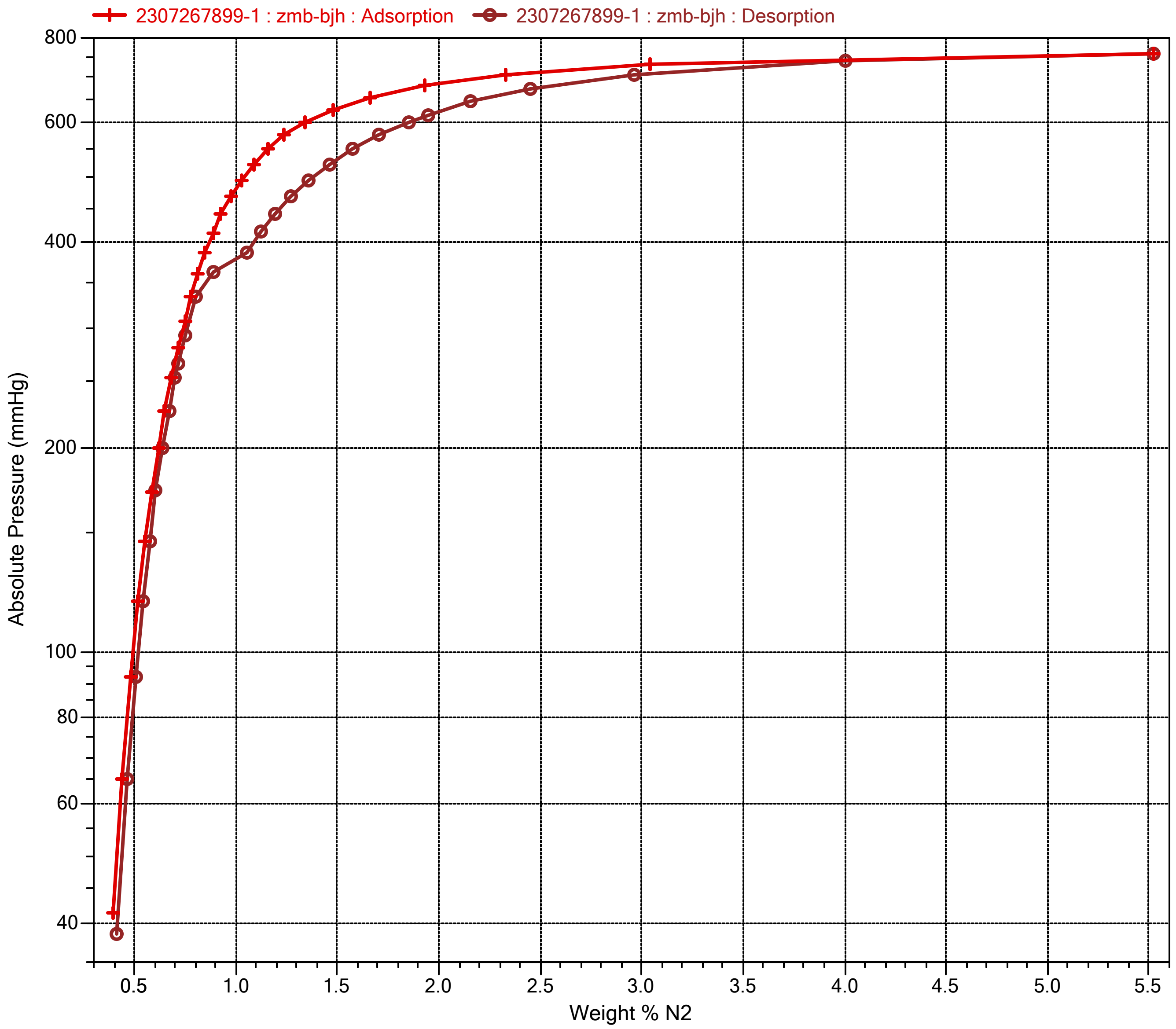

Sample: zmb-bjh
Operator:
Submitter:
File: E:\ASAP 2460\data\2307267899-1.SMP

Started:	2023/7/30 8:14:01	Analysis adsorptive:	N2
Completed:	2023/7/30 11:44:14	Analysis bath temp.:	-195.850 °C
Report time:	2023/7/30 19:22:42	Thermal correction:	No
Sample mass:	0.1315 g	Ambient free space:	17.2326 cm³ Measured
Analysis free space:	48.6379 cm³	Equilibration interval:	6 s
Low pressure dose:	None	Sample density:	1.000 g/cm³
Automatic degas:	No		

BET Report

BET surface area: 16.7594 ± 0.0652 m²/g
Slope: 0.255200 ± 0.000996 g/cm³ STP
Y-intercept: 0.004510 ± 0.000170 g/cm³ STP
C: 57.586408
Qm: 3.8505 cm³/g STP
Correlation coefficient: 0.9999619
Molecular cross-sectional area: 0.1620 nm²

Relative Pressure (P/Po)	Quantity Adsorbed (cm³/g STP)	1/[Q(Po/P - 1)]
0.053930052	3.1600	0.018039
0.084758177	3.5196	0.026312
0.120039808	3.8597	0.035343
0.154915522	4.1585	0.044082
0.189956615	4.4369	0.052853
0.224870737	4.6965	0.061771
0.259784074	4.9506	0.070891

Sample: zmb-bjh
Operator:
Submitter:
File: E:\ASAP 2460\data\2307267899-1.SMP

Started:	2023/7/30 8:14:01	Analysis adsorptive:	N2
Completed:	2023/7/30 11:44:14	Analysis bath temp.:	-195.850 °C
Report time:	2023/7/30 19:22:42	Thermal correction:	No
Sample mass:	0.1315 g	Ambient free space:	17.2326 cm³ Measured
Analysis free space:	48.6379 cm³	Equilibration interval:	6 s
Low pressure dose:	None	Sample density:	1.000 g/cm³
Automatic degas:	No		

BET Surface Area Plot

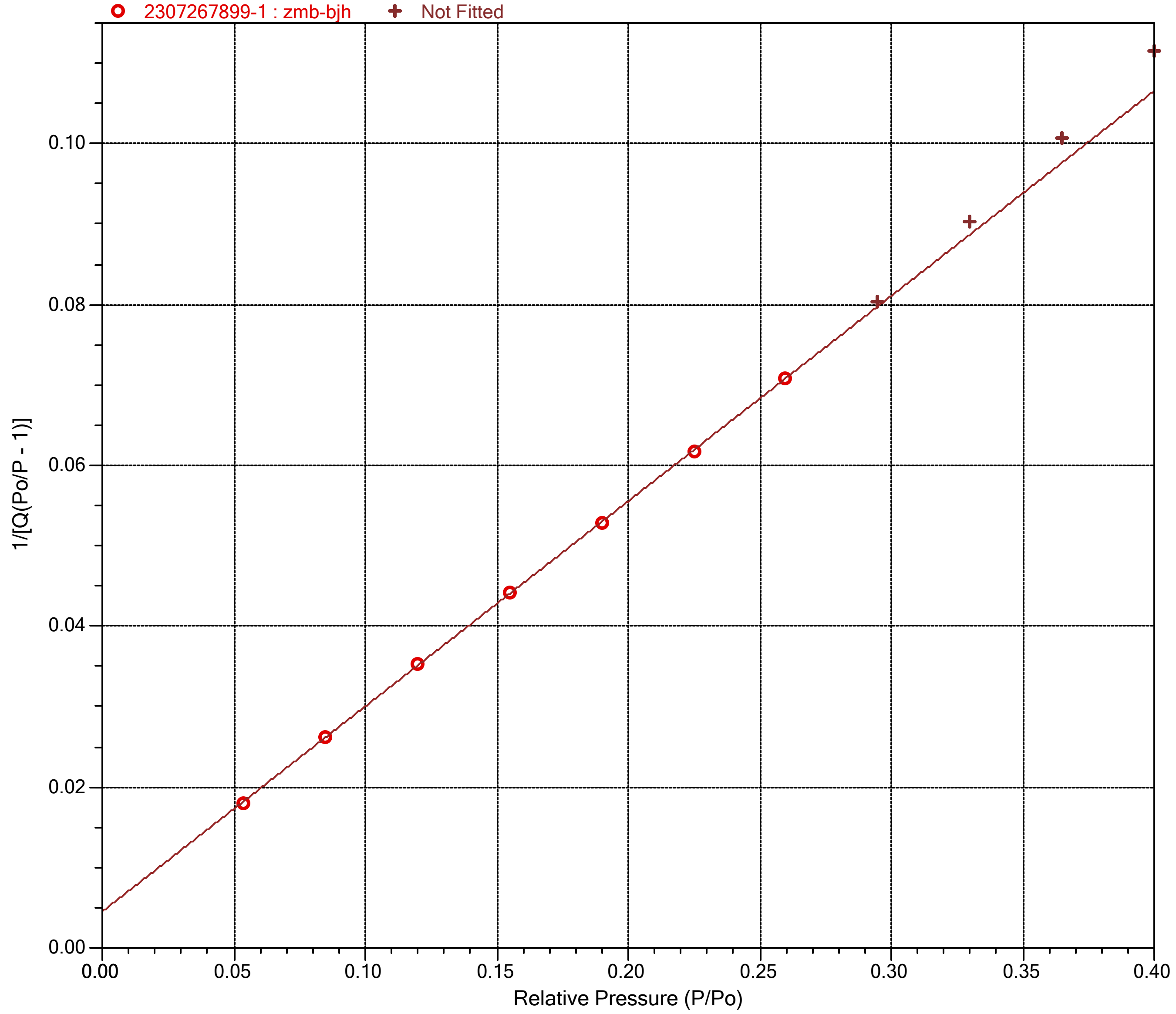

Sample: zmb-bjh
Operator:
Submitter:
File: E:\ASAP 2460\data\2307267899-1.SMP

Started: 2023/7/30 8:14:01
Completed: 2023/7/30 11:44:14
Report time: 2023/7/30 19:22:42
Sample mass: 0.1315 g
Analysis free space: 48.6379 cm³
Low pressure dose: None
Automatic degas: No

Analysis adsorptive: N2
Analysis bath temp.: -195.850 °C
Thermal correction: No
Ambient free space: 17.2326 cm³ Measured
Equilibration interval: 6 s
Sample density: 1.000 g/cm³

BJH Adsorption Pore Distribution Report

Faas Correction
Harkins and Jura
t = [13.99 / (0.034 - log(P/Po))] ^ 0.5

Diameter range: 3.7602 to 300.0000 nm
Adsorbate property factor: 0.95300 nm
Density conversion factor: 0.0015468
Fraction of pores open at both ends: 0.00

Pore Diameter Range (nm)	Average Diameter (nm)	Incremental Pore Volume (cm³/g)	Cumulative Pore Volume (cm³/g)	Incremental Pore Area (m²/g)	Cumulative Pore Area (m²/g)
224.9 - 44.0	50.0	0.035184	0.035184	2.812	2.812
44.0 - 26.6	31.0	0.010120	0.045304	1.305	4.117
26.6 - 18.7	21.2	0.005765	0.051069	1.086	5.204
18.7 - 14.5	16.0	0.003823	0.054892	0.954	6.157
14.5 - 11.8	12.8	0.002567	0.057460	0.801	6.958
11.8 - 9.9	10.7	0.001811	0.059270	0.678	7.636
9.9 - 8.6	9.1	0.001352	0.060622	0.593	8.229
8.6 - 7.5	7.9	0.001045	0.061667	0.526	8.755
7.5 - 6.7	7.0	0.000855	0.062522	0.487	9.241
6.7 - 6.0	6.3	0.000662	0.063185	0.422	9.663
6.0 - 5.4	5.7	0.000571	0.063756	0.403	10.066
5.4 - 4.9	5.2	0.000494	0.064249	0.384	10.450
4.9 - 4.5	4.7	0.000390	0.064640	0.332	10.782
4.5 - 4.2	4.3	0.000393	0.065033	0.364	11.146
4.2 - 3.8	4.0	0.000339	0.065372	0.341	11.487

Sample: zmb-bjh
Operator:
Submitter:
File: E:\ASAP 2460\data\2307267899-1.SMP

Started: 2023/7/30 8:14:01
Completed: 2023/7/30 11:44:14
Report time: 2023/7/30 19:22:42
Sample mass: 0.1315 g
Analysis free space: 48.6379 cm³
Low pressure dose: None
Automatic degas: No

Analysis adsorptive: N2
Analysis bath temp.: -195.850 °C
Thermal correction: No
Ambient free space: 17.2326 cm³ Measured
Equilibration interval: 6 s
Sample density: 1.000 g/cm³

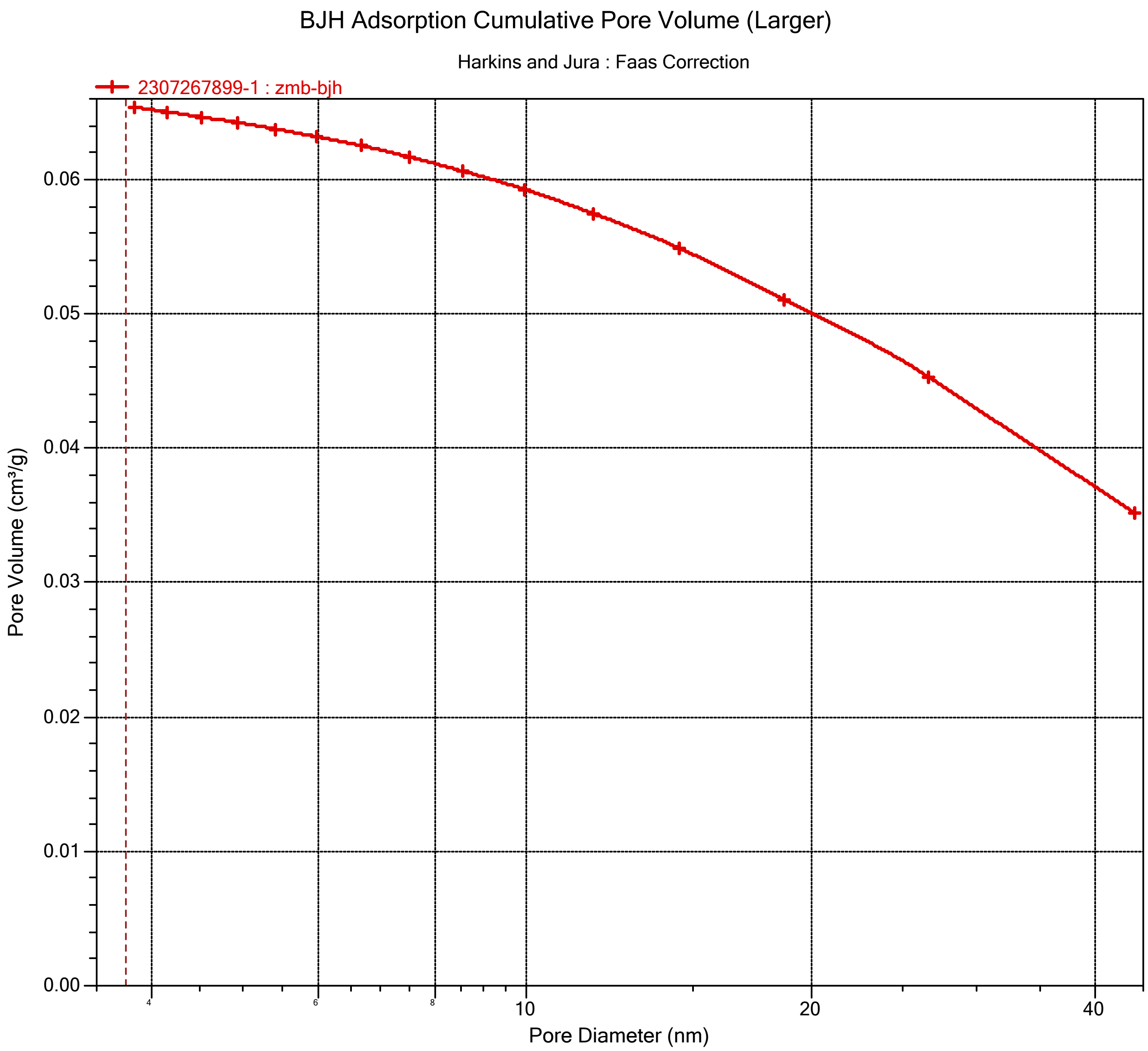

Sample: zmb-bjh
Operator:
Submitter:
File: E:\ASAP 2460\data\2307267899-1.SMP

Started:	2023/7/30 8:14:01	Analysis adsorptive:	N2
Completed:	2023/7/30 11:44:14	Analysis bath temp.:	-195.850 °C
Report time:	2023/7/30 19:22:42	Thermal correction:	No
Sample mass:	0.1315 g	Ambient free space:	17.2326 cm³ Measured
Analysis free space:	48.6379 cm³	Equilibration interval:	6 s
Low pressure dose:	None	Sample density:	1.000 g/cm³
Automatic degas:	No		

BJH Adsorption dV/dD Pore Volume

Harkins and Jura : Faas Correction

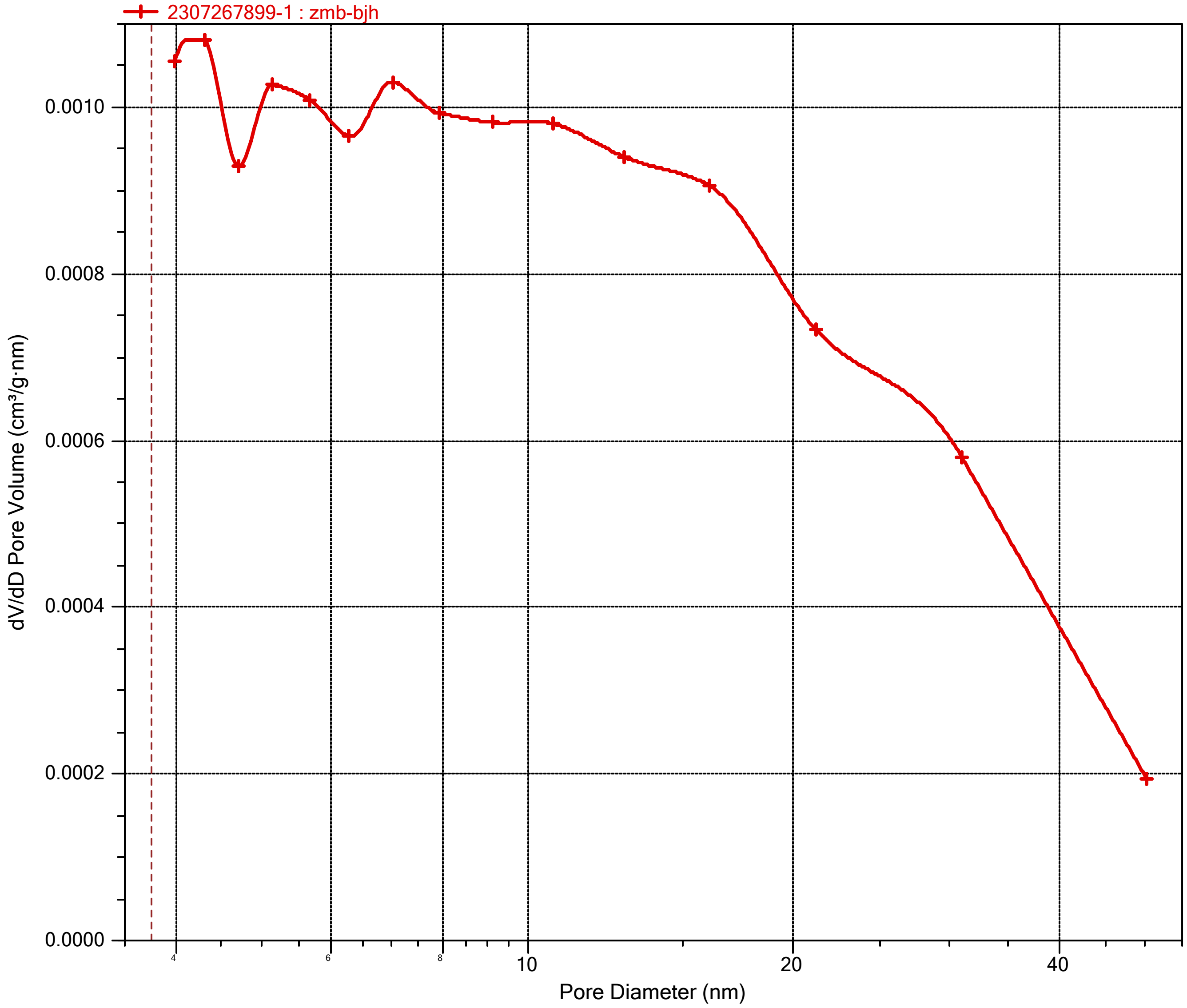

Sample: zmb-bjh
Operator:
Submitter:
File: E:\ASAP 2460\data\2307267899-1.SMP

Started:	2023/7/30 8:14:01	Analysis adsorptive:	N2
Completed:	2023/7/30 11:44:14	Analysis bath temp.:	-195.850 °C
Report time:	2023/7/30 19:22:42	Thermal correction:	No
Sample mass:	0.1315 g	Ambient free space:	17.2326 cm³ Measured
Analysis free space:	48.6379 cm³	Equilibration interval:	6 s
Low pressure dose:	None	Sample density:	1.000 g/cm³
Automatic degas:	No		

BJH Adsorption dV/dlog(D) Pore Volume

Harkins and Jura : Faas Correction

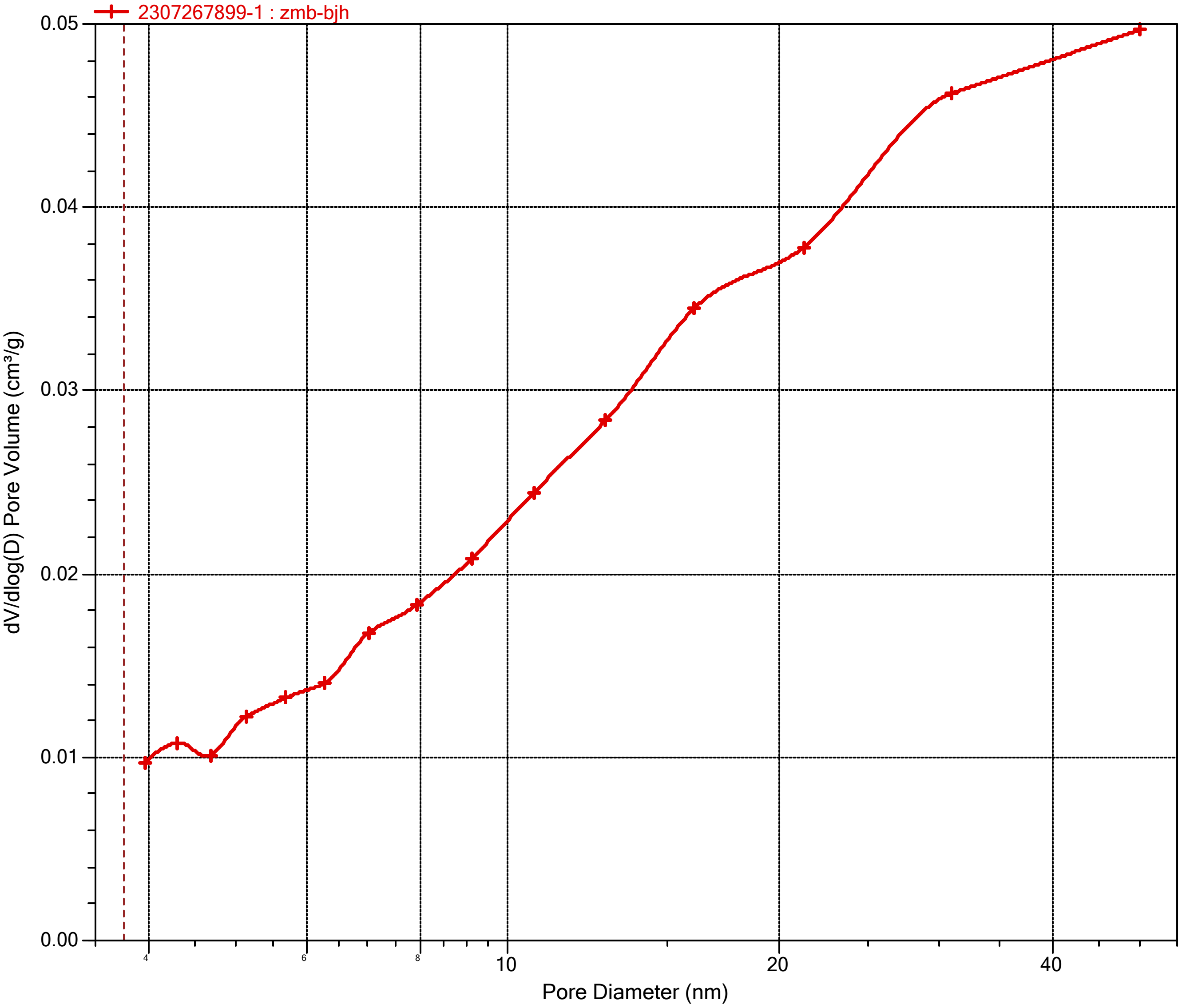

Sample: zmb-bjh
Operator:
Submitter:
File: E:\ASAP 2460\data\2307267899-1.SMP

Started:	2023/7/30 8:14:01	Analysis adsorptive:	N2
Completed:	2023/7/30 11:44:14	Analysis bath temp.:	-195.850 °C
Report time:	2023/7/30 19:22:42	Thermal correction:	No
Sample mass:	0.1315 g	Ambient free space:	17.2326 cm³ Measured
Analysis free space:	48.6379 cm³	Equilibration interval:	6 s
Low pressure dose:	None	Sample density:	1.000 g/cm³
Automatic degas:	No		

BJH Desorption Pore Distribution Report

Faas Correction
Harkins and Jura
t = [13.99 / (0.034 - log(P/Po))] ^ 0.5

Diameter range: 2.3123 to 21.7646 nm
Adsorbate property factor: 0.95300 nm
Density conversion factor: 0.0015468
Fraction of pores open at both ends: 0.00

Pore Diameter Range (nm)	Average Diameter (nm)	Incremental Pore Volume (cm³/g)	Cumulative Pore Volume (cm³/g)	Incremental Pore Area (m²/g)	Cumulative Pore Area (m²/g)
26.0 - 17.0	19.6	0.007525	0.007525	1.538	1.538
17.0 - 13.1	14.5	0.004347	0.011872	1.200	2.738
13.1 - 10.7	11.6	0.003164	0.015036	1.088	3.825
10.7 - 9.8	10.2	0.001337	0.016373	0.523	4.348
9.8 - 8.4	9.0	0.002240	0.018612	0.994	5.342
8.4 - 7.4	7.8	0.001895	0.020507	0.969	6.311
7.4 - 6.5	6.9	0.001621	0.022128	0.940	7.252
6.5 - 5.8	6.1	0.001445	0.023573	0.940	8.192
5.8 - 5.3	5.5	0.001249	0.024822	0.903	9.096
5.3 - 4.8	5.0	0.001088	0.025910	0.870	9.965
4.8 - 4.4	4.6	0.000948	0.026858	0.832	10.798
4.4 - 4.0	4.2	0.000838	0.027696	0.803	11.600
4.0 - 3.7	3.8	0.003431	0.031127	3.575	15.176
3.7 - 3.4	3.5	0.001476	0.032603	1.672	16.848
3.4 - 2.8	2.9	0.000002	0.032606	0.003	16.851
2.8 - 2.7	2.7	0.000050	0.032656	0.073	16.924
2.7 - 2.3	2.4	0.000017	0.032673	0.029	16.953

Sample: zmb-bjh
Operator:
Submitter:
File: E:\ASAP 2460\data\2307267899-1.SMP

Started:	2023/7/30 8:14:01	Analysis adsorptive:	N2
Completed:	2023/7/30 11:44:14	Analysis bath temp.:	-195.850 °C
Report time:	2023/7/30 19:22:42	Thermal correction:	No
Sample mass:	0.1315 g	Ambient free space:	17.2326 cm³ Measured
Analysis free space:	48.6379 cm³	Equilibration interval:	6 s
Low pressure dose:	None	Sample density:	1.000 g/cm³
Automatic degas:	No		

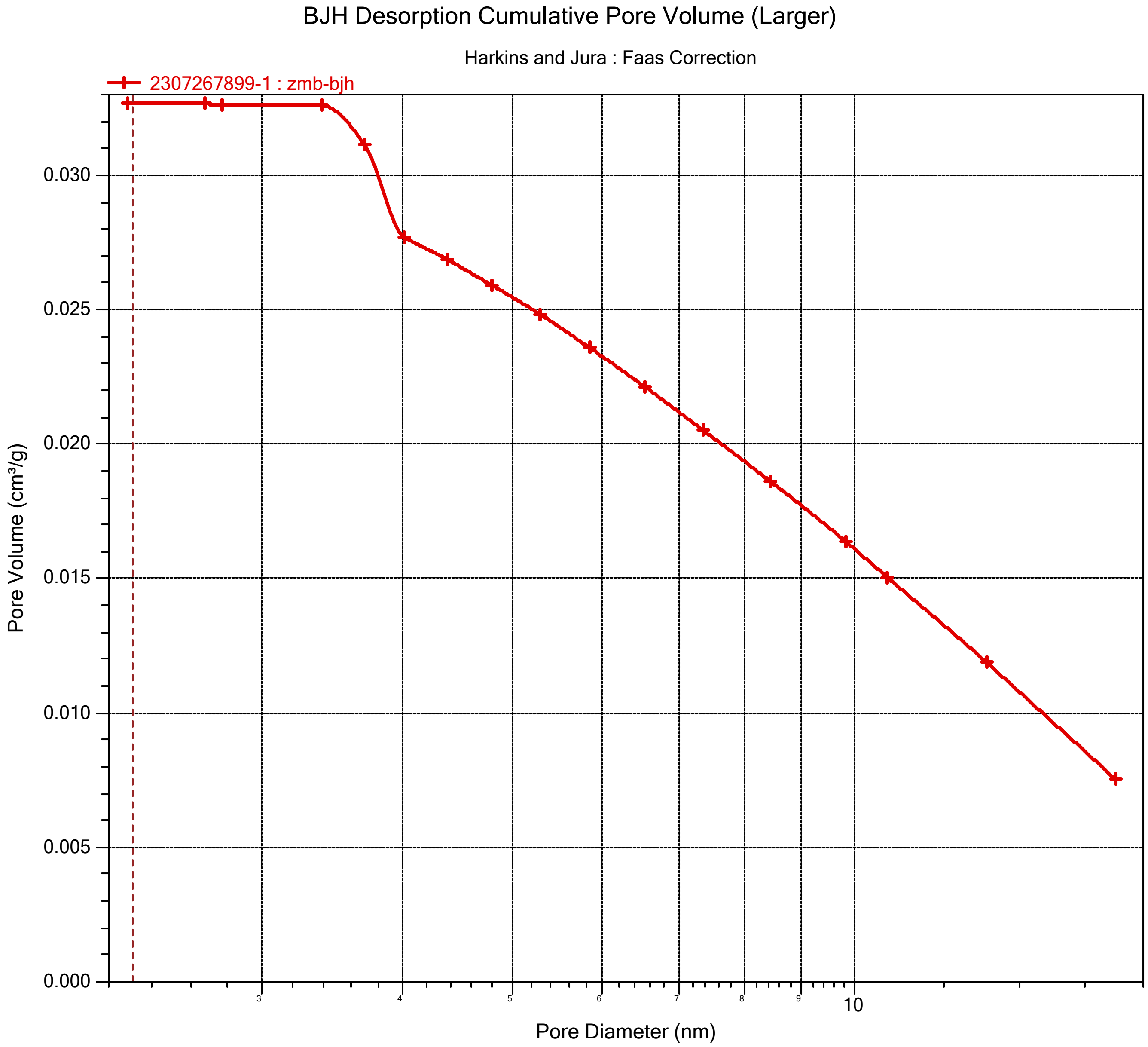

Sample: zmb-bjh
Operator:
Submitter:
File: E:\ASAP 2460\data\2307267899-1.SMP

Started:	2023/7/30 8:14:01	Analysis adsorptive:	N2
Completed:	2023/7/30 11:44:14	Analysis bath temp.:	-195.850 °C
Report time:	2023/7/30 19:22:42	Thermal correction:	No
Sample mass:	0.1315 g	Ambient free space:	17.2326 cm³ Measured
Analysis free space:	48.6379 cm³	Equilibration interval:	6 s
Low pressure dose:	None	Sample density:	1.000 g/cm³
Automatic degas:	No		

BJH Desorption dV/dD Pore Volume

Harkins and Jura : Faas Correction

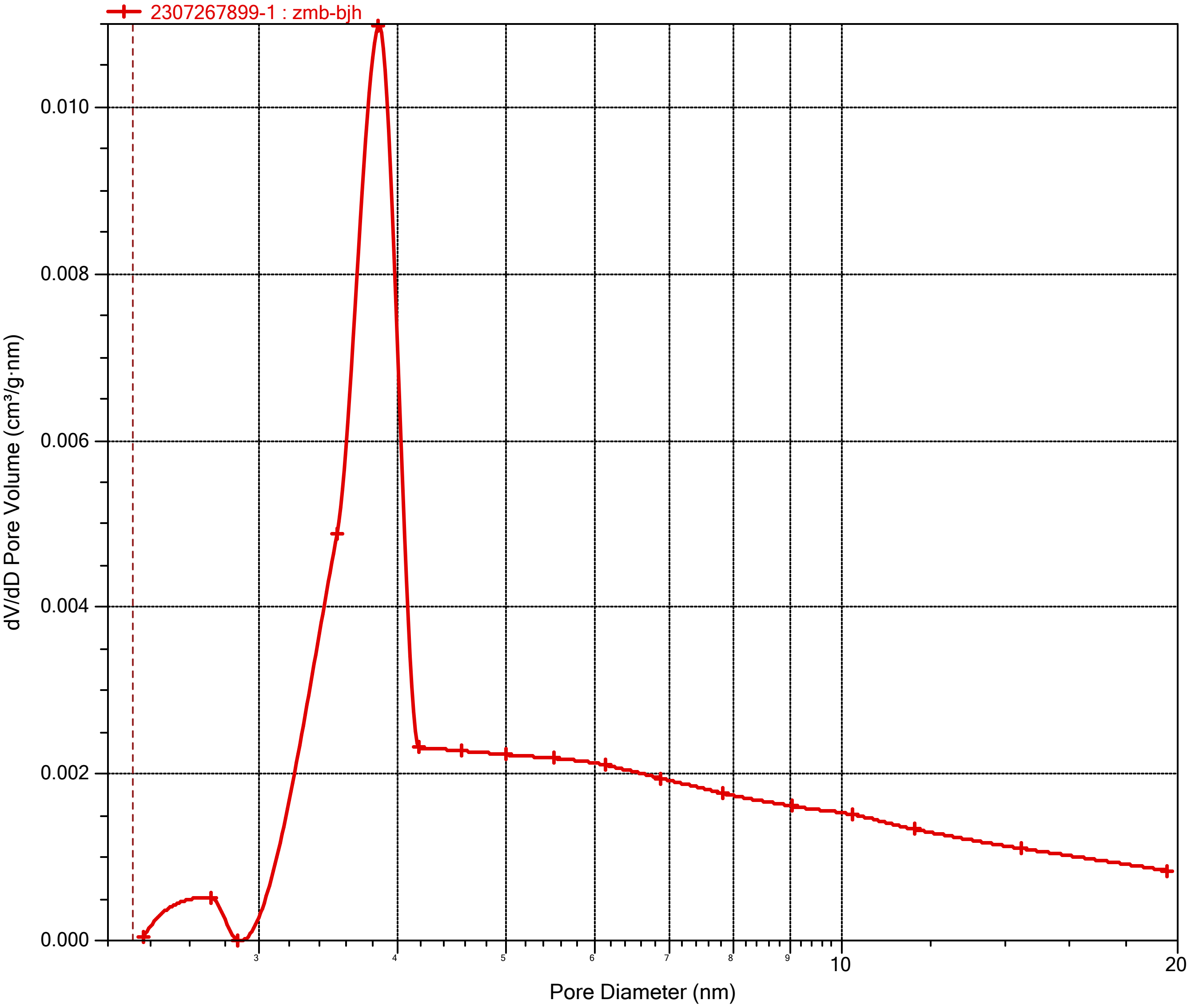

Sample: zmb-bjh
Operator:
Submitter:
File: E:\ASAP 2460\data\2307267899-1.SMP

Started:	2023/7/30 8:14:01	Analysis adsorptive:	N2
Completed:	2023/7/30 11:44:14	Analysis bath temp.:	-195.850 °C
Report time:	2023/7/30 19:22:42	Thermal correction:	No
Sample mass:	0.1315 g	Ambient free space:	17.2326 cm³ Measured
Analysis free space:	48.6379 cm³	Equilibration interval:	6 s
Low pressure dose:	None	Sample density:	1.000 g/cm³
Automatic degas:	No		

BJH Desorption dV/dlog(D) Pore Volume

Harkins and Jura : Faas Correction

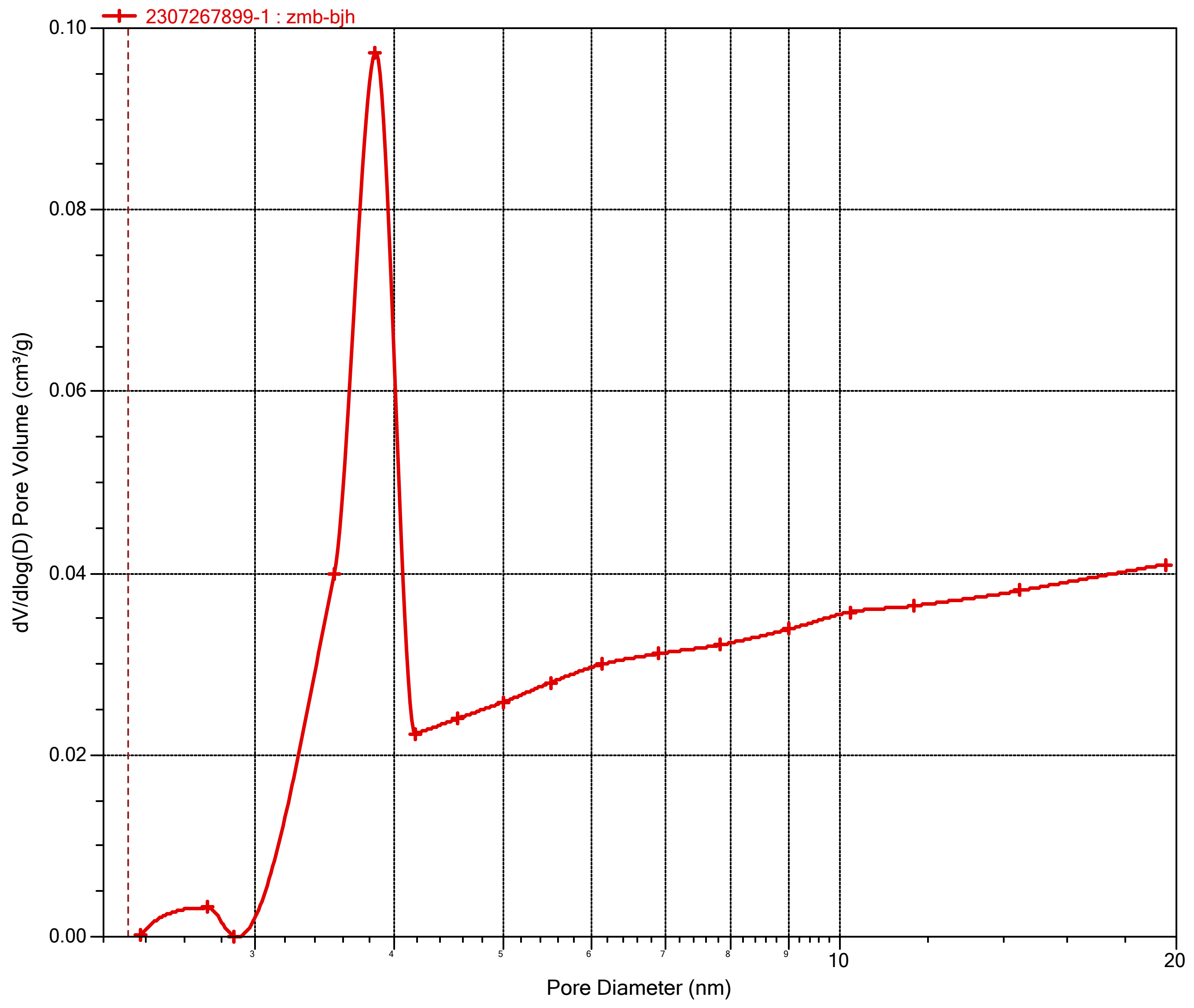

Sample: zmb-bjh
Operator:
Submitter:
File: E:\ASAP 2460\data\2307267899-1.SMP

Started:	2023/7/30 8:14:01	Analysis adsorptive:	N2
Completed:	2023/7/30 11:44:14	Analysis bath temp.:	-195.850 °C
Report time:	2023/7/30 19:22:42	Thermal correction:	No
Sample mass:	0.1315 g	Ambient free space:	17.2326 cm³ Measured
Analysis free space:	48.6379 cm³	Equilibration interval:	6 s
Low pressure dose:	None	Sample density:	1.000 g/cm³
Automatic degas:	No		

Porosity Distribution by Invalid
Model: N2 @ 77 on Carbon Slit Pores by NLDFT
Method: Non-negative Regularization: 0.00000
Standard Deviation of Fit: 0.46987 cm³/g STP

Volume in Pores	<	2.399 nm	0.00200 cm³/g
Total Volume in Pores	<=	99.993 nm	0.06517 cm³/g
Total Area in Pores	>=	2.399 nm	8.376 m²/g

Pore Table

Pore Width (nm)	Cumulative Pore Volume (cm³/g)	Incremental Pore Volume (cm³/g)	Cumulative Pore Area (m²/g)	Incremental Pore Area (m²/g)
2.399	0.00200	0.00000	0.000	0.000
2.514	0.00200	0.00000	0.000	0.000
2.628	0.00214	0.00014	0.105	0.105
2.742	0.00254	0.00040	0.393	0.288
2.885	0.00282	0.00029	0.591	0.198
2.999	0.00318	0.00036	0.834	0.242
3.141	0.00355	0.00037	1.069	0.235
3.284	0.00395	0.00040	1.312	0.243
3.427	0.00431	0.00035	1.517	0.206
3.598	0.00446	0.00015	1.602	0.084
3.769	0.00446	0.00000	1.602	0.000
3.912	0.00446	0.00000	1.602	0.000
4.112	0.00446	0.00000	1.602	0.000
4.283	0.00453	0.00007	1.637	0.035
4.483	0.00499	0.00046	1.841	0.204
4.682	0.00499	0.00000	1.841	0.000
4.882	0.00563	0.00064	2.102	0.261
5.110	0.00635	0.00073	2.387	0.285
5.339	0.00635	0.00000	2.387	0.000
5.595	0.00728	0.00092	2.717	0.330
5.852	0.00728	0.00000	2.717	0.000
6.109	0.00728	0.00000	2.717	0.000
6.366	0.00831	0.00103	3.041	0.324
6.680	0.00831	0.00000	3.041	0.000
6.965	0.00908	0.00077	3.262	0.221
7.279	0.00908	0.00000	3.262	0.000
7.622	0.00908	0.00000	3.262	0.000
7.964	0.00908	0.00000	3.262	0.000
8.306	0.01110	0.00203	3.750	0.488
8.677	0.01110	0.00000	3.750	0.000
9.077	0.01110	0.00000	3.750	0.000
9.476	0.01110	0.00000	3.750	0.000
9.904	0.01425	0.00315	4.385	0.635

Sample: zmb-bjh
Operator:
Submitter:
File: E:\ASAP 2460\data\2307267899-1.SMP

Started:	2023/7/30 8:14:01	Analysis adsorptive:	N2
Completed:	2023/7/30 11:44:14	Analysis bath temp.:	-195.850 °C
Report time:	2023/7/30 19:22:42	Thermal correction:	No
Sample mass:	0.1315 g	Ambient free space:	17.2326 cm³ Measured
Analysis free space:	48.6379 cm³	Equilibration interval:	6 s
Low pressure dose:	None	Sample density:	1.000 g/cm³
Automatic degas:	No		

Pore Table

Pore Width (nm)	Cumulative Pore Volume (cm³/g)	Incremental Pore Volume (cm³/g)	Cumulative Pore Area (m²/g)	Incremental Pore Area (m²/g)
10.361	0.01425	0.00000	4.385	0.000
10.818	0.01425	0.00000	4.385	0.000
11.303	0.01425	0.00000	4.385	0.000
11.816	0.01425	0.00000	4.385	0.000
12.359	0.01425	0.00000	4.385	0.000
12.929	0.01896	0.00471	5.113	0.728
13.500	0.01969	0.00073	5.222	0.109
14.099	0.02079	0.00109	5.377	0.155
14.727	0.02184	0.00105	5.520	0.143
15.412	0.02250	0.00066	5.605	0.085
16.097	0.02333	0.00084	5.709	0.104
16.810	0.02376	0.00043	5.760	0.051
17.581	0.02384	0.00008	5.770	0.010
18.380	0.02422	0.00038	5.811	0.042
19.207	0.02488	0.00066	5.880	0.069
20.063	0.02556	0.00067	5.947	0.067
20.976	0.02698	0.00142	6.082	0.135
21.918	0.02883	0.00185	6.251	0.169
22.888	0.03035	0.00152	6.384	0.133
23.944	0.03205	0.00171	6.526	0.142
25.000	0.03471	0.00266	6.739	0.213
26.141	0.03739	0.00268	6.944	0.205
27.311	0.03910	0.00171	7.069	0.125
28.538	0.04073	0.00163	7.183	0.114
29.823	0.04303	0.00229	7.337	0.154
31.164	0.04515	0.00213	7.474	0.137
32.562	0.04641	0.00126	7.551	0.077
34.046	0.04770	0.00129	7.627	0.076
35.558	0.04953	0.00183	7.730	0.103
37.185	0.05125	0.00172	7.822	0.092
38.840	0.05226	0.00101	7.874	0.052
40.609	0.05329	0.00103	7.925	0.051
42.435	0.05473	0.00144	7.993	0.068
44.319	0.05607	0.00133	8.053	0.060
46.316	0.05693	0.00086	8.090	0.037
48.399	0.05771	0.00079	8.123	0.033
50.597	0.05860	0.00088	8.158	0.035
52.880	0.05933	0.00073	8.185	0.028
55.248	0.06000	0.00068	8.210	0.024
57.731	0.06065	0.00065	8.232	0.023
60.328	0.06107	0.00041	8.246	0.014
63.038	0.06517	0.00411	8.376	0.130

Sample: zmb-bjh
Operator:
Submitter:
File: E:\ASAP 2460\data\2307267899-1.SMP

Started: 2023/7/30 8:14:01
Completed: 2023/7/30 11:44:14
Report time: 2023/7/30 19:22:42
Sample mass: 0.1315 g
Analysis free space: 48.6379 cm³
Low pressure dose: None
Automatic degas: No

Analysis adsorptive: N2
Analysis bath temp.: -195.850 °C
Thermal correction: No
Ambient free space: 17.2326 cm³ Measured
Equilibration interval: 6 s
Sample density: 1.000 g/cm³

Pore Table

Pore Width (nm)	Cumulative Pore Volume (cm³/g)	Incremental Pore Volume (cm³/g)	Cumulative Pore Area (m²/g)	Incremental Pore Area (m²/g)
65.892	0.06517	0.00000	8.376	0.000
68.860	0.06517	0.00000	8.376	0.000
71.942	0.06517	0.00000	8.376	0.000
75.195	0.06517	0.00000	8.376	0.000
78.591	0.06517	0.00000	8.376	0.000
82.101	0.06517	0.00000	8.376	0.000
85.810	0.06517	0.00000	8.376	0.000
89.663	0.06517	0.00000	8.376	0.000
93.715	0.06517	0.00000	8.376	0.000
97.938	0.06517	0.00000	8.376	0.000
99.993	0.06517	0.00000	8.376	0.000

Sample: zmb-bjh
Operator:
Submitter:
File: E:\ASAP 2460\data\2307267899-1.SMP

Started:	2023/7/30 8:14:01	Analysis adsorptive:	N2
Completed:	2023/7/30 11:44:14	Analysis bath temp.:	-195.850 °C
Report time:	2023/7/30 19:22:42	Thermal correction:	No
Sample mass:	0.1315 g	Ambient free space:	17.2326 cm³ Measured
Analysis free space:	48.6379 cm³	Equilibration interval:	6 s
Low pressure dose:	None	Sample density:	1.000 g/cm³
Automatic degas:	No		

Porosity Distribution by Invalid
Model: N2 @ 77 on Carbon Slit Pores by NLDFT
Method: Non-negative Regularization: 0.00000
Standard Deviation of Fit: 0.46987 cm³/g STP

Isotherm Table

Relative Pressure (P/Po)	Experimental Quantity Adsorbed (cm³/g STP)	Fitted Quantity Adsorbed (cm³/g STP)	Absolute Residual (cm³/g STP)	Relative Residual
0.056598000	3.1937	3.2404	-0.0468	-0.014643
0.070747500	3.3652	3.3599	0.0054	0.001595
0.088434400	3.5574	3.4343	0.1231	0.034598
0.110543000	3.7736	3.5464	0.2273	0.060226
0.116070000	3.8243	3.5811	0.2432	0.063589
0.121874000	3.8759	3.6227	0.2531	0.065312
0.127967000	3.9294	3.6750	0.2544	0.064737
0.134366000	3.9852	3.7457	0.2395	0.060089
0.141084000	4.0430	3.8557	0.1873	0.046329
0.148138000	4.1026	4.0564	0.0462	0.011251
0.155545000	4.1636	4.2760	-0.1124	-0.026992
0.163322000	4.2269	4.4135	-0.1866	-0.044143
0.171488000	4.2928	4.5118	-0.2190	-0.051020
0.180063000	4.3608	4.5909	-0.2302	-0.052782
0.189066000	4.4301	4.6587	-0.2285	-0.051581
0.198519000	4.5012	4.7190	-0.2177	-0.048375
0.208445000	4.5753	4.7742	-0.1988	-0.043458
0.218867000	4.6524	4.8258	-0.1733	-0.037256
0.229811000	4.7326	4.8746	-0.1420	-0.029999
0.241301000	4.8167	4.9216	-0.1050	-0.021799
0.253366000	4.9044	4.9674	-0.0630	-0.012852
0.266035000	4.9954	5.0125	-0.0171	-0.003424
0.279337000	5.0899	5.0987	-0.0088	-0.001724
0.293303000	5.1896	5.1436	0.0460	0.008871
0.307969000	5.2952	5.3141	-0.0189	-0.003565
0.323367000	5.4063	5.3602	0.0461	0.008525
0.339535000	5.5226	5.5010	0.0216	0.003906
0.356512000	5.6443	5.6713	-0.0270	-0.004790
0.374338000	5.7758	5.7233	0.0524	0.009079
0.393055000	5.9236	5.9063	0.0174	0.002934
0.412707000	6.0730	6.1071	-0.0341	-0.005610
0.433343000	6.2282	6.1751	0.0531	0.008519
0.455010000	6.3979	6.3812	0.0167	0.002616
0.477760000	6.5789	6.5386	0.0403	0.006126
0.501648000	6.7724	6.6953	0.0771	0.011387
0.526731000	6.9910	7.0141	-0.0230	-0.003294

Sample: zmb-bjh
Operator:
Submitter:
File: E:\ASAP 2460\data\2307267899-1.SMP

Started: 2023/7/30 8:14:01
Completed: 2023/7/30 11:44:14
Report time: 2023/7/30 19:22:42
Sample mass: 0.1315 g
Analysis free space: 48.6379 cm³
Low pressure dose: None
Automatic degas: No

Analysis adsorptive: N2
Analysis bath temp.: -195.850 °C
Thermal correction: No
Ambient free space: 17.2326 cm³ Measured
Equilibration interval: 6 s
Sample density: 1.000 g/cm³

Isotherm Table

Relative Pressure (P/Po)	Experimental Quantity Adsorbed (cm³/g STP)	Fitted Quantity Adsorbed (cm³/g STP)	Absolute Residual (cm³/g STP)	Relative Residual
0.553067000	7.2288	7.2775	-0.0487	-0.006737
0.580721000	7.4881	7.4757	0.0124	0.001654
0.609757000	7.8016	7.7818	0.0198	0.002534
0.640244000	8.1622	8.1530	0.0092	0.001128
0.672257000	8.5797	8.5636	0.0160	0.001865
0.705870000	9.1005	9.0822	0.0183	0.002016
0.741163000	9.7502	9.7397	0.0105	0.001079
0.778221000	10.5881	10.5712	0.0169	0.001592
0.817132000	11.7537	11.7413	0.0124	0.001055
0.857989000	13.4802	13.4672	0.0131	0.000969
0.899898000	16.3664	16.3647	0.0017	0.000106
0.903995000	16.7536	16.7519	0.0017	0.000102
0.907932000	17.1427	17.3161	-0.1733	-0.010112
0.911716000	17.5272	17.3519	0.1753	0.010000
0.915352000	17.9016	17.9002	0.0014	0.000078
0.918845000	18.2615	18.2601	0.0014	0.000074
0.922200000	18.6036	18.7167	-0.1130	-0.006075
0.925422000	18.8867	18.7720	0.1147	0.006073
0.928516000	19.0810	19.0799	0.0011	0.000059
0.931486000	19.2414	19.2403	0.0011	0.000057
0.934337000	19.4121	19.4960	-0.0840	-0.004325
0.937074000	19.6277	19.5425	0.0852	0.004343
0.939700000	19.9143	19.9134	0.0009	0.000047
0.942221000	20.2912	20.2903	0.0009	0.000044
0.944638000	20.7711	21.0499	-0.2788	-0.013421
0.946958000	21.3632	21.0830	0.2802	0.013114
0.949183000	22.0713	22.0705	0.0008	0.000034
0.951317000	22.8967	22.8960	0.0007	0.000031
0.953364000	23.8383	23.8377	0.0006	0.000027
0.955327000	24.8717	25.3426	-0.4709	-0.018934
0.957209000	25.8796	25.4057	0.4739	0.018313
0.959013000	26.8458	26.8453	0.0005	0.000017
0.960743000	27.7723	27.7719	0.0004	0.000016
0.962401000	28.6603	28.6599	0.0004	0.000015
0.963991000	29.5119	29.9067	-0.3948	-0.013378
0.965514000	30.3275	29.9314	0.3961	0.013061
0.966974000	31.1095	31.1092	0.0003	0.000010
0.968374000	31.8593	31.8590	0.0003	0.000010
0.969715000	32.5775	32.5772	0.0003	0.000009
0.971000000	33.2657	33.5845	-0.3188	-0.009583
0.972231000	33.9250	33.6051	0.3199	0.009429

Sample: zmb-bjh
Operator:
Submitter:
File: E:\ASAP 2460\data\2307267899-1.SMP

Started: 2023/7/30 8:14:01
Completed: 2023/7/30 11:44:14
Report time: 2023/7/30 19:22:42
Sample mass: 0.1315 g
Analysis free space: 48.6379 cm³
Low pressure dose: None
Automatic degas: No

Analysis adsorptive: N2
Analysis bath temp.: -195.850 °C
Thermal correction: No
Ambient free space: 17.2326 cm³ Measured
Equilibration interval: 6 s
Sample density: 1.000 g/cm³

Isotherm Table

Relative Pressure (P/Po)	Experimental Quantity Adsorbed (cm³/g STP)	Fitted Quantity Adsorbed (cm³/g STP)	Absolute Residual (cm³/g STP)	Relative Residual
0.973411000	34.5569	34.5567	0.0002	0.000006
0.974541000	35.1621	35.1619	0.0002	0.000006
0.975624000	35.7422	35.7420	0.0002	0.000005
0.976661000	36.2975	36.5466	-0.2490	-0.006861
0.977655000	36.8299	36.5791	0.2508	0.006810
0.978607000	37.3398	37.3397	0.0001	0.000003
0.979519000	37.8282	37.8281	0.0001	0.000002
0.980392000	38.2957	38.2957	0.0001	0.000002
0.981078510	38.6634	38.7918	-0.1283	-0.003319
0.981624530	38.9559	38.8261	0.1297	0.003331
0.982154930	39.2399	39.2399	0.0000	0.000000
0.982670160	39.5159	39.6309	-0.1151	-0.002912
0.983170640	39.7839	39.6673	0.1166	0.002931
0.983656790	40.0443	40.0443	-0.0001	-0.000002
0.984129010	40.2972	40.2972	-0.0001	-0.000002
0.984587690	40.5428	42.4495	-1.9066	-0.047028
0.985033210	40.7814	42.4510	-1.6696	-0.040940
0.985465960	41.0132	42.4529	-1.4397	-0.035103
0.985886280	41.2383	42.4544	-1.2161	-0.029488
0.986294530	41.4570	42.4558	-0.9988	-0.024094
0.986691050	41.6693	42.4572	-0.7879	-0.018908
0.987076170	41.8756	42.4586	-0.5830	-0.013922
0.987450220	42.0759	42.4602	-0.3843	-0.009133
0.987813510	42.2705	42.4615	-0.1910	-0.004518
0.988166350	42.4594	42.4627	-0.0033	-0.000077
0.988509030	42.6430	42.4639	0.1791	0.004199
0.988841840	42.8212	42.4651	0.3561	0.008317
0.989165070	42.9943	42.4662	0.5281	0.012283
0.989478980	43.1625	42.4673	0.6951	0.016105
0.989783850	43.3257	42.4687	0.8570	0.019781
0.990079930	43.4843	42.4698	1.0146	0.023331
0.990367470	43.6383	42.4708	1.1675	0.026755
0.990646710	43.7879	42.4717	1.3161	0.030057
0.990917900	43.9331	42.4727	1.4604	0.033242
0.991181250	44.0741	42.4736	1.6005	0.036315

Sample: zmb-bjh
Operator:
Submitter:
File: E:\ASAP 2460\data\2307267899-1.SMP

Started:	2023/7/30 8:14:01	Analysis adsorptive:	N2
Completed:	2023/7/30 11:44:14	Analysis bath temp.:	-195.850 °C
Report time:	2023/7/30 19:22:42	Thermal correction:	No
Sample mass:	0.1315 g	Ambient free space:	17.2326 cm³ Measured
Analysis free space:	48.6379 cm³	Equilibration interval:	6 s
Low pressure dose:	None	Sample density:	1.000 g/cm³
Automatic degas:	No		

Cumulative Surface Area vs. Pore Width

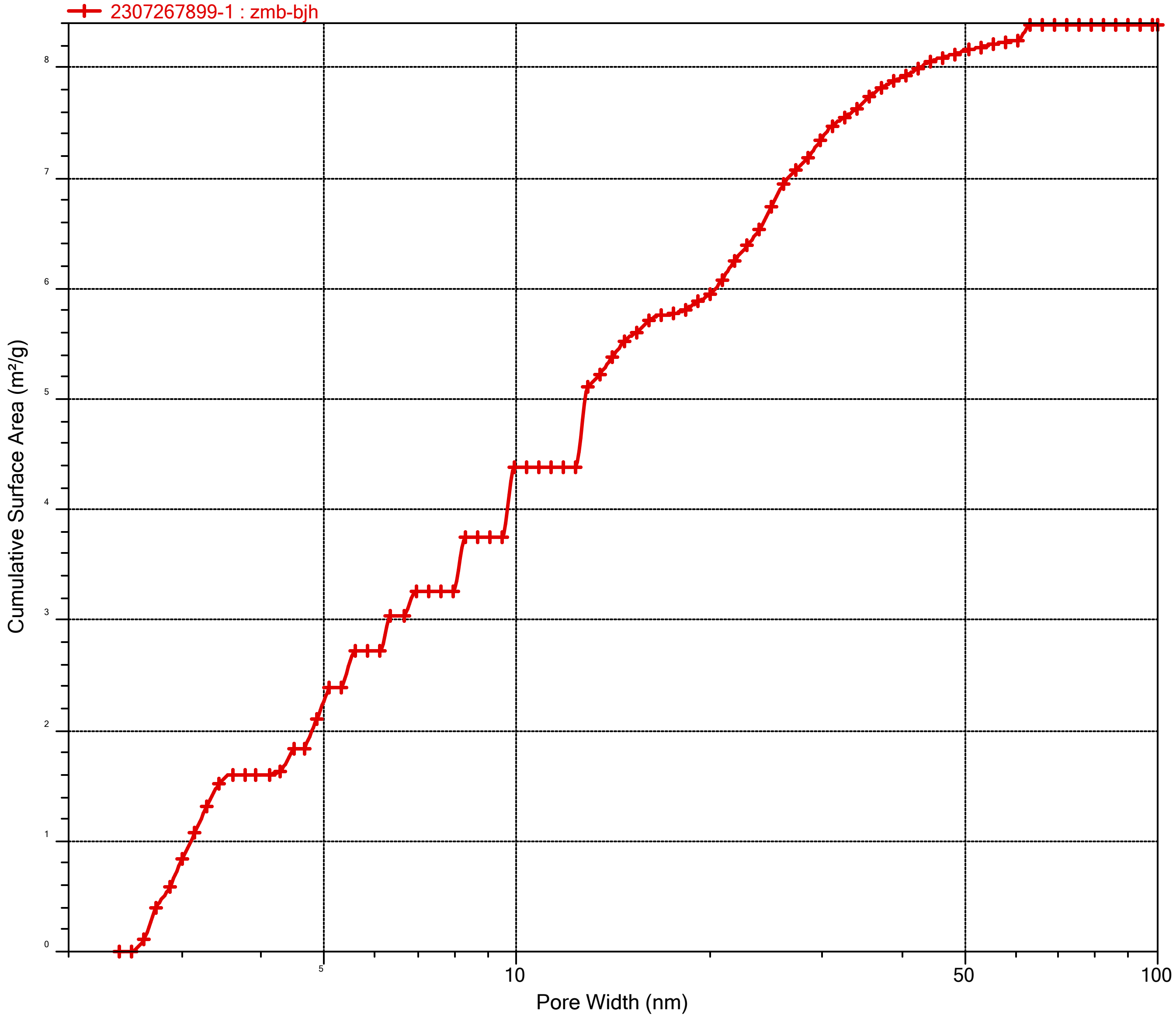

Sample: zmb-bjh
Operator:
Submitter:
File: E:\ASAP 2460\data\2307267899-1.SMP

Started:	2023/7/30 8:14:01	Analysis adsorptive:	N2
Completed:	2023/7/30 11:44:14	Analysis bath temp.:	-195.850 °C
Report time:	2023/7/30 19:22:42	Thermal correction:	No
Sample mass:	0.1315 g	Ambient free space:	17.2326 cm³ Measured
Analysis free space:	48.6379 cm³	Equilibration interval:	6 s
Low pressure dose:	None	Sample density:	1.000 g/cm³
Automatic degas:	No		

Incremental Surface Area vs. Pore Width

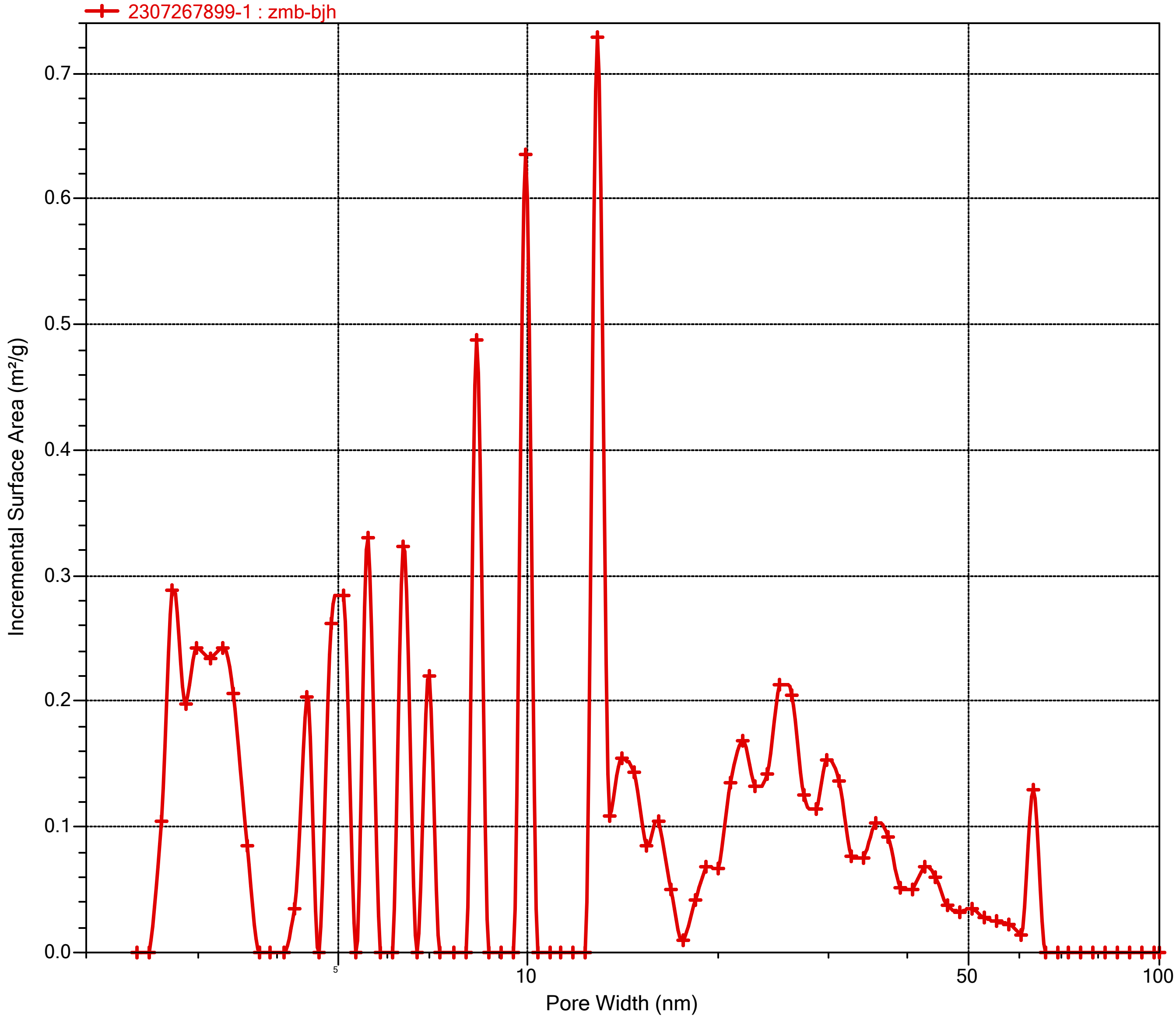

Sample: zmb-bjh
Operator:
Submitter:
File: E:\ASAP 2460\data\2307267899-1.SMP

Started:	2023/7/30 8:14:01	Analysis adsorptive:	N2
Completed:	2023/7/30 11:44:14	Analysis bath temp.:	-195.850 °C
Report time:	2023/7/30 19:22:42	Thermal correction:	No
Sample mass:	0.1315 g	Ambient free space:	17.2326 cm³ Measured
Analysis free space:	48.6379 cm³	Equilibration interval:	6 s
Low pressure dose:	None	Sample density:	1.000 g/cm³
Automatic degas:	No		

dA/dW Surface Area vs. Pore Width

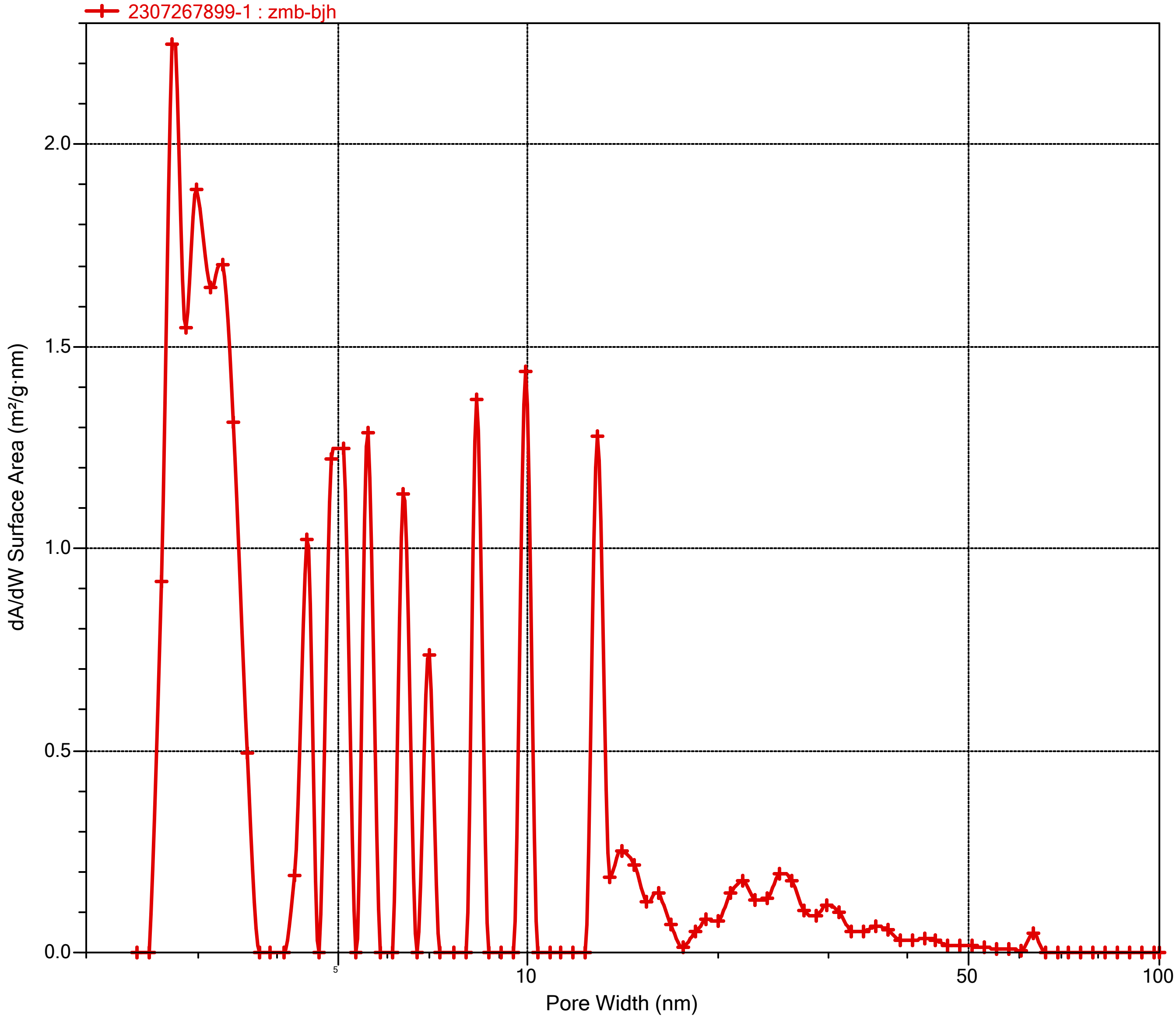

Sample: zmb-bjh
Operator:
Submitter:
File: E:\ASAP 2460\data\2307267899-1.SMP

Started: 2023/7/30 8:14:01
Completed: 2023/7/30 11:44:14
Report time: 2023/7/30 19:22:42
Sample mass: 0.1315 g
Analysis free space: 48.6379 cm³
Low pressure dose: None
Automatic degas: No

Analysis adsorptive: N2
Analysis bath temp.: -195.850 °C
Thermal correction: No
Ambient free space: 17.2326 cm³ Measured
Equilibration interval: 6 s
Sample density: 1.000 g/cm³

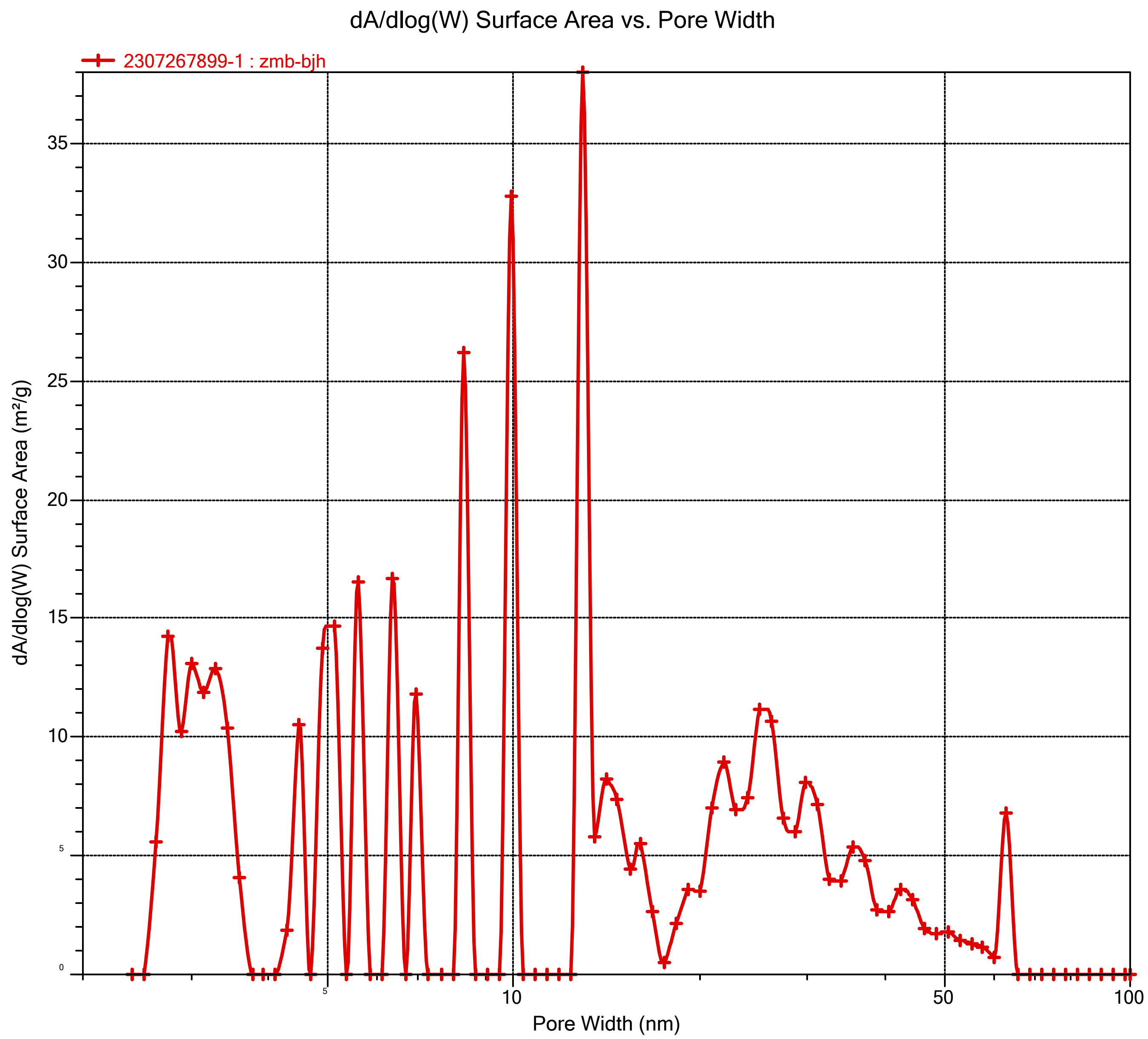

Sample:	zmb-bjh
Operator:	
Submitter:	
File:	E:\ASAP 2460\data\2307267899-1.SMP

Started:	2023/7/30 8:14:01	Analysis adsorptive:	N2
Completed:	2023/7/30 11:44:14	Analysis bath temp.:	-195.850 °C
Report time:	2023/7/30 19:22:42	Thermal correction:	No
Sample mass:	0.1315 g	Ambient free space:	17.2326 cm³ Measured
Analysis free space:	48.6379 cm³	Equilibration interval:	6 s
Low pressure dose:	None	Sample density:	1.000 g/cm³
Automatic degas:	No		

Cumulative Pore Volume vs. Pore Width

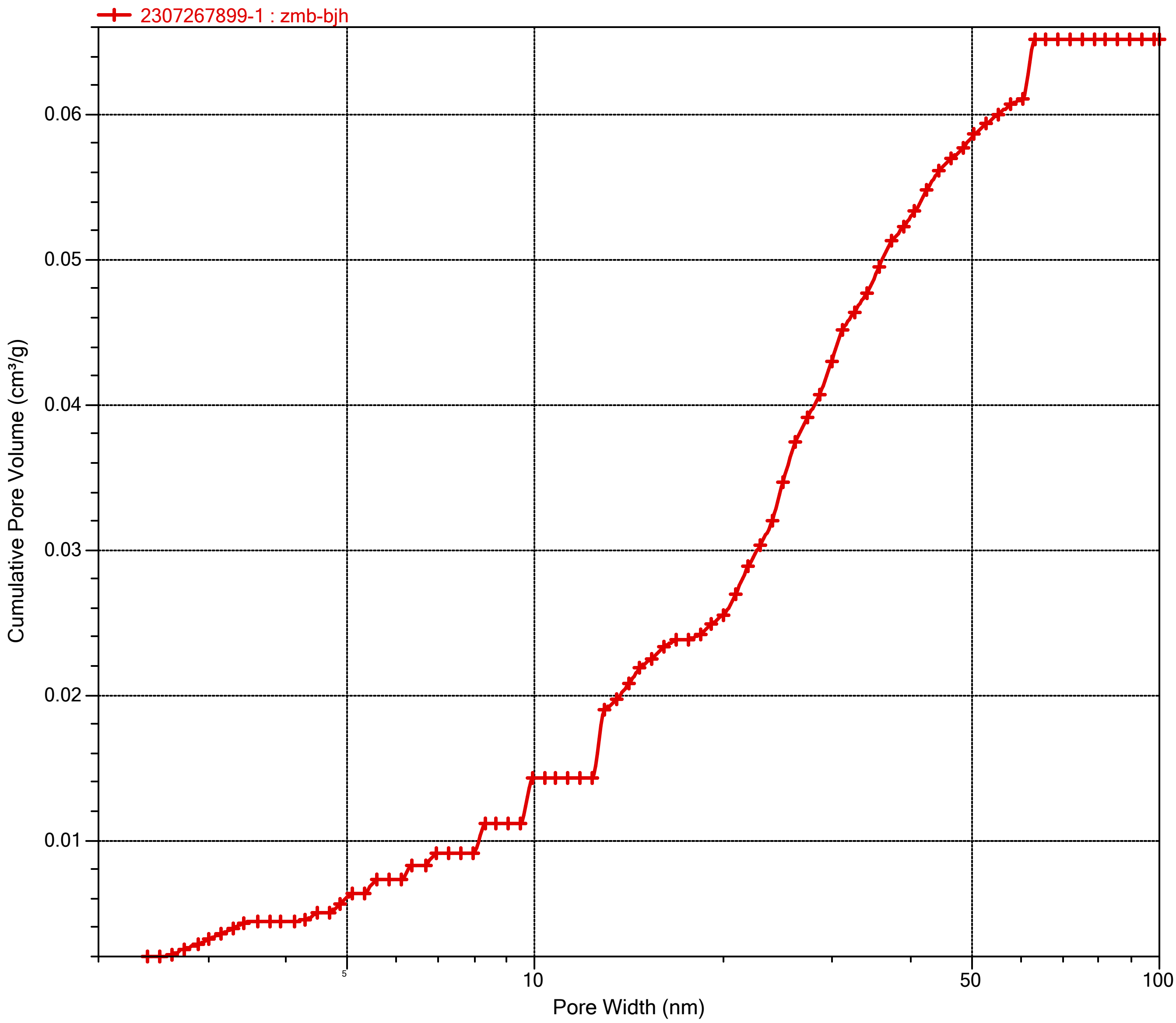

Sample: zmb-bjh
Operator:
Submitter:
File: E:\ASAP 2460\data\2307267899-1.SMP

Started:	2023/7/30 8:14:01	Analysis adsorptive:	N2
Completed:	2023/7/30 11:44:14	Analysis bath temp.:	-195.850 °C
Report time:	2023/7/30 19:22:42	Thermal correction:	No
Sample mass:	0.1315 g	Ambient free space:	17.2326 cm³ Measured
Analysis free space:	48.6379 cm³	Equilibration interval:	6 s
Low pressure dose:	None	Sample density:	1.000 g/cm³
Automatic degas:	No		

Incremental Pore Volume vs. Pore Width

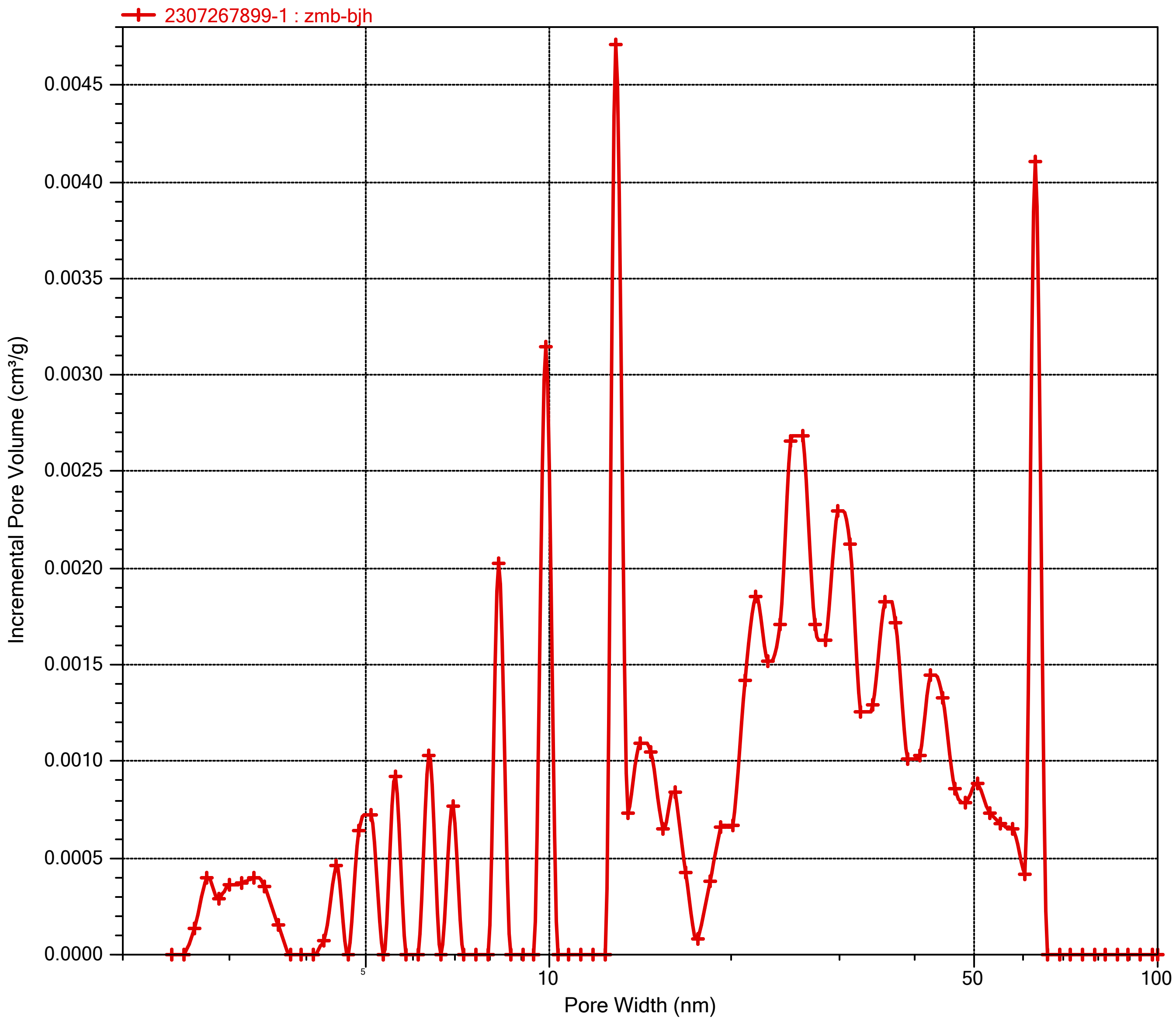

Sample: zmb-bjh
Operator:
Submitter:
File: E:\ASAP 2460\data\2307267899-1.SMP

Started: 2023/7/30 8:14:01
Completed: 2023/7/30 11:44:14
Report time: 2023/7/30 19:22:42
Sample mass: 0.1315 g
Analysis free space: 48.6379 cm³
Low pressure dose: None
Automatic degas: No

Analysis adsorptive: N2
Analysis bath temp.: -195.850 °C
Thermal correction: No
Ambient free space: 17.2326 cm³ Measured
Equilibration interval: 6 s
Sample density: 1.000 g/cm³

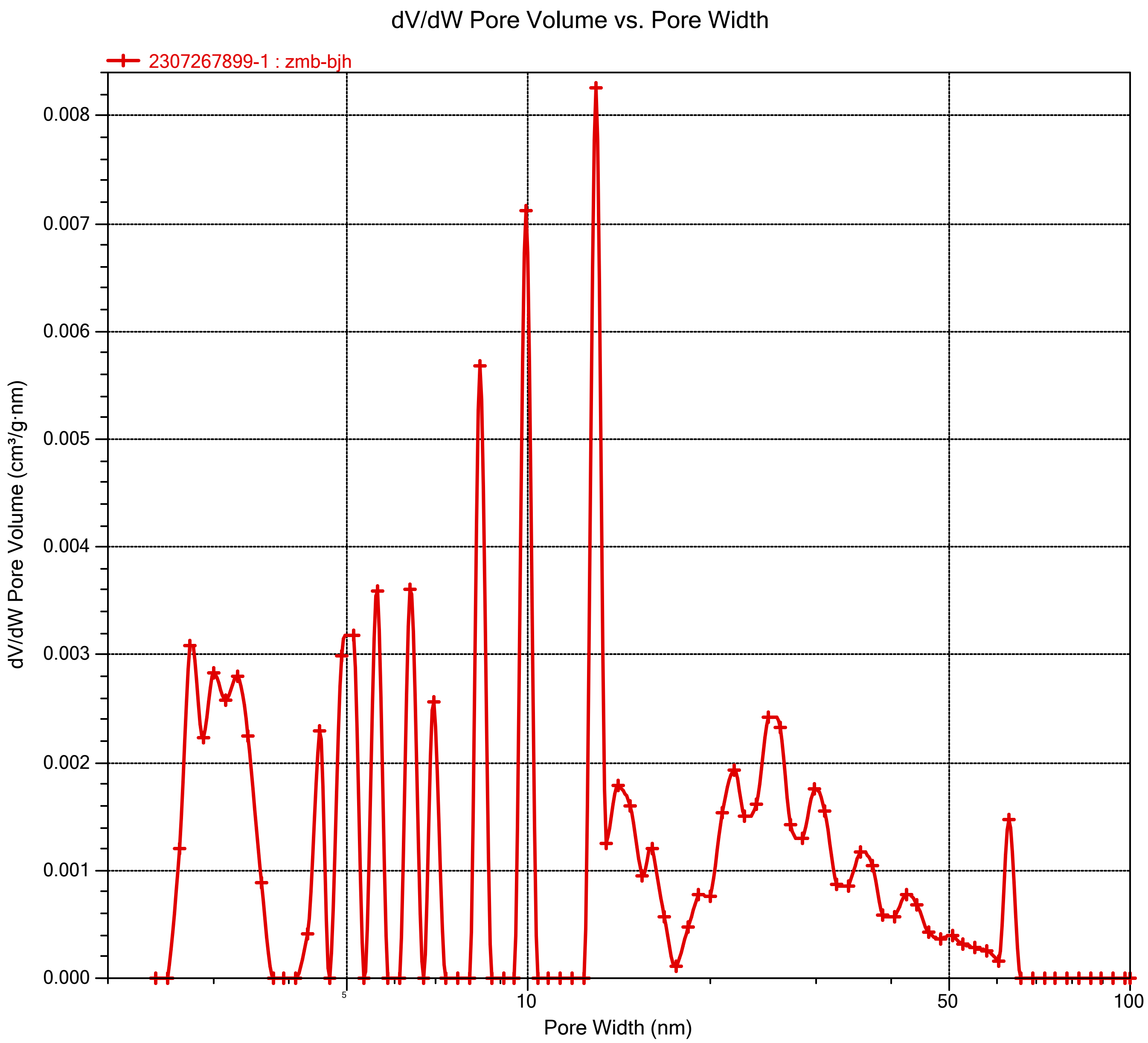

Sample: zmb-bjh
Operator:
Submitter:
File: E:\ASAP 2460\data\2307267899-1.SMP

Started:	2023/7/30 8:14:01	Analysis adsorptive:	N2
Completed:	2023/7/30 11:44:14	Analysis bath temp.:	-195.850 °C
Report time:	2023/7/30 19:22:42	Thermal correction:	No
Sample mass:	0.1315 g	Ambient free space:	17.2326 cm³ Measured
Analysis free space:	48.6379 cm³	Equilibration interval:	6 s
Low pressure dose:	None	Sample density:	1.000 g/cm³
Automatic degas:	No		

dV/dlog(W) Pore Volume vs. Pore Width

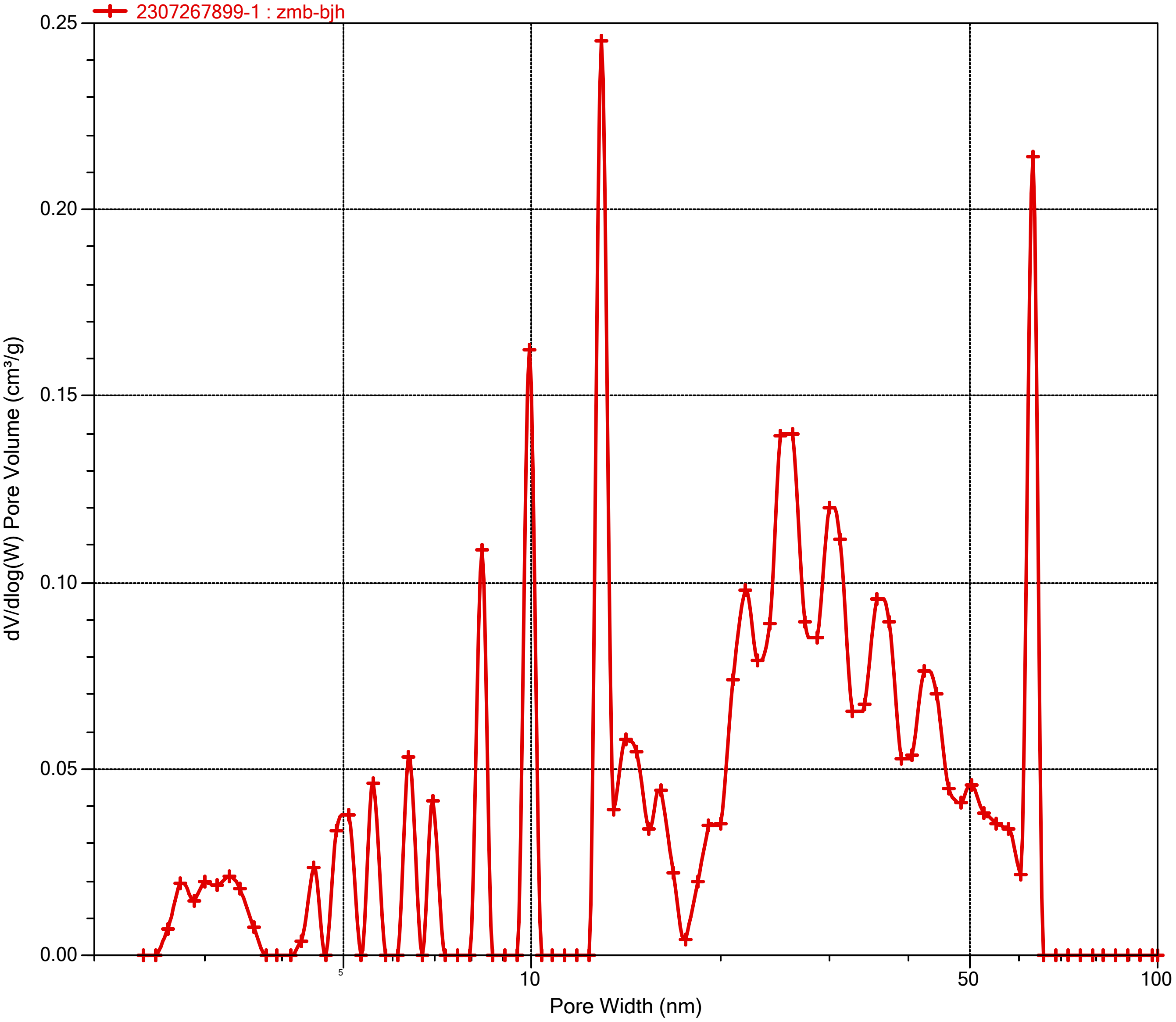

Sample: zmb-bjh
Operator:
Submitter:
File: E:\ASAP 2460\data\2307267899-1.SMP

Started:	2023/7/30 8:14:01	Analysis adsorptive:	N2
Completed:	2023/7/30 11:44:14	Analysis bath temp.:	-195.850 °C
Report time:	2023/7/30 19:22:42	Thermal correction:	No
Sample mass:	0.1315 g	Ambient free space:	17.2326 cm³ Measured
Analysis free space:	48.6379 cm³	Equilibration interval:	6 s
Low pressure dose:	None	Sample density:	1.000 g/cm³
Automatic degas:	No		

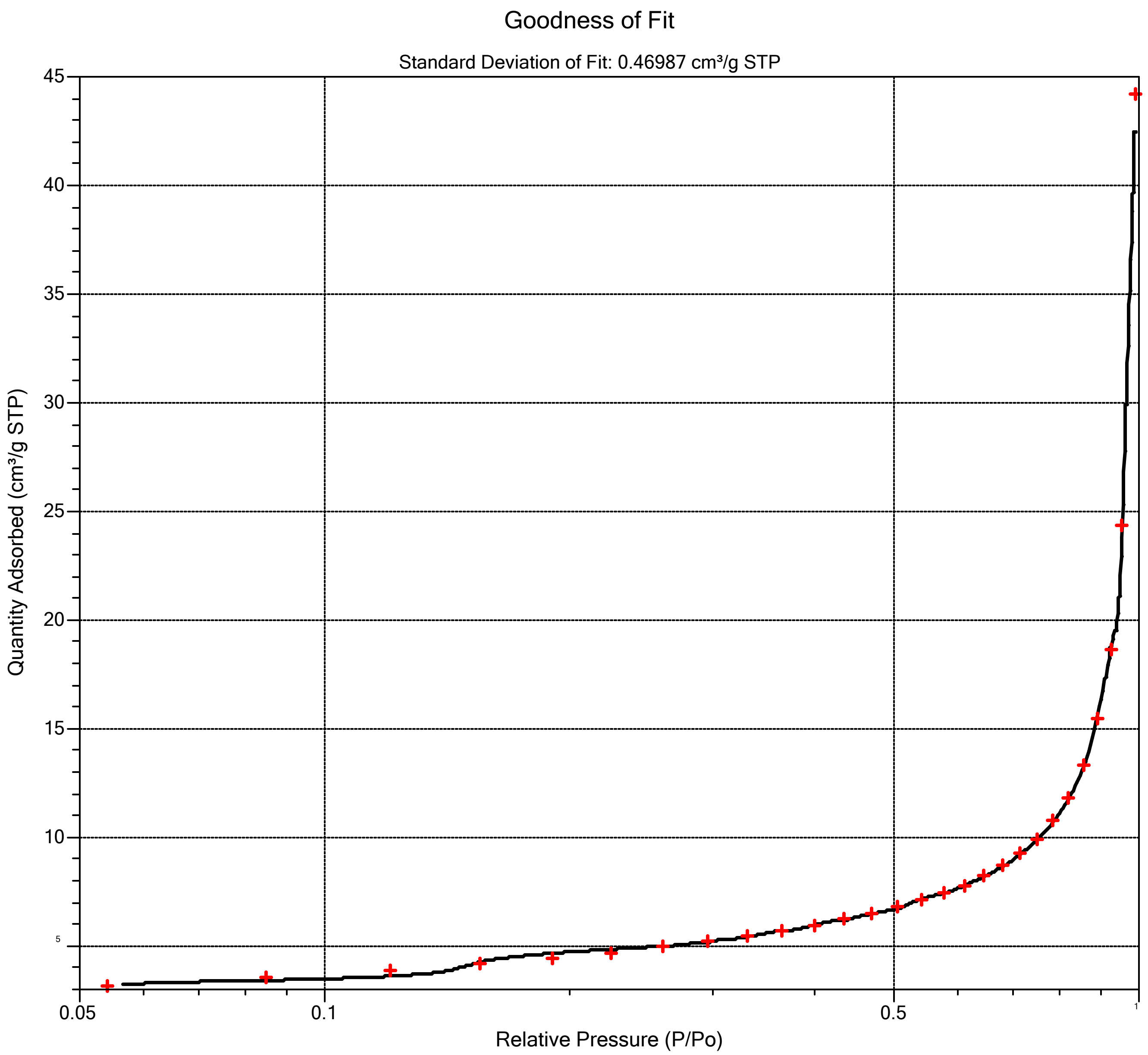

Sample: zmb-bjh
Operator:
Submitter:
File: E:\ASAP 2460\data\2307267899-1.SMP

Started:	2023/7/30 8:14:01	Analysis adsorptive:	N2
Completed:	2023/7/30 11:44:14	Analysis bath temp.:	-195.850 °C
Report time:	2023/7/30 19:22:42	Thermal correction:	No
Sample mass:	0.1315 g	Ambient free space:	17.2326 cm³ Measured
Analysis free space:	48.6379 cm³	Equilibration interval:	6 s
Low pressure dose:	None	Sample density:	1.000 g/cm³
Automatic degas:	No		

Goodness of Fit

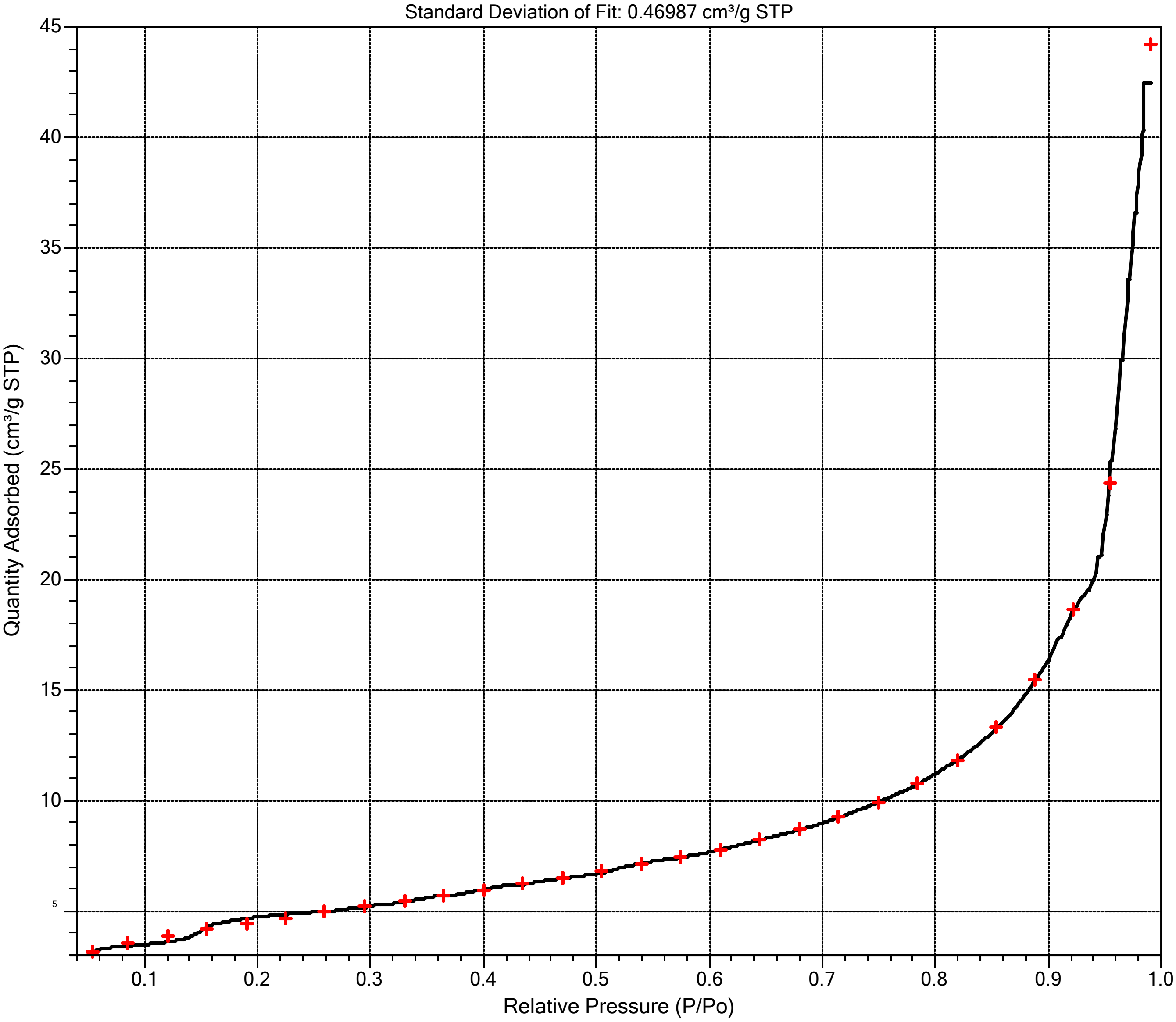

Sample: zmb-bjh
Operator:
Submitter:
File: E:\ASAP 2460\data\2307267899-1.SMP

Started:	2023/7/30 8:14:01	Analysis adsorptive:	N_2
Completed:	2023/7/30 11:44:14	Analysis bath temp.:	-195.850 °C
Report time:	2023/7/30 19:22:42	Thermal correction:	No
Sample mass:	0.1315 g	Ambient free space:	17.2326 cm^3 Measured
Analysis free space:	48.6379 cm^3	Equilibration interval:	6 s
Low pressure dose:	None	Sample density:	1.000 g/cm^3
Automatic degas:	No		

Porosity Distribution by Invalid

Model: N_2 - DFT Model
Method: Non-negative Regularization: 0.31600
Standard Deviation of Fit: 0.19312 cm^3/g STP

Volume in Pores	<	2.002 nm	0.00131 cm^3/g
Total Volume in Pores	<=	147.596 nm	0.06638 cm^3/g
Area in Pores	>	147.596 nm	0.188 m^2/g
Total Area in Pores	>=	2.002 nm	8.024 m^2/g

Pore Table

Pore Width (nm)	Cumulative Pore Volume (cm^3/g)	Incremental Pore Volume (cm^3/g)	Cumulative Pore Area (m^2/g)	Incremental Pore Area (m^2/g)
2.002	0.00145	0.00014	0.139	0.139
2.162	0.00161	0.00017	0.294	0.155
2.341	0.00182	0.00020	0.468	0.173
2.520	0.00206	0.00024	0.658	0.191
2.734	0.00234	0.00028	0.862	0.204
2.949	0.00265	0.00031	1.075	0.212
3.181	0.00299	0.00034	1.288	0.213
3.431	0.00335	0.00036	1.497	0.209
3.699	0.00372	0.00038	1.700	0.203
4.003	0.00412	0.00040	1.898	0.198
4.325	0.00454	0.00042	2.094	0.196
4.664	0.00500	0.00046	2.290	0.197
5.040	0.00550	0.00050	2.490	0.200
5.433	0.00605	0.00055	2.693	0.203
5.880	0.00666	0.00060	2.898	0.205
6.344	0.00731	0.00065	3.104	0.206
6.845	0.00802	0.00071	3.310	0.206
7.399	0.00878	0.00077	3.518	0.207
7.988	0.00962	0.00084	3.728	0.210
8.632	0.01054	0.00092	3.940	0.212
9.311	0.01152	0.00098	4.150	0.210
10.061	0.01254	0.00102	4.353	0.203
10.866	0.01359	0.00105	4.547	0.194
11.723	0.01469	0.00110	4.735	0.187
12.653	0.01586	0.00117	4.919	0.184
13.671	0.01711	0.00125	5.102	0.183
14.761	0.01844	0.00133	5.282	0.181
15.941	0.01985	0.00141	5.460	0.177
17.210	0.02130	0.00145	5.628	0.168
18.586	0.02266	0.00136	5.774	0.146
20.069	0.02379	0.00113	5.887	0.113
21.660	0.02464	0.00085	5.966	0.079

Sample: zmb-bjh
Operator:
Submitter:
File: E:\ASAP 2460\data\2307267899-1.SMP

Started: 2023/7/30 8:14:01
Completed: 2023/7/30 11:44:14
Report time: 2023/7/30 19:22:42
Sample mass: 0.1315 g
Analysis free space: 48.6379 cm³
Low pressure dose: None
Automatic degas: No

Analysis adsorptive: N2
Analysis bath temp.: -195.850 °C
Thermal correction: No
Ambient free space: 17.2326 cm³ Measured
Equilibration interval: 6 s
Sample density: 1.000 g/cm³

Pore Table

Pore Width (nm)	Cumulative Pore Volume (cm³/g)	Incremental Pore Volume (cm³/g)	Cumulative Pore Area (m²/g)	Incremental Pore Area (m²/g)
23.393	0.02544	0.00079	6.033	0.068
25.252	0.02655	0.00111	6.122	0.088
27.271	0.02822	0.00167	6.244	0.122
29.451	0.03046	0.00224	6.396	0.152
31.792	0.03336	0.00290	6.578	0.182
34.330	0.03686	0.00350	6.782	0.204
37.064	0.04040	0.00354	6.973	0.191
40.031	0.04344	0.00304	7.125	0.152
43.230	0.04578	0.00234	7.234	0.108
46.679	0.04787	0.00209	7.323	0.090
50.396	0.05016	0.00228	7.414	0.091
54.417	0.05231	0.00215	7.493	0.079
58.760	0.05397	0.00166	7.550	0.057
63.442	0.05543	0.00146	7.596	0.046
68.499	0.05699	0.00156	7.641	0.046
73.968	0.05827	0.00127	7.676	0.034
79.865	0.05941	0.00114	7.704	0.029
86.245	0.06071	0.00130	7.734	0.030
93.126	0.06192	0.00121	7.760	0.026
100.560	0.06279	0.00088	7.778	0.017
108.566	0.06358	0.00079	7.792	0.015
117.233	0.06449	0.00091	7.808	0.016
126.580	0.06520	0.00071	7.819	0.011
136.677	0.06575	0.00055	7.827	0.008
147.596	0.06638	0.00064	7.836	0.009

Sample: zmb-bjh
Operator:
Submitter:
File: E:\ASAP 2460\data\2307267899-1.SMP

Started:	2023/7/30 8:14:01	Analysis adsorptive:	N2
Completed:	2023/7/30 11:44:14	Analysis bath temp.:	-195.850 °C
Report time:	2023/7/30 19:22:42	Thermal correction:	No
Sample mass:	0.1315 g	Ambient free space:	17.2326 cm³ Measured
Analysis free space:	48.6379 cm³	Equilibration interval:	6 s
Low pressure dose:	None	Sample density:	1.000 g/cm³
Automatic degas:	No		

Porosity Distribution by Invalid
Model: N2 - DFT Model
Method: Non-negative Regularization: 0.31600
Standard Deviation of Fit: 0.19312 cm³/g STP

Isotherm Table

Relative Pressure (P/Po)	Experimental Quantity Adsorbed (cm³/g STP)	Fitted Quantity Adsorbed (cm³/g STP)	Absolute Residual (cm³/g STP)	Relative Residual
0.056084011	3.1872	3.1327	0.0545	0.017113
0.064206667	3.2875	3.2280	0.0596	0.018116
0.073040441	3.3917	3.3335	0.0582	0.017162
0.082588248	3.4969	3.4873	0.0096	0.002745
0.092847057	3.6021	3.6034	-0.0012	-0.000342
0.103808001	3.7099	3.7178	-0.0078	-0.002110
0.115456402	3.8187	3.8759	-0.0572	-0.014979
0.127772301	3.9277	3.9775	-0.0498	-0.012680
0.140730694	4.0399	4.0723	-0.0324	-0.008011
0.154301897	4.1535	4.2220	-0.0685	-0.016502
0.168452203	4.2684	4.3053	-0.0369	-0.008647
0.183144197	4.3848	4.4596	-0.0748	-0.017065
0.198337302	4.4999	4.5354	-0.0356	-0.007907
0.213988706	4.6164	4.6093	0.0071	0.001541
0.230053306	4.7344	4.7732	-0.0388	-0.008193
0.246484801	4.8544	4.8450	0.0094	0.001934
0.263235897	4.9754	4.9174	0.0580	0.011654
0.280259013	5.0965	5.0953	0.0012	0.000244
0.297506303	5.2198	5.1699	0.0499	0.009562
0.314930797	5.3455	5.3644	-0.0190	-0.003550
0.332486212	5.4720	5.4439	0.0282	0.005144
0.350127310	5.5985	5.5270	0.0714	0.012762
0.367810607	5.7262	5.7400	-0.0138	-0.002418
0.385494202	5.8637	5.8288	0.0349	0.005959
0.403138310	6.0013	5.9200	0.0813	0.013546
0.420704991	6.1326	6.1450	-0.0123	-0.002013
0.438158900	6.2654	6.2347	0.0308	0.004913
0.455466807	6.4016	6.4665	-0.0649	-0.010143
0.472598106	6.5378	6.5500	-0.0122	-0.001873
0.489524394	6.6732	6.6308	0.0424	0.006348
0.506219923	6.8107	6.8657	-0.0550	-0.008078
0.522661209	6.9545	6.9402	0.0143	0.002058
0.538827300	7.1001	7.1878	-0.0877	-0.012348
0.554699600	7.2437	7.2580	-0.0143	-0.001979
0.570261598	7.3876	7.3277	0.0600	0.008117
0.585499227	7.5367	7.5934	-0.0567	-0.007522

Sample: zmb-bjh
Operator:
Submitter:
File: E:\ASAP 2460\data\2307267899-1.SMP

Started: 2023/7/30 8:14:01
Completed: 2023/7/30 11:44:14
Report time: 2023/7/30 19:22:42
Sample mass: 0.1315 g
Analysis free space: 48.6379 cm³
Low pressure dose: None
Automatic degas: No

Analysis adsorptive: N2
Analysis bath temp.: -195.850 °C
Thermal correction: No
Ambient free space: 17.2326 cm³ Measured
Equilibration interval: 6 s
Sample density: 1.000 g/cm³

Isotherm Table

Relative Pressure (P/Po)	Experimental Quantity Adsorbed (cm³/g STP)	Fitted Quantity Adsorbed (cm³/g STP)	Absolute Residual (cm³/g STP)	Relative Residual
0.600400090	7.6970	7.6621	0.0349	0.004529
0.614954293	7.8607	7.9525	-0.0918	-0.011681
0.629153311	8.0276	8.0222	0.0053	0.000666
0.642990828	8.1960	8.0939	0.1021	0.012456
0.656461716	8.3665	8.4132	-0.0467	-0.005587
0.669562697	8.5423	8.4869	0.0554	0.006486
0.682291925	8.7224	8.8342	-0.1118	-0.012823
0.694648683	8.9137	8.9079	0.0058	0.000651
0.706633508	9.1135	8.9815	0.1320	0.014489
0.718248010	9.3149	9.3520	-0.0371	-0.003987
0.729494929	9.5223	9.4210	0.1014	0.010645
0.740377605	9.7345	9.8191	-0.0845	-0.008685
0.750900388	9.9478	9.8826	0.0652	0.006551
0.761068285	10.1708	10.3145	-0.1436	-0.014123
0.770887017	10.4047	10.3736	0.0311	0.002991
0.780362606	10.6427	10.8449	-0.2023	-0.019008
0.789501607	10.8833	10.9013	-0.0179	-0.001648
0.798311174	11.1415	10.9579	0.1836	0.016475
0.806798697	11.4102	11.4631	-0.0529	-0.004635
0.814971626	11.6809	11.5193	0.1617	0.013840
0.822837889	11.9507	12.0523	-0.1017	-0.008507
0.830405474	12.2386	12.1090	0.1296	0.010587
0.837682605	12.5408	12.6664	-0.1256	-0.010014
0.844677329	12.8499	12.7227	0.1271	0.009895
0.851397991	13.1597	13.3080	-0.1484	-0.011273
0.857852995	13.4731	13.3618	0.1113	0.008261
0.864050388	13.8187	13.9842	-0.1655	-0.011977
0.869998574	14.1857	14.0338	0.1519	0.010708
0.875705481	14.5609	14.7016	-0.1407	-0.009661
0.881179392	14.9337	14.7469	0.1868	0.012511
0.886428118	15.2958	15.4629	-0.1671	-0.010924
0.891459525	15.6534	15.5050	0.1484	0.009481
0.896281302	16.0448	16.2686	-0.2238	-0.013948
0.900900900	16.4592	16.3090	0.1502	0.009126
0.905325770	16.8835	17.0976	-0.2140	-0.012677
0.909563184	17.3075	17.1373	0.1702	0.009836
0.913620114	17.7229	17.8900	-0.1671	-0.009427
0.917503417	18.1234	17.9297	0.1938	0.010693
0.921219707	18.5042	18.5674	-0.0631	-0.003412
0.924775481	18.8381	18.6064	0.2317	0.012298
0.928177178	19.0620	19.1015	-0.0395	-0.002073

Sample: zmb-bjh
Operator:
Submitter:
File: E:\ASAP 2460\data\2307267899-1.SMP

Started: 2023/7/30 8:14:01
Completed: 2023/7/30 11:44:14
Report time: 2023/7/30 19:22:42
Sample mass: 0.1315 g
Analysis free space: 48.6379 cm³
Low pressure dose: None
Automatic degas: No

Analysis adsorptive: N2
Analysis bath temp.: -195.850 °C
Thermal correction: No
Ambient free space: 17.2326 cm³ Measured
Equilibration interval: 6 s
Sample density: 1.000 g/cm³

Isotherm Table

Relative Pressure (P/Po)	Experimental Quantity Adsorbed (cm³/g STP)	Fitted Quantity Adsorbed (cm³/g STP)	Absolute Residual (cm³/g STP)	Relative Residual
0.931430817	19.2384	19.1385	0.0998	0.005188
0.934542298	19.4261	19.6034	-0.1774	-0.009131
0.937517405	19.6698	19.6379	0.0319	0.001620
0.940361619	20.0024	20.2779	-0.2755	-0.013772
0.943080306	20.4473	20.3102	0.1371	0.006705
0.945678592	21.0195	21.2611	-0.2416	-0.011495
0.948161721	21.7280	21.2919	0.4361	0.020072
0.950534225	22.5763	22.5657	0.0106	0.000468
0.952800930	23.5638	24.2182	-0.6543	-0.027769
0.954966187	24.6785	24.2462	0.4322	0.017514
0.957034409	25.7861	26.2524	-0.4663	-0.018082
0.959009588	26.8440	26.2784	0.5656	0.021068
0.960896015	27.8543	28.3165	-0.4622	-0.016593
0.962697208	28.8190	28.3391	0.4798	0.016649
0.964416981	29.7400	30.1023	-0.3622	-0.012180
0.966058910	30.6194	30.1214	0.4979	0.016262
0.967626274	31.4588	31.4892	-0.0304	-0.000966
0.969122529	32.2602	32.7182	-0.4580	-0.014198
0.970550597	33.0250	32.7336	0.2914	0.008824
0.971913695	33.7550	34.0792	-0.3242	-0.009605
0.973214507	34.4517	34.0935	0.3582	0.010398
0.974455774	35.1165	35.3709	-0.2544	-0.007245
0.975640416	35.7509	35.3845	0.3665	0.010251
0.976770699	36.3563	36.3771	-0.0209	-0.000574
0.977849126	36.9339	37.2572	-0.3234	-0.008756
0.978878021	37.4849	37.2689	0.2160	0.005763
0.979859591	38.0106	38.2094	-0.1988	-0.005229
0.980795979	38.5121	38.2196	0.2925	0.007596
0.981689274	38.9905	38.9901	0.0004	0.000011
0.982541502	39.4470	39.6841	-0.2371	-0.006011
0.983354270	39.8822	39.6928	0.1894	0.004749
0.984129488	40.2974	40.4860	-0.1886	-0.004679
0.984869003	40.6935	40.4945	0.1990	0.004889
0.985574186	41.0712	41.2339	-0.1628	-0.003963
0.986246824	41.4314	41.2422	0.1892	0.004566
0.986888289	41.7750	41.7811	-0.0061	-0.000146
0.987500012	42.1026	42.2688	-0.1662	-0.003947
0.988083303	42.4150	42.2759	0.1391	0.003279
0.988639593	42.7129	42.8407	-0.1278	-0.002993
0.989170074	42.9970	42.8473	0.1497	0.003482
0.989675879	43.2679	43.2858	-0.0179	-0.000414

Sample: zmb-bjh
Operator:
Submitter:
File: E:\ASAP 2460\data\2307267899-1.SMP

Started: 2023/7/30 8:14:01
Completed: 2023/7/30 11:44:14
Report time: 2023/7/30 19:22:42
Sample mass: 0.1315 g
Analysis free space: 48.6379 cm³
Low pressure dose: None
Automatic degas: No

Analysis adsorptive: N2
Analysis bath temp.: -195.850 °C
Thermal correction: No
Ambient free space: 17.2326 cm³ Measured
Equilibration interval: 6 s
Sample density: 1.000 g/cm³

Isotherm Table

Relative Pressure (P/Po)	Experimental Quantity Adsorbed (cm³/g STP)	Fitted Quantity Adsorbed (cm³/g STP)	Absolute Residual (cm³/g STP)	Relative Residual
0.990158200	43.5262	43.6278	-0.1016	-0.002334
0.990618110	43.7725	43.6344	0.1381	0.003155
0.991056621	44.0074	44.0337	-0.0263	-0.000598

Sample: zmb-bjh
Operator:
Submitter:
File: E:\ASAP 2460\data\2307267899-1.SMP

Started:	2023/7/30 8:14:01	Analysis adsorptive:	N2
Completed:	2023/7/30 11:44:14	Analysis bath temp.:	-195.850 °C
Report time:	2023/7/30 19:22:42	Thermal correction:	No
Sample mass:	0.1315 g	Ambient free space:	17.2326 cm³ Measured
Analysis free space:	48.6379 cm³	Equilibration interval:	6 s
Low pressure dose:	None	Sample density:	1.000 g/cm³
Automatic degas:	No		

Cumulative Surface Area vs. Pore Width

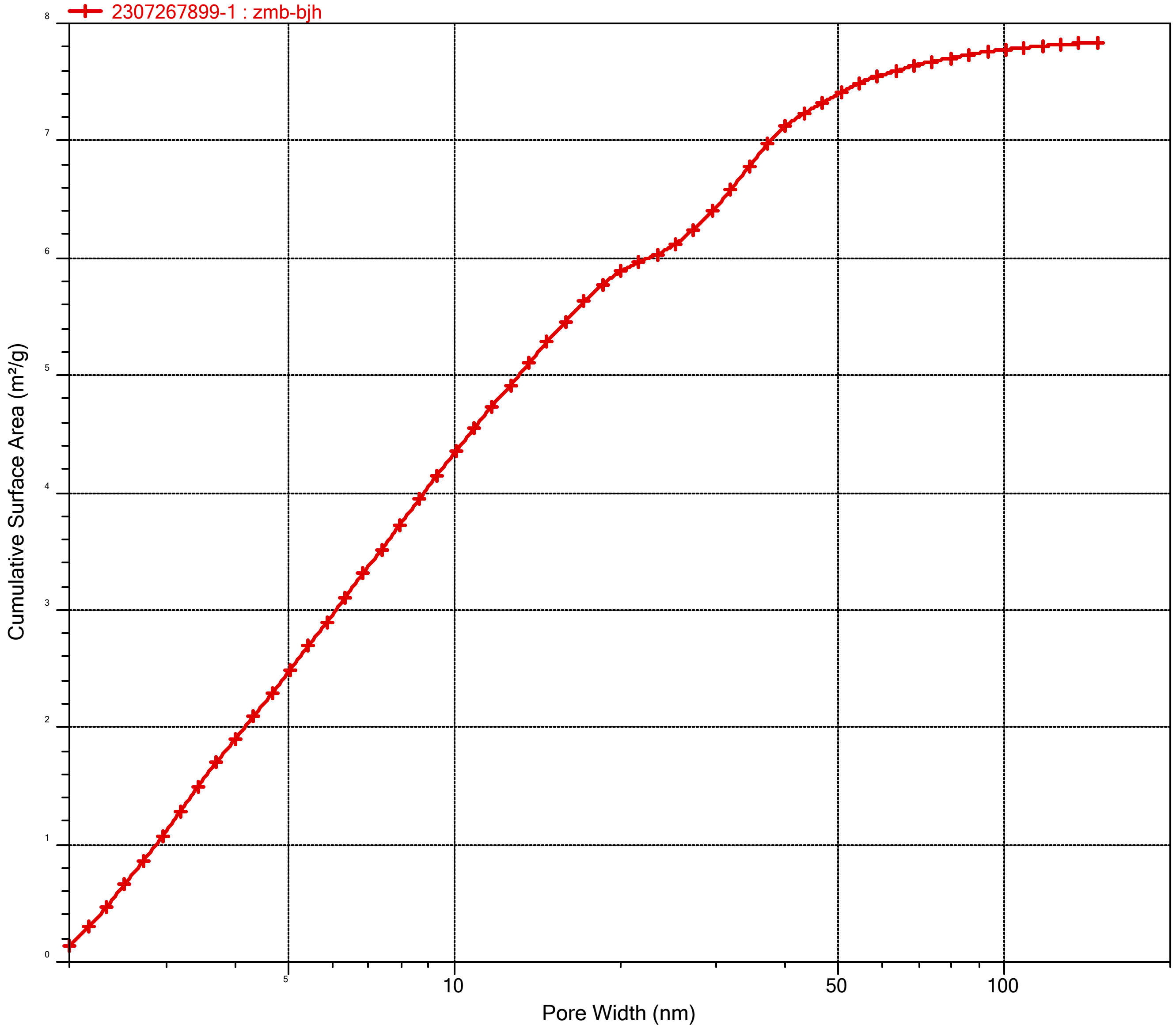

Sample: zmb-bjh
Operator:
Submitter:
File: E:\ASAP 2460\data\2307267899-1.SMP

Started:	2023/7/30 8:14:01
Completed:	2023/7/30 11:44:14
Report time:	2023/7/30 19:22:42
Sample mass:	0.1315 g
Analysis free space:	48.6379 cm³
Low pressure dose:	None
Automatic degas:	No

Analysis adsorptive:	N2
Analysis bath temp.:	-195.850 °C
Thermal correction:	No
Ambient free space:	17.2326 cm³ Measured
Equilibration interval:	6 s
Sample density:	1.000 g/cm³

Incremental Surface Area vs. Pore Width

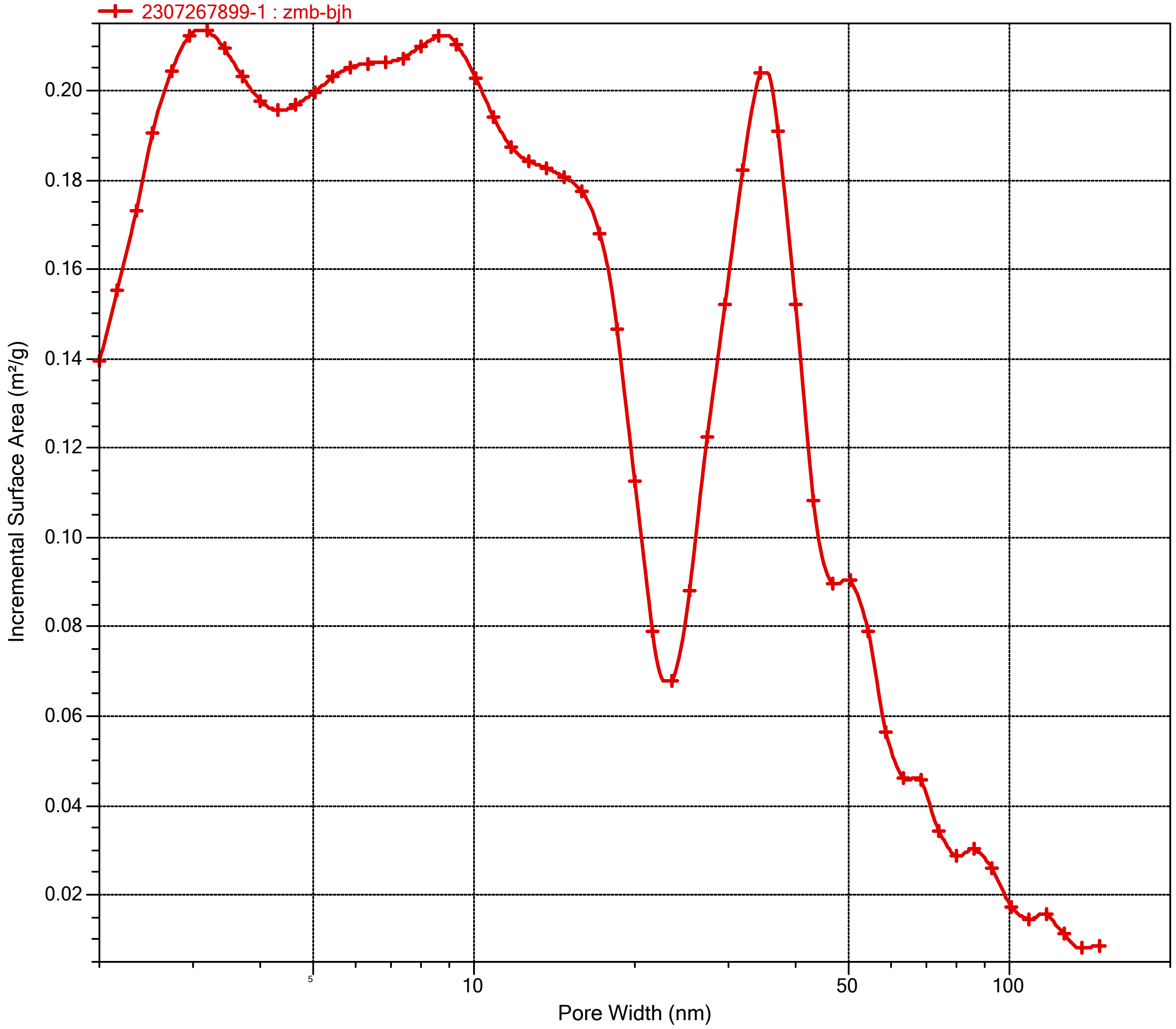

Sample: zmb-bjh
Operator:
Submitter:
File: E:\ASAP 2460\data\2307267899-1.SMP

Started:	2023/7/30 8:14:01	Analysis adsorptive:	N2
Completed:	2023/7/30 11:44:14	Analysis bath temp.:	-195.850 °C
Report time:	2023/7/30 19:22:42	Thermal correction:	No
Sample mass:	0.1315 g	Ambient free space:	17.2326 cm³ Measured
Analysis free space:	48.6379 cm³	Equilibration interval:	6 s
Low pressure dose:	None	Sample density:	1.000 g/cm³
Automatic degas:	No		

dA/dW Surface Area vs. Pore Width

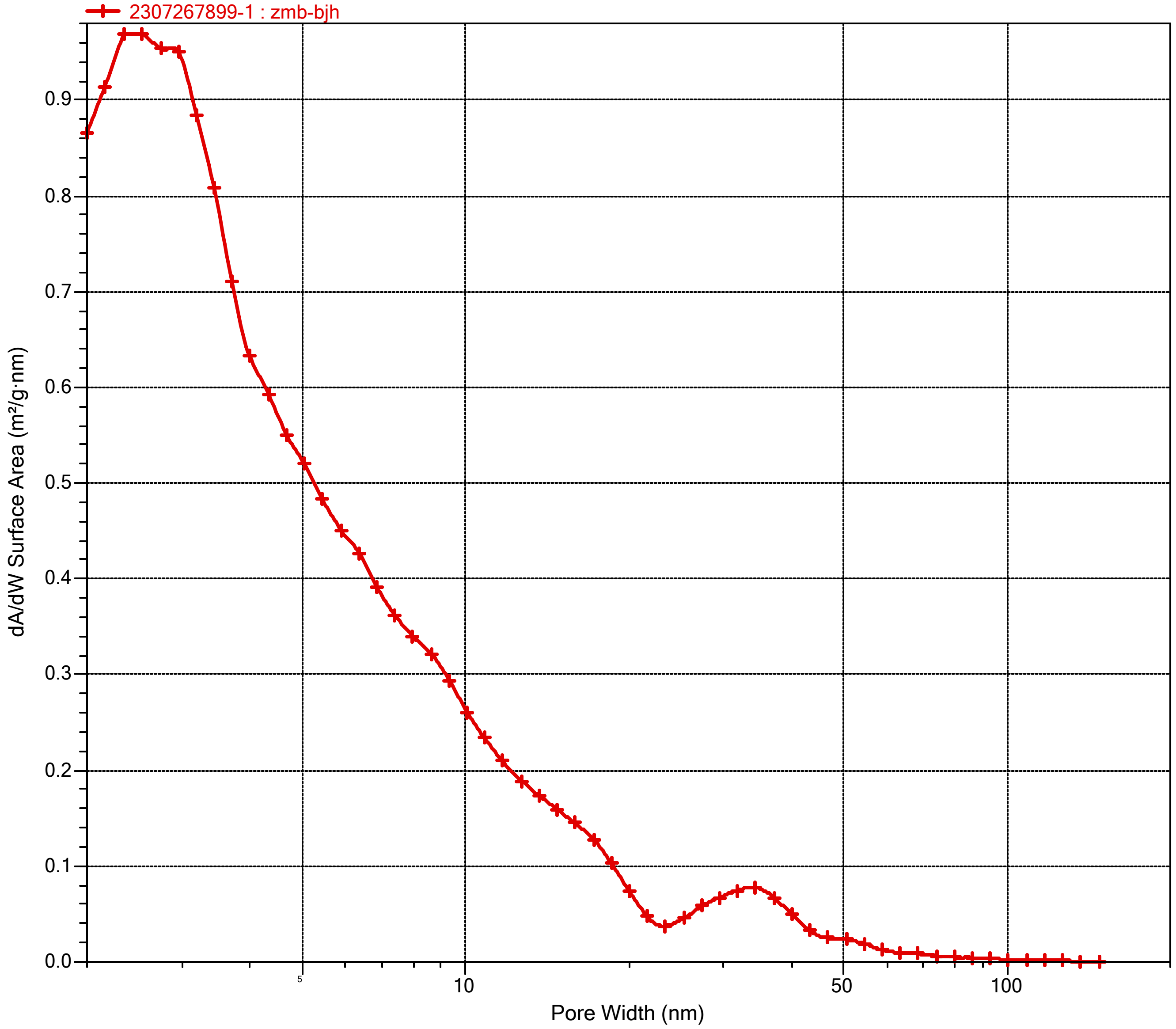

Sample: zmb-bjh
Operator:
Submitter:
File: E:\ASAP 2460\data\2307267899-1.SMP

Started:	2023/7/30 8:14:01	Analysis adsorptive:	N2
Completed:	2023/7/30 11:44:14	Analysis bath temp.:	-195.850 °C
Report time:	2023/7/30 19:22:42	Thermal correction:	No
Sample mass:	0.1315 g	Ambient free space:	17.2326 cm³ Measured
Analysis free space:	48.6379 cm³	Equilibration interval:	6 s
Low pressure dose:	None	Sample density:	1.000 g/cm³
Automatic degas:	No		

dA/dlog(W) Surface Area vs. Pore Width

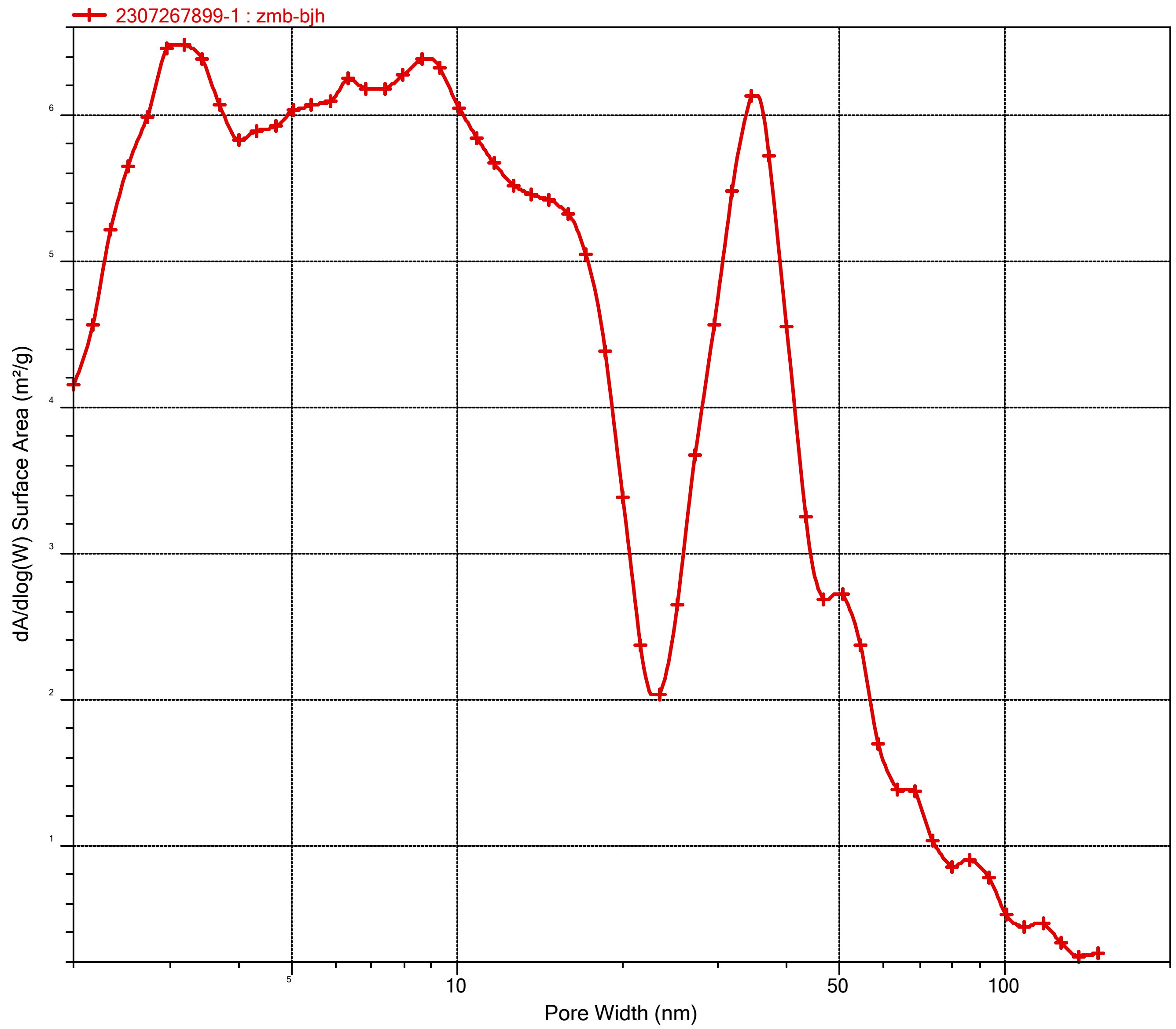

Sample: zmb-bjh
Operator:
Submitter:
File: E:\ASAP 2460\data\2307267899-1.SMP

Started:	2023/7/30 8:14:01	Analysis adsorptive:	N2
Completed:	2023/7/30 11:44:14	Analysis bath temp.:	-195.850 °C
Report time:	2023/7/30 19:22:42	Thermal correction:	No
Sample mass:	0.1315 g	Ambient free space:	17.2326 cm³ Measured
Analysis free space:	48.6379 cm³	Equilibration interval:	6 s
Low pressure dose:	None	Sample density:	1.000 g/cm³
Automatic degas:	No		

Cumulative Pore Volume vs. Pore Width

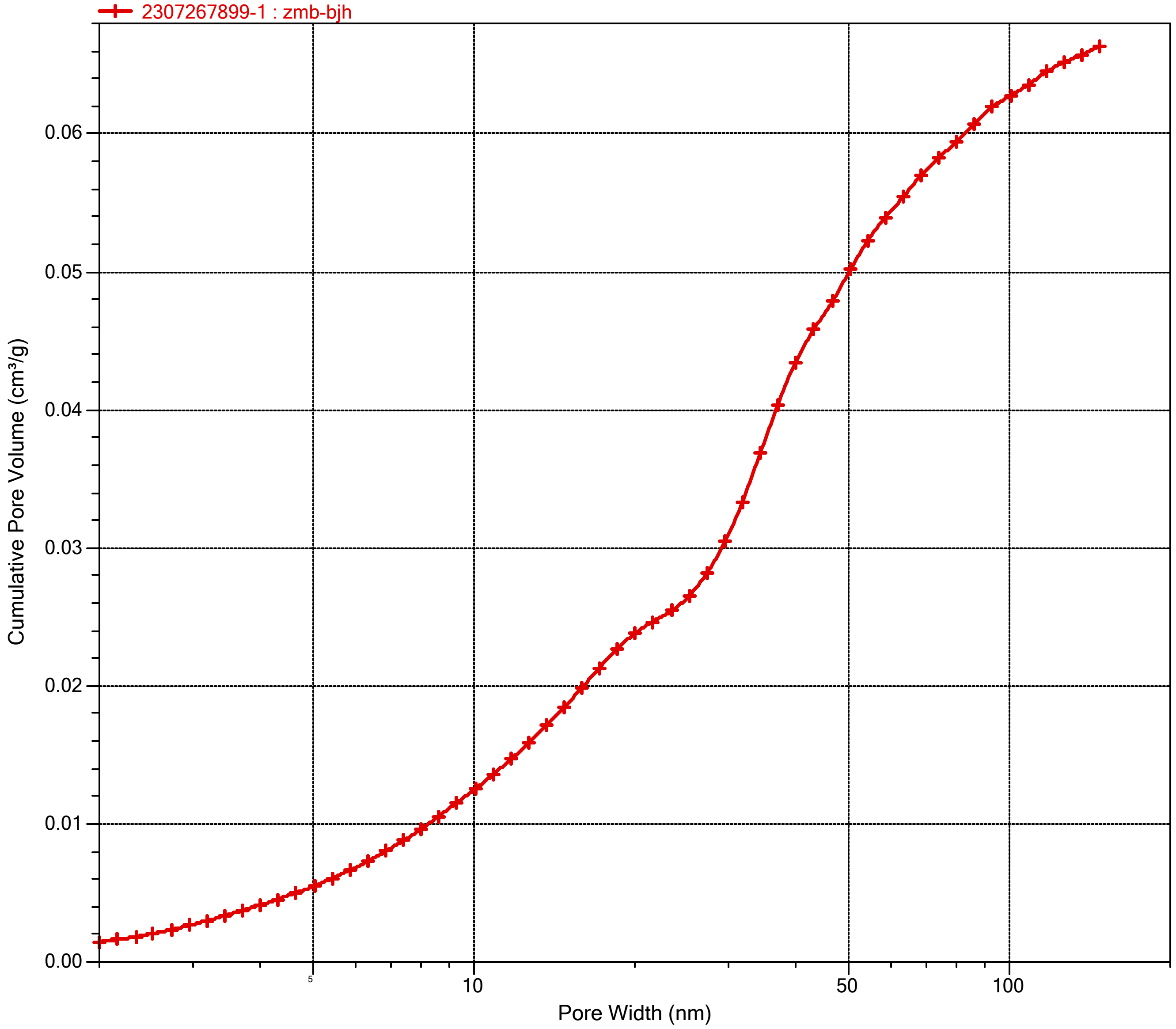

Sample: zmb-bjh
Operator:
Submitter:
File: E:\ASAP 2460\data\2307267899-1.SMP

Started:	2023/7/30 8:14:01	Analysis adsorptive:	N2
Completed:	2023/7/30 11:44:14	Analysis bath temp.:	-195.850 °C
Report time:	2023/7/30 19:22:42	Thermal correction:	No
Sample mass:	0.1315 g	Ambient free space:	17.2326 cm³ Measured
Analysis free space:	48.6379 cm³	Equilibration interval:	6 s
Low pressure dose:	None	Sample density:	1.000 g/cm³
Automatic degas:	No		

Incremental Pore Volume vs. Pore Width

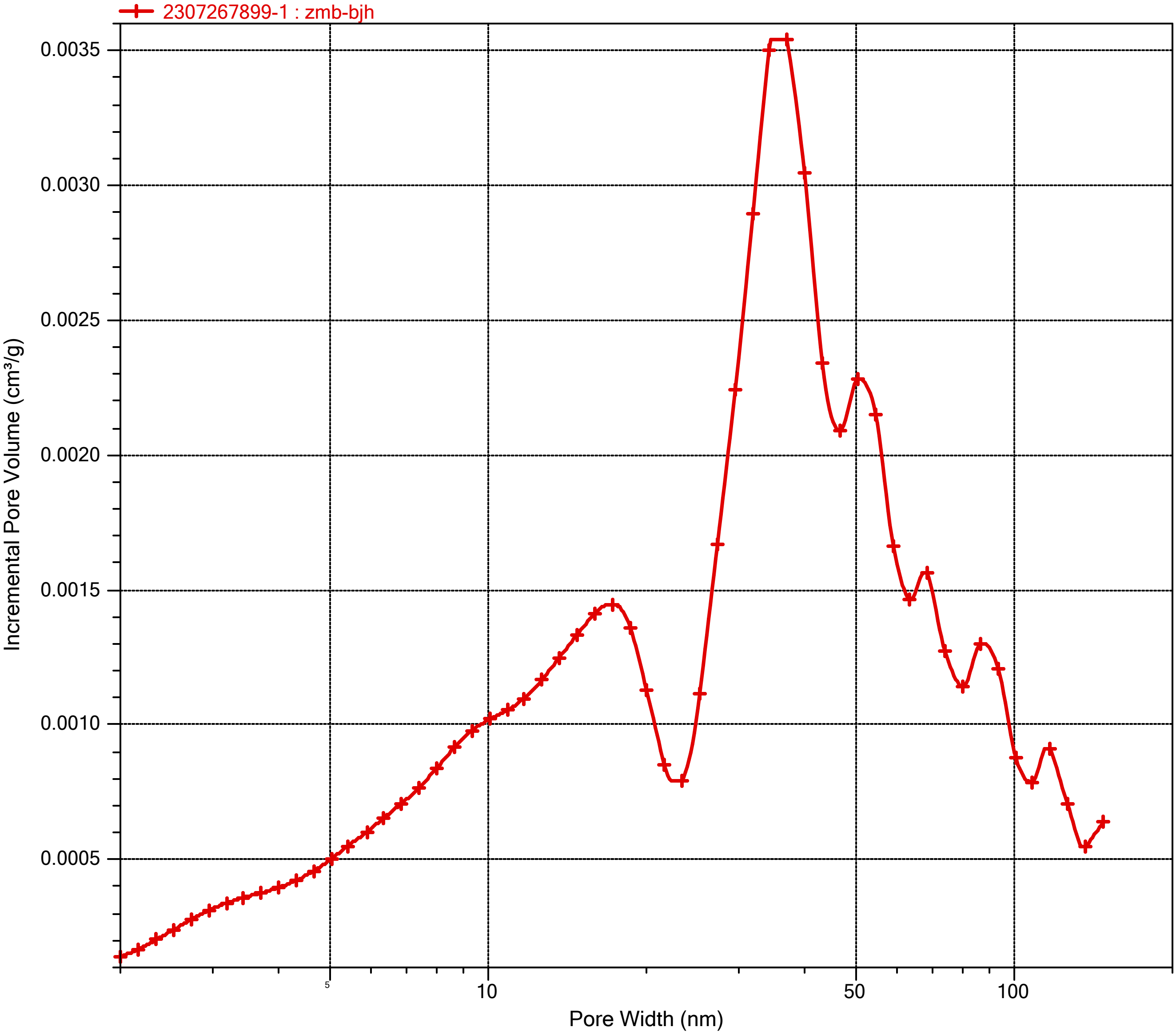

Sample: zmb-bjh
Operator:
Submitter:
File: E:\ASAP 2460\data\2307267899-1.SMP

Started:	2023/7/30 8:14:01	Analysis adsorptive:	N2
Completed:	2023/7/30 11:44:14	Analysis bath temp.:	-195.850 °C
Report time:	2023/7/30 19:22:42	Thermal correction:	No
Sample mass:	0.1315 g	Ambient free space:	17.2326 cm³ Measured
Analysis free space:	48.6379 cm³	Equilibration interval:	6 s
Low pressure dose:	None	Sample density:	1.000 g/cm³
Automatic degas:	No		

dV/dW Pore Volume vs. Pore Width

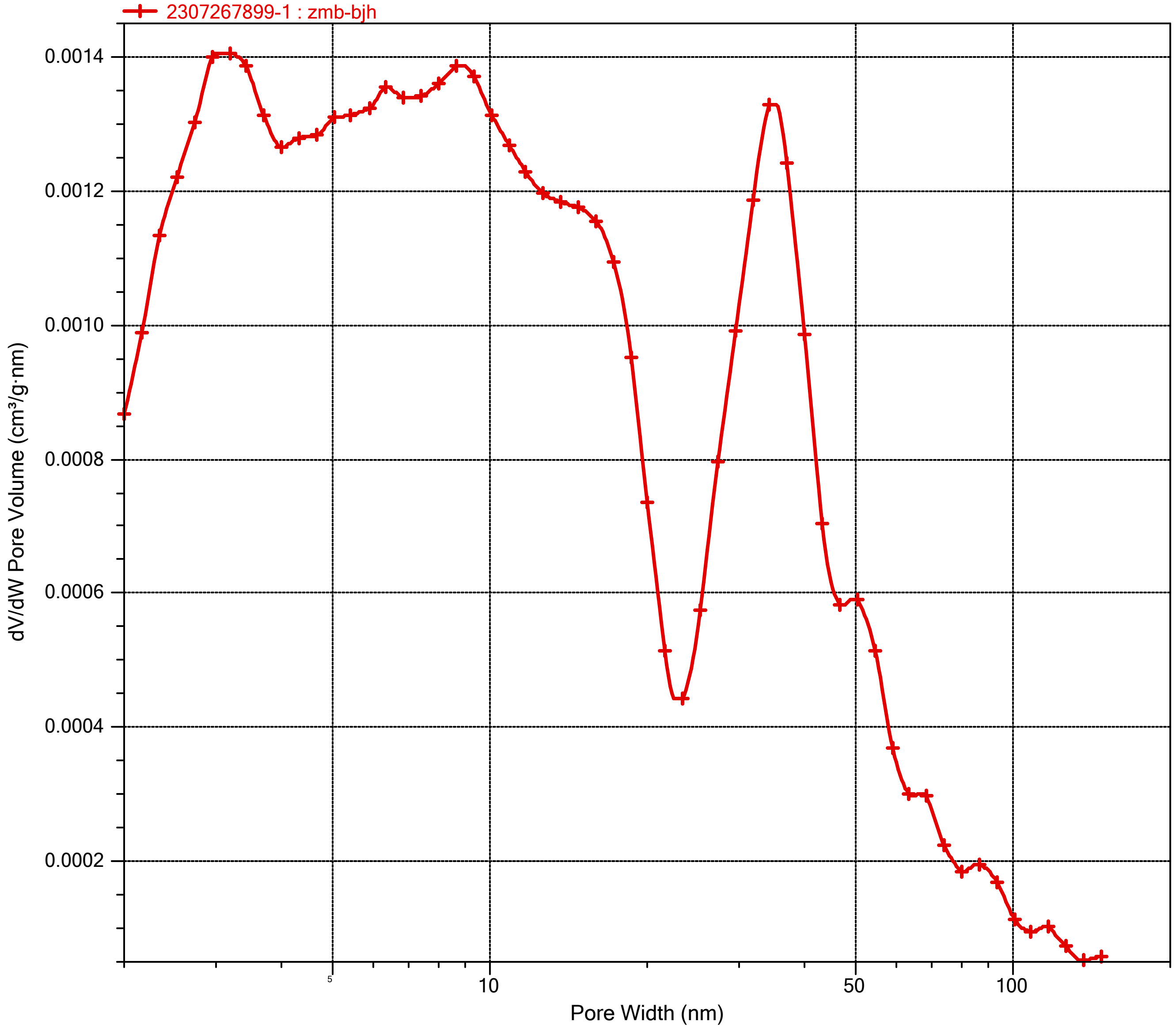

Sample:	zmb-bjh
Operator:	
Submitter:	
File:	E:\ASAP 2460\data\2307267899-1.SMP

Started:	2023/7/30 8:14:01	Analysis adsorptive:	N2
Completed:	2023/7/30 11:44:14	Analysis bath temp.:	-195.850 °C
Report time:	2023/7/30 19:22:42	Thermal correction:	No
Sample mass:	0.1315 g	Ambient free space:	17.2326 cm³ Measured
Analysis free space:	48.6379 cm³	Equilibration interval:	6 s
Low pressure dose:	None	Sample density:	1.000 g/cm³
Automatic degas:	No		

dV/dlog(W) Pore Volume vs. Pore Width

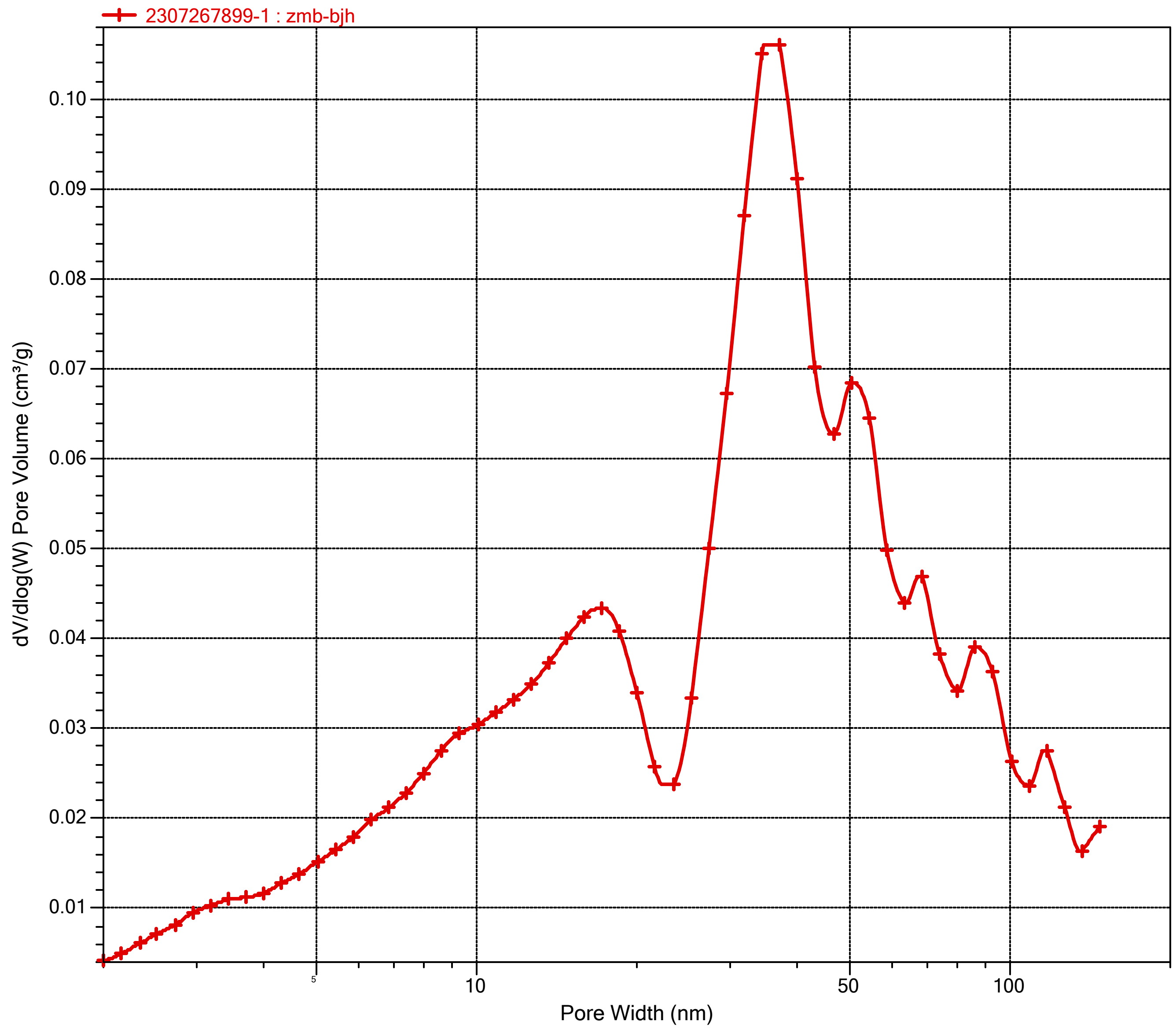

Sample: zmb-bjh
Operator:
Submitter:
File: E:\ASAP 2460\data\2307267899-1.SMP

Started:	2023/7/30 8:14:01	Analysis adsorptive:	N2
Completed:	2023/7/30 11:44:14	Analysis bath temp.:	-195.850 °C
Report time:	2023/7/30 19:22:42	Thermal correction:	No
Sample mass:	0.1315 g	Ambient free space:	17.2326 cm³ Measured
Analysis free space:	48.6379 cm³	Equilibration interval:	6 s
Low pressure dose:	None	Sample density:	1.000 g/cm³
Automatic degas:	No		

Goodness of Fit

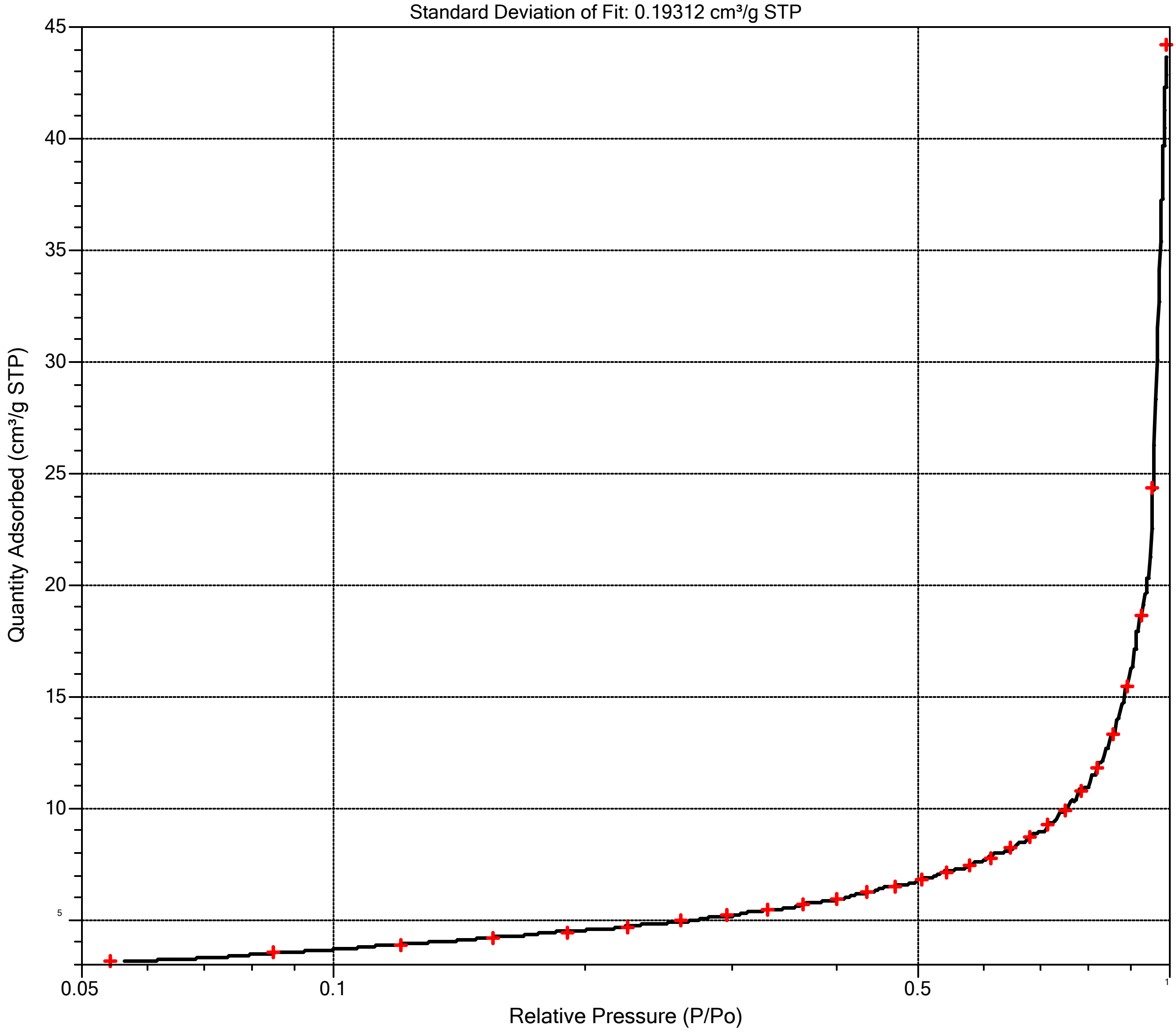

Sample: zmb-bjh
Operator:
Submitter:
File: E:\ASAP 2460\data\2307267899-1.SMP

Started:	2023/7/30 8:14:01	Analysis adsorptive:	N2
Completed:	2023/7/30 11:44:14	Analysis bath temp.:	-195.850 °C
Report time:	2023/7/30 19:22:42	Thermal correction:	No
Sample mass:	0.1315 g	Ambient free space:	17.2326 cm³ Measured
Analysis free space:	48.6379 cm³	Equilibration interval:	6 s
Low pressure dose:	None	Sample density:	1.000 g/cm³
Automatic degas:	No		

Goodness of Fit

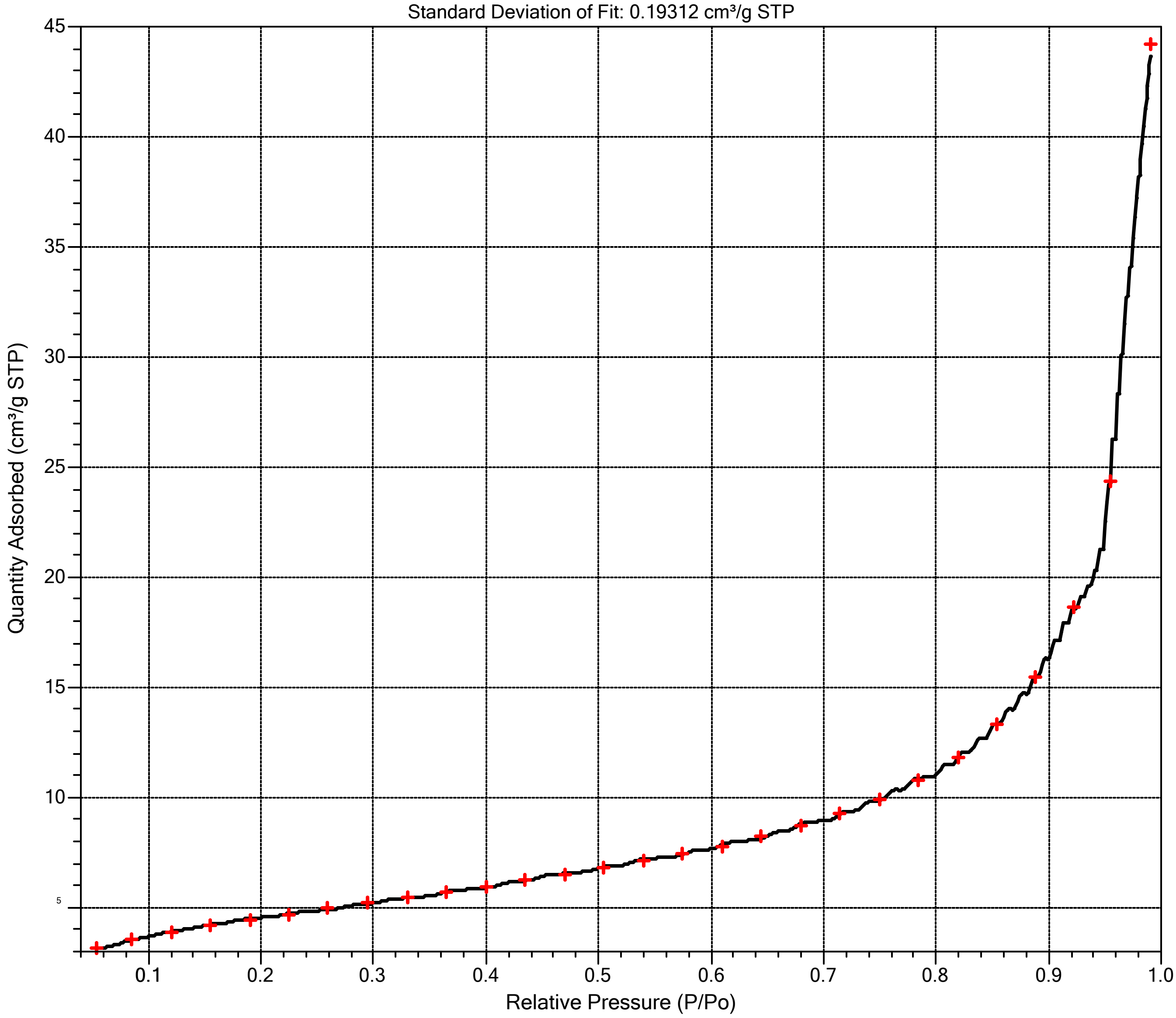

Supplement: S1 Data set — (ZIP) [file pone.0301986.s007.zip › minimal data set/▒φ╒≈╩2╛▌.zip!/▒φ╒≈╩2╛▌/BET/SBC500.zip!/2307267899/2307267899-1.PDF]
