# Supplementary material for: Adsorption of phosphate over a novel magnesium-loaded sludge-based biochar
Source: PLoS One. 2024 Apr 16;19(4):e0301986. doi: 10.1371/journal.pone.0301986 (PMC11020854; doi:10.1371/journal.pone.0301986)
Supplement: S1 Data set — (ZIP) [file pone.0301986.s007.zip › minimal data set/▒φ╒≈╩2╛▌.zip!/▒φ╒≈╩2╛▌/FTIR/DESMgSBC500.zip!/pdf_20230802885740.pdf]

尊敬的科学指南针用户：

您好！

感谢您选择科学指南针，指南针自成立以来，在大家的信任和支持下不断前进，在专业服务科研人的道路上迈出一步步扎实的步伐。

科学指南针，秉承着科研资源开放共享的宗旨，一直在整合全国优质开放共享测试资源，为科研党们提供优质、高效的分析测试服务，从原有的材料测试，扩展到了生物实验外包、模拟计算、科研绘图、论文润色等各类科研服务。

其中100%硕士/博士学历的实验对接服务人员，是我们保障优质服务的信心所在；遍布全国的服务点和高校服务网络，让我们最合理地调配仪器资源。我们不断死磕自己，只为给科研人提供更好的服务，您为科研，我们为您！

为方便大家拿到数据后，更好的分析和处理数据，对本次测试的仪器及数据说明如下：

（说明：以下数据格式简述部分为各个测试项目的示例数据，只有用来举例说明，不是您的真实数据！您自己实际得到的数据请具体查看订单数据）

测试项目：傅立叶红外光谱（FTIR）

仪器型号：Thermo Scientific Nicolet iS5

数据格式简述：一般导出数据有以下几种类型：（不同型号导出数据有差异，请根据实际收到的数据对应相应的方式打开）

1. 用记事本打开的数据格式有：TXT，DPT；

2. 用excel打开的数据格式有：excel；

3. 可直接查看的PDF文档或图片数据；

4. 软件（例如omnic）打开的格式 ：SPA，PKCS

备注：

1. 岛津仪器一般只能导出文本打开的数据，文本打开作图后手动标峰处理。

2. 测量模式可在SPA/excel/图片/PDF中查询到，一般T为透过率，A为吸光度，一般数据第一列是波数（单位cm^{-1}），第二列是测试时选择的模式（吸收/透过）。

3. Dpt格式可直接用TXT打开：复制全部数据，粘贴在excel中，选中数据列，选择数据-分列-分隔符号-下一步-分隔符号-下一步-完成，即可将数据分成两列，可将两列数据复制到origin中作图。

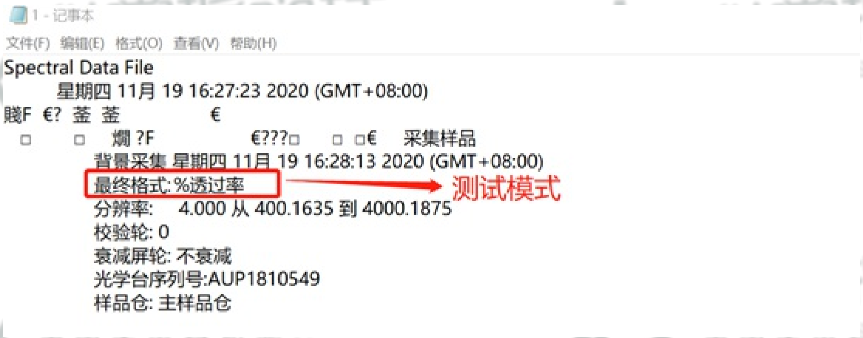

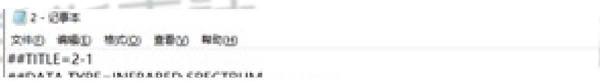

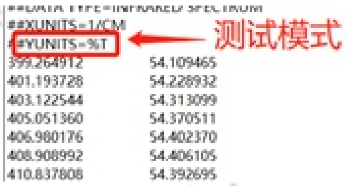


```
##DATA TYPE=INFRARED SPECTRUM
##XUNITS=1/CM
##YUNITS=%T
399.264912    54.109465
401.193728    54.228932
403.122544    54.313099
405.051360    54.370511
406.980176    54.402370
408.908992    54.406105
410.837808    54.392695
```

常用软件与资料下载

温馨提示：以下链接无法在PDF里直接点开，需要您把对应链接复制粘贴到浏览器的搜索栏里打开哦

红外光谱原理及谱图解析：

https://shiyanjia.oss-cn-hangzhou.aliyuncs.com/数据解读文档/FTIR/红外光谱原理及谱图解析.ppt

OMNIC软件：

https://shiyanjia.oss-cn-hangzhou.aliyuncs.com/数据解读文档/FTIR/红外软件OMNIC.zip

OMNIC软件使用指南：

https://shiyanjia.oss-cn-hangzhou.aliyuncs.com/数据解读文档/FTIR/OMNIC-中文用户指南.pdf

Origin软件：

https://shiyanjia.oss-cn-hangzhou.aliyuncs.com/数据解读文档/XRD/Origin 8 专业版破解版下载.zip

吸收模式和透过模式之间转换，及数据处理说明：

https://shiyanjia.oss-cn-hangzhou.aliyuncs.com/数据解读文档/FTIR/红外处理教程.pdf

常见问题与解答

Q：红外测试为保证数据质量，需注意以下事宜

1，样品必须预先纯化，以保证有足够的纯度；

2，样品须预先除水干燥，避免损坏仪器，同时避免水峰对样品谱图的干扰；

3，易潮解的样品，应放置在干燥器内；

4，对易挥发、升华、对热不稳定的样品，请用带密封盖或塞子的容器盛装并盖紧，同时必须注明；

5，对于有毒性和腐蚀性的样品，用户必须用密封容器装好。送样时必须分别在样品瓶标签的明显位置上注明

备注：红外测试无论哪种方法都需要样品干燥，尽量含有比较低的水分

Q：红外常规测试步骤

溴化钾压片测试方法(适用于粉末样品）

测试流程：取1~2mg粉末试样、200mg纯KBr研细均匀，置于模具中，在油压机上压成透明薄片，将样片放入红外光谱仪中测试，波数范围4000~400cm-1，扫描次数32，分辨率4cm-1。

ATR测试方法(适用于液体，薄膜，固体样品)：

测试流程：取少量样品置于金刚石ATR模块中，波数范围4000~400cm-1，扫描次数32，分辨率4cm-1，红外光谱仪测试。

Q：如何选择红外测试方法

红外测试一般主要分为溴化钾压片法和ATR，液体样品池方法

溴化钾压片方法适合粉末样品，此方法中涉及溴化钾带入的杂峰影响，所以我们一般选择扣除溴化钾背景和空气背景方法（具体方法客户可以指定），扣除溴化钾背景可以尽量避免溴化钾引入的杂峰（主要因为溴化钾极易吸水，羟基峰影响非常明显）

ATR方法适合各种固体，块状薄膜，液体等无法研磨成粉末样品，该方法优势是无其它杂质峰干扰，但是缺点为有些样品峰会比较弱。

液体样品池法，该方法设计比较少，一般适合于一些液体做定量测试

Q：样品涂层测试，测试方法和样品厚度的要求

样品的厚度或合适的样品量是很重要的，通常要求厚度10-50um，对于极性物质要求厚度小一些，对于非极性物质要求厚一些。

有些客户需要测试样品涂层，这种样品尽量采用溴化钾压片，具体就是人工把涂层表面刮下来进行溴化钾压片测试，不采取ATR的原因是因为红外光线会透过涂层可能测试到基地的信号就会造成数据干扰

Q：红外定量问题

红外很难做到定量，只能做到半定量和定性，因为测试过程中，样品和溴化钾比例大约100:1，首先1mg样品很难称量到特别准确，其次压片过程中也很难压到完全一样的厚度，所以定量比较难。

Q：ATR方法，数据峰型较差

1，atr测试，对样品要求比较高，适合薄膜类，材质不是很硬的样品，而且要求样品表面平整（atr是反射，如样品不平整，数据峰型会很差，atr一般是测试物质的表面，）

2，对于ATR测试效果不好的，建议换KBr压片测，KBr压片对样品要求比较低，测试波长范围可以从400开始，出的峰比ATR的相对好

Q：样品在3000-4000理论上是没有峰，但是测出来有峰

多出的3000-4000是羟基峰，也就是水峰，原因有以下：

1，天气潮样品吸水，

2，溴化钾压片测试，溴化钾会而且很容易吸水，所以羟基峰很难避免

Q：有些峰未出来或信号不强

1，给到的数据，有时会经过平滑和基线拉平简单处理，所以在平滑过程中可能会除掉一些小的杂质峰，如需要原始数据或有疑问，具体可跟对接老师沟通确认；

2，对于有些颜色较重的样品，特别是黑色样品，如果加多了会导致透过率比较差，加少了信号比较弱，所以一般黑色样品加的量不大，如果感觉峰比较弱或其他疑问，可及时跟对接老师沟通

Q：光谱图基线不平

1，压片时样品颗粒太大造成的；

2，样品本身性质的原因导致的；

3，背景图与样品图相差太大导致的

其它问题，如无法确定样品编号、缺少数据、测错样品等

以上如对实验结果有疑问，请尽快联系科学指南针工作人员或拨打400-831-0631，我们会尽快给您确认并解决，由于我们工作的失误给您带来的不便，我们深感抱歉！

不会分析数据？

科学指南针现隆重推出旗下数据分析团队“铅笔解析”，可承接科研测试类数据分析，包括但不限于XRD、XPS、FTIR、TEM、单晶XRD、NMR、电化学等数据分析，未知物成分剖析和大数据/人工智能分析服务，如需购买对应分析服务，可致电400-831-0631，或直接咨询业务负责人张老师13520903432（同微信）

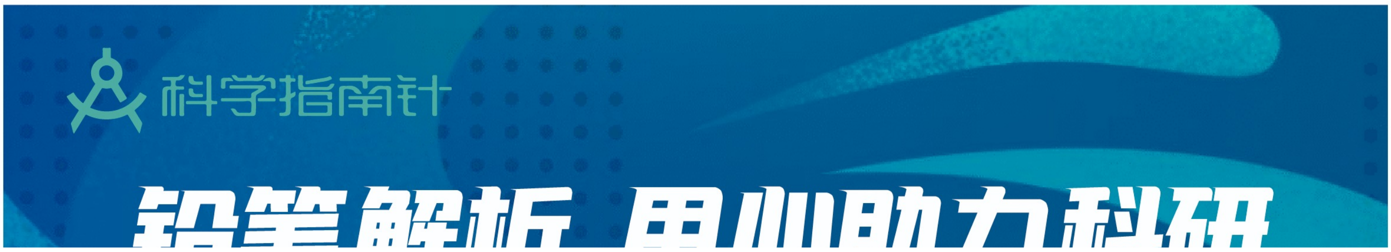

[illegible]

大类	分析项目名称	分析内容	报价	周期
材化类数据分析	XPS数据分析	全谱定性分析、分峰拟合、价态标定、含量分析、角分辨、mapping分析等	240/样起	2-7个工作日
	常规XRD数据分析	结晶度、晶粒尺寸、物相鉴定、物相鉴定+晶面标注、定量分析	150/样起	2-7个工作日
	XRD全岩+黏土分析	矿物成分定性定量分析、长石、浊沸石、蒙皂石类、伊利石、伊蒙混层、高岭石、绿泥石、绿蒙混层、蛭石、伊利石/蛭石混层 等等	600/样	2-7个工作日
	XRD精修	基础精修数据、COF结构精修、MOF结构精修、原子占位、氧空位、晶体结构三维图、键长键角、层间距、混排度、微观应变、应力分析等	300/样起	2-7个工作日
	中子衍射精修	晶体结构信息、轻原子位置、混排、及其他XRD精修无法处理的数据	一事一议	2-7个工作日
	单晶XRD数据分析	消除A类错误、消除B类错误、消除C类错误、解析结构	300/样起	2-7个工作日
	NMR数据分析	化学位移的归属、定量分析、耦合常数分析	200/样起	2-7个工作日
	小角散射数据分析	回转粒子半径、对距离分布函数、相关函数计算、Prood方程等	一事一议	2-7个工作日
	红外数据分析	标峰、标峰和对比分析、标峰和半定量分析、分峰计算、蛋白二级结构分析、主成分分析、二维红外分析等	200/样起	2-7个工作日
	圆二色谱（CD）数据分析	分峰拟合、蛋白质二级结构分析	250/样起	2-7个工作日
	TEM数据分析	高分辨晶相和晶面间距、衍射标定、粒径统计、原子距离量取、图片处理（高分辨降噪、局域傅里叶滤波、全图染色）、晶体模型建模、高分辨模拟	200/样起	2-7个工作日
	同步辐射吸收谱数据分析	EKR空间做图+XANES分析、EXAFS配位拟合、小波变换、全套分析、近边结构计算等	300/样起	2-7个工作日
	电化学数据分析	EIS拟合、赝电容贡献率计算、GITT数据分析等	150/样起	2-7个工作日
	EBSD数据分析	取向成像图、相图、极图（PF图）、CSL特殊晶界图、织构分布图等	100/样起	2-7个工作日
	穆斯堡尔谱分析	拟合谱图和参数表格（超精细场、同质异能移IS、四极分裂QS、半线宽Γ/2）	1500/样起	2-7个工作日
	质谱数据分析	裂解路径分析、解析得到产物结构、半定量分析	一事一议	5-10个工作日
	其他数据分析	BET数据分析、VSM数据分析、拉曼数据分析、EPR数据分析	200/样起	2-7个工作日
成分结构剖析	未知成分分析	未知溶液粉体成分定性定量分析、高分子材料剖析、工业原料剖析等	一事一议	7-14个工作日
	配方破解	商品配方破解、商品复配	一事一议	7-14个工作日
	失效分析	粘结失效、焊接失效、腐蚀失效、失效机理分析	一事一议	7-14个工作日
	未知结构鉴定	天然产物鉴定、有机合成产物鉴定	一事一议	7-14个工作日
科学计算	微积分	数值积分（计算谱图面积等）、求解常微分方程（组）	一事一议	2-7个工作日
	代数	求解（非）线性方程组	一事一议	2-7个工作日
	优化	求解最优化问题	一事一议	2-7个工作日
统计分析	统计分析	假设检验、计算协方差矩阵、相关系数矩阵等、	一事一议	2-7个工作日
	聚类	聚类分析	一事一议	2-7个工作日
	统计图表	绘制热力图、雷达图、等高线图等复杂图表	一事一议	2-7个工作日
人工智能	逻辑模型	利用最近邻，决策树，支持向量机等模型解决数值预测和类型识别问题	一事一议	2-7个工作日
	人工神经网络	利用人工神经网络模型解决数值预测和类型识别问题	一事一议	2-7个工作日

因分析需求不同，报价以最终确认为准

科学指南针 中国大型科研服务机构
科学指南针 中国大型科研服务机构

科研绘图，助力科研成果呈现

一张精美达意的图片，胜过千言万语，我们可帮助您把千辛万苦做出的实验结果以最靓丽的方式呈献给别人。封面图，TOC，插图，科研动画，通通都能帮您解决，是您的优秀成果不被埋没的重要辅助，助您实现科研成果的精彩绽放。如需绘图服务 ，可致电400-831-0631，或直接咨询业务负责人 王博 18222935002（同微信）

我们的优势

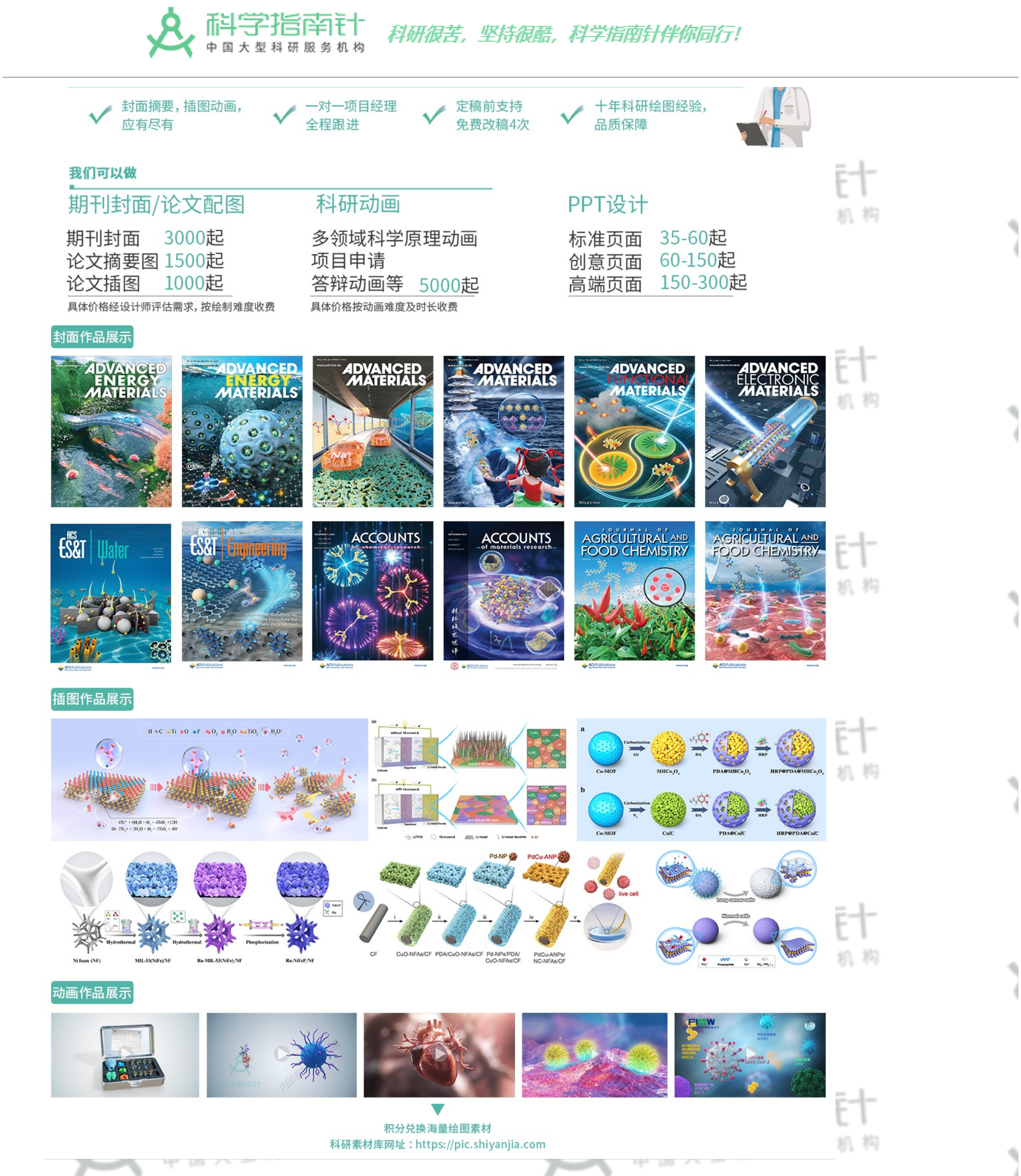

模拟仿真，助力论文档次提升

基于量子力学或牛顿力学的第一性原理、密度泛函理论（DFT）、分子动力学等方法，可以对物理、化学、材料、生物研究体系进行建模计算，从而得到其最优的几何构型、电子结构、热力学动力学等相关性质。科学指南针可提供几何构型、电子相关性质、动力/热力学相关性质、生物相关模拟、有限元模拟（comsol）相关的模拟仿真服务。如需模拟计算服务，可致电400-831-0631，或直接咨询业务负责人谢老师 15210358385（同微信）

分类	软件及适用方向	计算内容种类	具体可做内容（包括但不限于以下内容）
		分子结构分析	基态、激发态、过渡态结构，键长键角二面角等
		[illegible]	[illegible]

量子化学	**软件**：ORCA，Gaussian等<br>**适用方向**：有机、高分子等	分子性质计算	轨道分析（HOMO & LUMO等），电荷分布，键级，偶极矩，极化率，静电势，芳香性等
		光谱预测、解释和指认	红外，紫外，拉曼，圆二色谱，荧光/磷光，核磁等
		波函数分析	布居分析，键级分析，电子定域性、离域性分析，电荷分解分析，轨道成分分析，分子表面分析，密度差分析，定域化轨道分析，电子跃迁分析，AIM，NBO等
		热力学、动力学	反应热力学（焓 熵 自由能）、动力学（活化参数）等能量计算
		反应相关计算	分子反应性预测；反应机理的研究；过渡态搜索，反应路径计算，势能面扫描等
第一性原理	**软件**：VASP，MS等<br>**适用方向**：半导体、金属材料、非金属材料、纳米材料、催化、电池等	材料的微观结构及光、电、磁、力学及热力学相关的物理、化学性质等	结构优化，几何性质，DOS，差分电荷密度，ELF图，能带结构、费米能级，声子谱，声子态密度，载流子迁移率，扩散性质，迁移能垒，稳定性，导电性，磁性，吸附性质，催化性质，掺杂与缺陷形式，表面催化反应（包括但不限于HER, ORR, OER等）的反应路径和自由能，过渡态搜索等
分子模拟	**软件**：Gromacs，lammps等<br>**适用方向**：生物、生化、药物、医学、有机、高分子、材料、食品、环境等	建模	同源建模，从头建模
		分子对接	蛋白质-小分子"、"DNA-小分子"、"RNA-小分子"对接，蛋白-蛋白对接，蛋白-DNA等大分子对接
		三维结构分析	蛋白、DNA及RNA三维结构的预测，以及模拟蛋白、DNA及RNA等分子在不同温度、pH、电压等各种条件下结构变化
		弱相互作用分析	预测不同分子间结合自由能，分析药物结合大分子后对其二级结构的影响，分析蛋白结合口袋周围理化性质、小分子结合口袋周围亲疏水作用性质、氢键作用、静电作用、范德华力作用等弱相互作用模式分析
		分子动力学	高分子和溶液体系的分子动力学模拟，高分子构象预测和分析，溶液体系内粒子空间分布和相互作用，材料-溶液界面性质等
		药物相关	药物筛选，药物设计等
有限元模拟	**软件**：comsol，ansys，fluent，meshing，flotherm，aspen，adina，abaqus，factsage等<br>**适用方向**：流体、力学、材料、电磁、化工、土木、工艺等	材料分析	静力学分析，结构动力学，模态分析，随机振动分析，谐响应分析，屈曲分析，瞬态动力学分析，显示动力学分析，接触分析，复合材料分析，疲劳分析，传热分析，非线性分析，裂纹分析，二次开发等
		流体分析	导热，散热，流体流动与传热，自然对流与辐射换热，凝固融化，多相流，组分传输，动网格，相变，管道阻力，控制器、换热器分析等
		化工计算分析	塔（内件）的水力学计算，精馏（共沸物），塔规格，换热器，压缩机，闪蒸系统，蒸汽喷射式真空泵等
		相分析	二元、三元相图计算，化学反应平衡的计算，化学反应的热力学广延量（H,G,V,S,Cp,A）的变化计算等
因计算需求复杂度不同，所有需求报价均一事一议<br>以上相关模拟计算方向，经验丰富的有志之士，有意兼职者，欢迎联系加入，联系人（同微信）：谢老师（15210358385）			

杭州研趣信息技术有限公司
