# Supplementary material for: Adsorption of phosphate over a novel magnesium-loaded sludge-based biochar
Source: PLoS One. 2024 Apr 16;19(4):e0301986. doi: 10.1371/journal.pone.0301986 (PMC11020854; doi:10.1371/journal.pone.0301986)

Title:1

样品扫描次数: 32
背景扫描次数: 32
分辨率: 4.000
采样增益: 1.0
动镜速度: 0.4747
光阑: 100.00

检测器: DTGS KBr
分束器: KBr
光源: 红外光源

Comment:

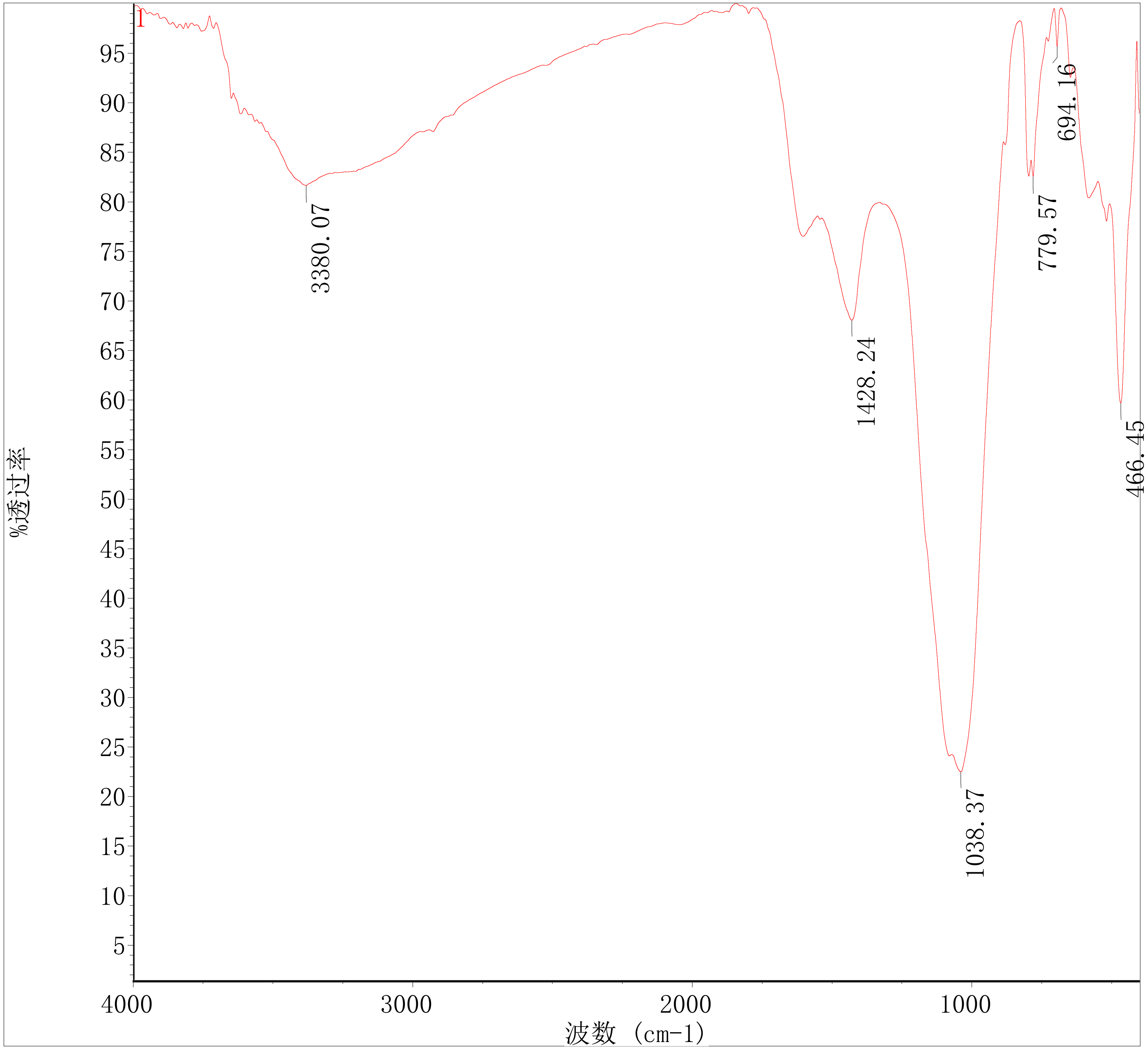

Supplement: S1 Data set — (ZIP) [file pone.0301986.s007.zip › minimal data set/▒φ╒≈╩2╛▌.zip!/▒φ╒≈╩2╛▌/FTIR/SBC500+PMgSBC500.zip!/2307260572/1.pdf]
