# Supplementary material for: Adsorption of phosphate over a novel magnesium-loaded sludge-based biochar
Source: PLoS One. 2024 Apr 16;19(4):e0301986. doi: 10.1371/journal.pone.0301986 (PMC11020854; doi:10.1371/journal.pone.0301986)

Title:2

样品扫描次数:　32
背景扫描次数:　32
分辨率:　 4.000
采样增益:　1.0
动镜速度:　0.4747
光阑:　100.00

检测器:　DTGS KBr
分束器:　KBr
光源:　红外光源

Comment:

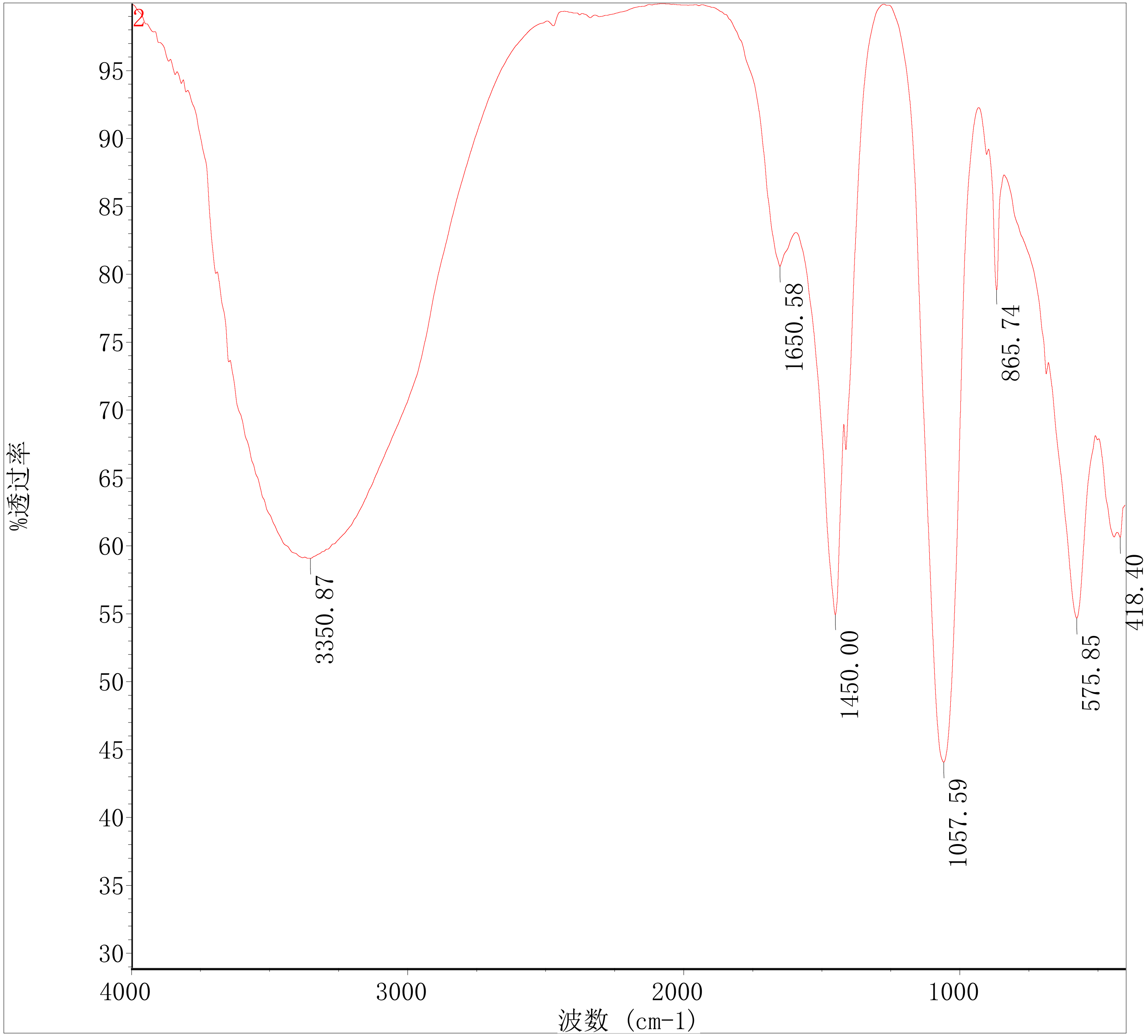

Supplement: S1 Data set — (ZIP) [file pone.0301986.s007.zip › minimal data set/▒φ╒≈╩2╛▌.zip!/▒φ╒≈╩2╛▌/FTIR/SBC500+PMgSBC500.zip!/2307260572/2.pdf]
