# Supplementary material for: Adsorption of phosphate over a novel magnesium-loaded sludge-based biochar
Source: PLoS One. 2024 Apr 16;19(4):e0301986. doi: 10.1371/journal.pone.0301986 (PMC11020854; doi:10.1371/journal.pone.0301986)
Supplement: Attachment — Submitted filename: Response to Reviewers.dox.docx [file pone.0301986.s009.docx]

**Response to Reviewer 2’s comments**

1. In the second paragraph of the Introduction, the sentence "The most typical way was to alter biochar (BC) with metal ions using various methods such as prediction" has a different font size from the surrounding text, and it also lacks punctuation at the end with a missing period. There are two instances of this type of error in the document.

Thank you for your suggestion. We have revised this sentence and other similar issues in the manuscript.

2. The first sentence of the third paragraph in the Introduction states, "SBC is one of the best candidates for phosphorus adsorption in wastewater treatment plants, as it can low-cost obtain synthetic biochar from sewage sludge [1]." In this sentence, SBC, which refers to sludge-based biochar as mentioned earlier, is correctly identified as a top candidate for phosphorus adsorption in wastewater treatment plants. However, the clause following 'as' introduces ambiguity, as it implies SBC is a method that "can low-cost obtain synthetic biochar from sewage sludge," which could be confusing. It is suggested to revise this for clarity.

Thank you for your suggestion. We have changed this sentence to:

“SBC has the advantage of low cost and is one of the best adsorbents for phosphorus treatment in wastewater treatment plants.”

3. Equation (1) should include parentheses around C0-Ct to reflect the original intent of the formula, which is to calculate the change in concentration before determining the amount of phosphate adsorbed per unit of adsorbent, Qt. Moreover, the document does not adequately define Qt, only mentioning it as the amount of phosphate adsorbed, whereas it specifically refers to the amount of phosphate adsorbed per unit of adsorbent.

Thank you for your suggestion. However, it is not common for us to take (*C*_0_-*C*_t_) as a formula alone after consulting the literature, so we only explain its meaning in the text, not as an additional formula. We have changed this paragraph to:

“Where *Q*_t_ refers to the amount of phosphorus adsorbed by each unit of adsorbent at time t. Where *C*_0_ (mg·L^-1^) represents the initial phosphorus concentration, *C*_t_ (mg·L^-1^) represents the phosphorus concentration at time t, and (*C*_0_-*C*_t_) refers to the change of phosphorus concentration.”

4.In the first subsection of the "Results and Discussion" section titled "Characterization and morphology," the second paragraph states, "In terms of BET surface area (cm2·g-1) and pore volume (cm3·g-1), SBC had values of 17.76 and 0.037, while MgSBC-0.1 had values of 93.35 and 0.17, while MgSBC-0.1 had values of 93.35, 0.17, and 0.00." The last two sentences are repetitive.

Thank you for your suggestion. This paragraph has been changed to:

“In terms of BET surface area (cm^2^·g^-1^) and pore volume (cm^3^·g^-1^), SBC had values of 17.76 and 0.037, while MgSBC-0.1 had values of 93.35 and 0.17.”

5. In the section preceding "Mechanism of P capture," there is a sentence that reads, "Subsequently, upon increasing the dosage of MgSBC-0.1 from 0.33 g·L-1 to 4.00·L-1 there was observed an ascending pattern in the removal rate of total phosphorus inside the biogas slurry." There is a mistake in the units of measurement in the sentence.

Thank you for your suggestion. We have checked and unified the unit of measurement of this sentence as “g·L^-1^”.
